# Second-Line Antiretroviral Therapy for Children Living with HIV in Africa

**DOI:** 10.1056/NEJMoa2404597

**Published:** 2025-05-15

**Authors:** Victor Musiime, Mutsa Bwakura-Dangarembizi, Alexander J. Szubert, Vivian Mumbiro, Hilda A. Mujuru, Cissy M. Kityo, Abbas Lugemwa, Katja Doerholt, Chishala Chabala, Shafic Makumbi, Veronica Mulenga, Helen McIlleron, David Burger, Eva Natukunda, Clare Shakeshaft, Kyomuhendo Jovia Linda, Kusum Nathoo, Lara Monkiewicz, Ibrahim Yawe, Monica Kapasa, Mary Nyathi, Joyce Lungu, Bwendo Nduna, Wedu Ndebele, Annabelle South, Mwate Mwamabazi, Godfrey Musoro, Anna Griffiths, Khozya Zyambo, Rashidah Nazzinda, Kevin Zimba, Yingying Zhang, Simon Walker, Anna Turkova, A. Sarah Walker, Alasdair Bamford, Diana M Gibb, for the CHAPAS-4 Trial Team

**Affiliations:** 1 Makerere University, College of Health Sciences, School of Medicine, Department of Paediatrics and Child Health, Kampala, Uganda; 2 Joint Clinical Research Centre, Kampala, Uganda; 3 Faculty of Medicine and Health Sciences, University of Zimbabwe, Harare, Zimbabwe; 4 University of Zimbabwe Clinical Research Centre, Harare, Zimbabwe; 5 Medical Research Council Clinical Trials Unit at University College London, 90 High Holborn, London WC1V 6LJ, United Kingdom; 6 Joint Clinical Research Centre, Mbarara, Uganda; 7 University of Zambia, School of Medicine, Lusaka, Zambia; 8 Infectious Diseases Research Collaboration, Kampala, Uganda; 9 University of Cape Town, Department of Medicine, Cape Town, 7700, South Africa; 10 Radboudumc Institute for Medical Innovation, Radboud University Medical Center, 6525 GA Nijmegen, The Netherlands; 11 Joint Clinical Research Centre, Fort Portal, Uganda; 12 University Teaching Hospitals Children’s Hospital, Lusaka, Zambia; 13 Mpilo Central Hospital, Bulawayo, Zimbabwe; 14 Arthur Davison Children’s Hospital, Ndola, Zambia; 15 National University of Science and Technology (NUST), NUST Mpilo Hospital Complex, Bulawayo, Zimbabwe; 16 Centre for Health Economics, University of York, York, YO10 5DD, UK; 17 UCL Great Ormond Street Institute of Child Health, London, WC1N 1EH, UK

## Abstract

**Background:**

Children living with HIV have limited second-line antiretroviral therapy (ART) options.

**Methods:**

In this open-label trial children were randomised to tenofovir alafenamide fumarate (TAF)-based or standard-of-care (SOC: abacavir (ABC) or zidovudine (ZDV) plus lamivudine (3TC)) backbone; and factorially to second-line anchor drugs: dolutegravir (DTG), ritonavir-boosted darunavir (DRV/r), atazanavir (ATV/r), or lopinavir (LPV/r). Primary endpoint was week-96 viral load (VL)<400copies/mL, analysed using logistic regression (intention-to-treat), hypothesising TAF would be non-inferior to SOC (10% margin), DTG and DRV/r superior to LPV/r and ATV/r combined, and ATV/r non-inferior to LPV/r (12% margin).

**Results:**

Between 17/12/18 and 01/04/21, 919 children, median[IQR] 10[8-13] years, 497(54.1%) male, baseline VL 17,573copies/ml[5,549-55,700], CD4 count 669cells/mm^3^[413-971], weight-for-age Z-score -1.6[-2.4,-0.9] were randomised. At week-96 TAF/FTC was superior to SOC (adjusted difference [95% CI] VL<400copies/mL +6.3%[2.0%,10.6%],p=0.004), with no evidence this varied by ABC/3TC or ZDV/3TC. Growth was better with TAF/FTC vs. SOC, without excess weight-gain with any backbone/anchor combination (including DTG+TAF/FTC). Bone health was similar between backbone arms, irrespective of anchor drug. DTG was superior (+9.7%[+4.8%,+14.5%],p<0.001) to LPV/r and ATV/r arms combined; DRV/r was not superior (+5.6%[+0.3%,+11.0%],p=0.04 vs. multiple-comparison adjusted threshold p=0.03). ATV/r was non-inferior to LPV/r (+3.4%[-3.4%,+10.2%]). All arms except LPV/r showed age-appropriate growth. CD4 counts increased similarly in all arms for both randomisations. One child died; 29(3%) had serious adverse events without between-arm differences.

**Conclusions:**

Second-line ART including TAF/FTC and DTG are effective for children without evident safety concerns. DRV/r is also a good option. Further development of child-friendly TAF/FTC fixed-dose-combinations (±anchor) would increase ART options, reducing the paediatric drug access gap. (ISRCTN22964075)

Globally, more children living with HIV (CLHIV) are accessing first-line antiretroviral therapy (ART); coupled with increasing HIV viral load (VL) monitoring, numbers requiring second/subsequent-line ART following virological failure are also increasing.^1–3^ Most CLHIV live in Africa where, until recently, first-line non-nucleoside reverse transcriptase inhibitor (NNRTI)-based regimens were widely used. Following first-line NNRTI-based ART failure, guidelines recommend an anchor drug from a new class (ritonavir-boosted protease inhibitor (PI) or integrase inhibitor (INSTI)), plus two nucleos(t)ide reverse transcriptase inhibitors (NRTIs). Maximising effectiveness and tolerability while minimising toxicity is particularly important for children requiring life-long ART.^4^ Which backbone and anchor drugs are safest and most effective for paediatric second-line ART remains unclear.

A tenofovir disoproxil fumarate (TDF)-based backbone is recommended for first and second-line ART for adolescents >30kg; INSTI-based regimens including tenofovir demonstrate robust efficacy when compared to ritonavir-boosted PI-based regimens including zidovudine in adult second-line trials.^5–7^ However, concerns about bone and renal toxicity and lack of paediatric formulations limit paediatric TDF use.^8^ Tenofovir alafenamide (TAF), a tenofovir prodrug, has lower dosage and better renal/bone safety profiles than TDF ^9,10^ and a new paediatric TAF/emtricitabine(FTC) fixed-dose-combination (FDC) (15mg/120mg) has been developed (although not widely available). There are minimal data on TAF in African children; the first paediatric pharmacokinetic data showed tenofovir concentrations equivalent to those safe and effective in adults.^11^ Dolutegravir (DTG) is available in child-friendly formulations. Ritonavir-boosted PIs, although providing sustained VL suppression and high barrier to resistance,^6,7^ have formulation challenges.^12^ Lopinavir (LPV) is the only paediatric ritonavir co-formulated boosted PI but requires twice-daily dosing and is unpalatable; ritonavir-boosted darunavir (DRV/r) and atazanavir (ATV/r) are dosed once-daily but paediatric FDCs are unavailable and DRV/r is relatively costly.

CHAPAS-4 compared efficacy, safety and tolerability of different second-line anchor drugs combined with TAF-based or SOC backbone in African children aged 3-15 years.

## Methods

CHAPAS-4(ISRCTN22964075) was a randomised, open-label trial with a 2x4 factorial design. The trial was approved by ethics committees in Uganda, Zambia, Zimbabwe, and UK (protocol: www.mrcctu.ucl.ac.uk/studies/all-studies/c/chapas-4). Participants were recruited at six centres in three African countries: Uganda (Joint Clinical Research Centre (JCRC), Kampala; JCRC, Mbarara), Zambia (University Teaching Hospital, Lusaka; Arthur Davison Children’s Hospital, Ndola) and Zimbabwe (University of Zimbabwe Clinical Research Centre, Harare; Mpilo Central Hospital, Bulawayo).

Participants were CLHIV aged 3-15 years, weighing ≥14kg, receiving first-line NNRTI-based ART, with treatment failure according to WHO criteria (confirmed VL>1000 copies/ml (after adherence counselling) or immunological/clinical criteria for failure) and screening visit VL>400 copies/ml. Post-menarchal females required a negative pregnancy test. Guardians provided written informed consent, with additional assent from older children, according to national guidelines. Children were excluded if they had severe hepatic impairment (alanine aminotransferase (ALT) ≥5x upper limit of normal (ULN), or ALT ≥3xULN and bilirubin ≥2xULN, or clinical liver disease). Full study details can be found in the protocol at nejm.org.

Participants were randomised to one of two backbones (TAF/FTC or standard-of-care (SOC) (abacavir (ABC)/3TC or ZDV/3TC, whichever not used first-line)) and simultaneously to one of four anchor drugs (DTG, DRV/r, ATV/r, LPV/r). Randomisation was stratified by centre and first-line NRTI (ABC or ZDV). A computer-generated sequential randomisation list with variably sized permuted blocks was prepared by the trial statistician and incorporated securely into an online database. Allocation was concealed until eligibility was confirmed by local centre staff, who then randomised.

Participants were seen at screening, ART switch (week 0), 2, 6, 12 weeks and 12-weekly thereafter to at least week-96 (primary endpoint): extended follow-up continued until 02/02/2023. Children with tuberculosis at enrolment or during follow-up underwent regimen modification to account for rifampicin drug-drug interactions. Additional measures ensured participant follow-up during the COVID-19 pandemic (Supplementary appendix: Supplementary methods).

Primary outcome was VL <400 copies/ml at week-96 (death counted as failure). Secondary efficacy outcomes were VL <60 copies/ml (the lower limit at one site) and <1000 copies/ml at week-96, death/World Health Organisation (WHO) 3/4 events, changes in CD4 count/percentage, and genotypic resistance. Safety outcomes were grade 3/4, serious, and ART-modifying adverse events (AEs); and changes in total cholesterol, low-density lipoprotein (LDL), high-density lipoprotein (HDL), triglycerides, bilirubin and creatinine clearance (CrCl). Other outcomes included changes in weight-, height- and body mass index (BMI)-for-age and bone mineral density Z-scores.

An economic analysis considered costs which were estimated from the health-system perspective and included ART, clinic visits and hospital stays in 2022 US dollars, discounted at 3% per annum (Supplementary appendix).

For the backbone randomisation, assuming 80.0%-87.5% SOC achieved VL<400 copies/ml at week-96, 920 children provided ≥95% power to demonstrate TAF was non-inferior (10% margin) (two-sided alpha=0.05), assuming 2.5% loss-to-follow-up (reduced from 10% in original protocol). For the anchor randomisation, 920 children provided 88% power to demonstrate ATV/r was non-inferior (12% margin) to LPV/r (two-sided alpha=0.05), assuming 80% <400 copies/ml at week-96, and 89% power to detect 10% higher suppression in each of DTG and DRV/r than LPV/r and ATV/r combined (two-sided alpha=0.03; as multiple comparisons) (including 2.5% loss to follow-up). Margins reflect the clinical consensus and are within the range used in previous second-line treatment trials in adults (Supplementary appendix: methods). An independent data monitoring committee reviewed interim data four times using the Haybittle–Peto criterion (99.9% confidence interval).

Analyses were intention-to-treat using Stata (version 17.0). Primary endpoint analyses used logistic regression (adjusting for stratification factors), then marginal estimation of risk differences. For non-inferiority comparisons, secondary per-protocol analyses included children who received randomised backbone/anchor drug for >90% follow-up. For death/WHO 3/4 events, and grade 3/4 serious and ART-modifying AEs, groups were compared via Cox regression (unadjusted). Changes in continuous outcomes were analysed using Normal generalised estimating equations adjusting for visit, stratification factors and baseline (and interactions between these factors and visit), for an overall analysis of difference between groups over all visits (independent correlation; mean difference reported). 95% confidence intervals were not adjusted for multiple testing (Supplementary appendix: methods).

European Developing Country Clinical Trial Partnership (funder), and pharmaceutical companies donating additional funding (Gilead Sciences, Johnson and Johnson) and drugs (ViiV Healthcare, Gilead Sciences, Johnson and Johnson, CIPLA), did not participate in trial design, conduct or analysis.

## Results

**Figure 1:**
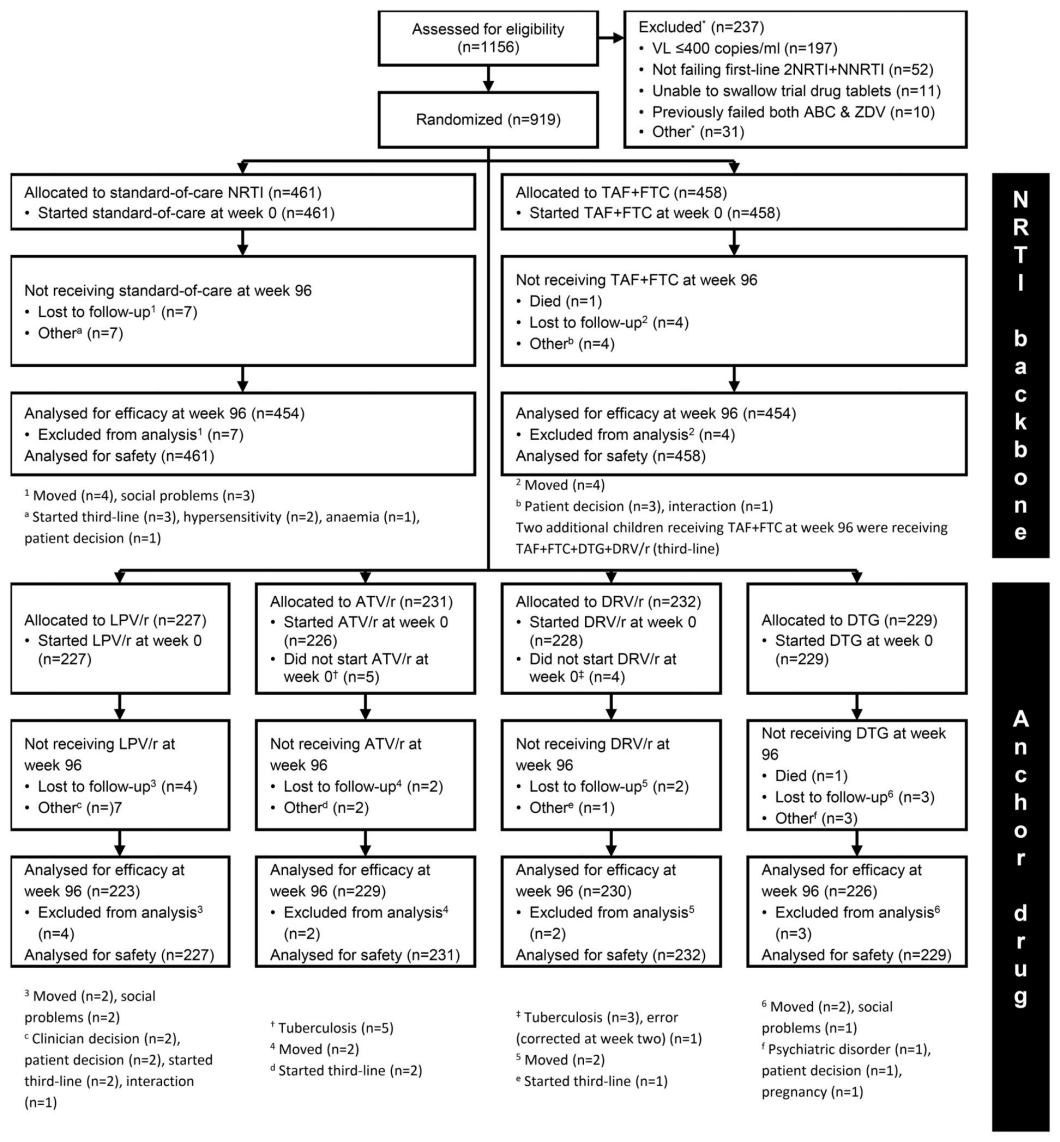
CONSORT flow diagram ABC denotes abacavir, ATV/r ritonavir-boosted atazanavir, DRV/r ritonavir-boosted darunavir, DTG dolutegravir, FTC emtricitabine, LPV/r ritonavir-boosted lopinavir, NNRTI non-nucleoside reverse transcriptase inhibitor, NRTI nucleoside/nucleotide reverse transcriptase inhibitor, TAF tenofovir alafenamide fumarate, VL HIV viral load, and ZDV zidovudine. * Reasons are not mutually exclusive therefore total to more than the total number of non-randomisations. Other reasons: declined to participate (n=7), did not return for enrolment within window (n=4), not aged 3-15 (n=4), biochemical (n=3), previously failed ritonavir-boosted lopinavir (n=2), contraception (n=1), contraindications (n=1), co-morbidities (n=1), died (n=1), other (n=9)

919 children were randomised between 17/12/2018 and 01/04/2021 (Figure 1). Baseline characteristics were similar between arms (Table 1; Table S3). 497(54.1%) children were male; median age 10 years (IQR 8,13); 777(84.5%) were WHO stage 1/2. Median weight-, height- and BMI-for-age Z-scores were between -1 and -1.6. Median VL was 17,573 copies/mL (IQR 5549,55700); CD4 count 669 cells/mm^3^ (413,971), CD4% 28%(19%,36%). Median time on first-line ART was 5.6 years (44% nevirapine, 56% efavirenz). Over 96 weeks, 98.9% of visits were attended. Eleven children (1.2%) were lost to follow-up. 674(73.3%) entered extended follow-up (median 60 (IQR 30,75) additional weeks).

### Backbone randomisation

In SOC, 217/461(47.1%) initiated ABC/3TC, 244(52.9%) ZDV/3TC. Prior to week-96, children spent 99.1% of time on allocated backbone (99.5% TAF/FTC vs. 98.8% SOC) and five (0.5%) initiated third-line ART (2(0.4%) TAF/FTC vs. 3(0.7%) SOC). In extended follow-up, children spent 93.5% of time on allocated backbone (95.6% TAF/FTC, 91.4% SOC) (Figure S1).

**Figure 2:**
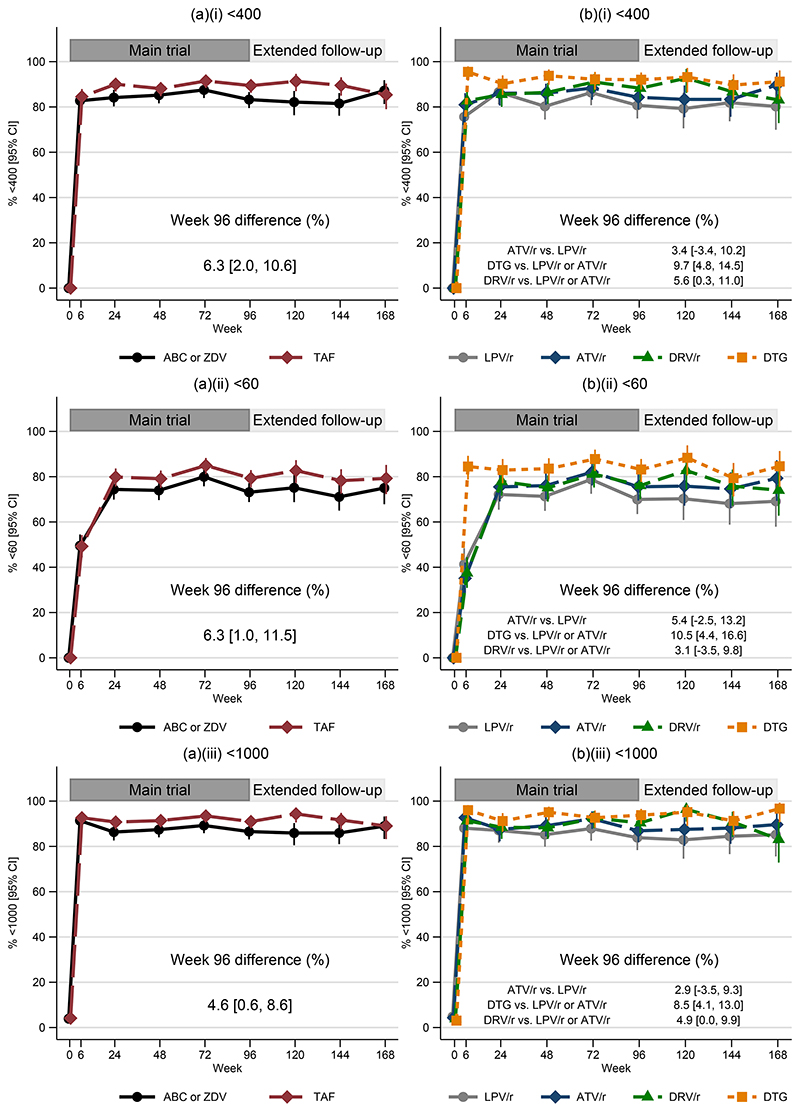
For the backbone (a) and anchor (b) randomisations, percentage of children with HIV viral load <400 copies/ml (i), <60 copies/ml (ii) and <1000 copies/ml (iii), over time during the main trial and during extended follow-up ABC denotes abacavir, ATV/r ritonavir-boosted atazanavir, DRV/r ritonavir-boosted darunavir, DTG dolutegravir, LPV/r ritonavir-boosted lopinavir, TAF tenofovir alafenamide fumarate and ZDV zidovudine

At week-96, 406/454(89.4%) TAF/FTC vs. 378/454(83.3%) SOC had VL <400copies/mL (adjusted difference +6.3% [95% confidence interval (CI) +2.0%,+10.6%]; p=0.004) (Figure 2). Therefore, TAF/FTC was non-inferior (and superior) to SOC according to the pre-specified 10% margin. There was no evidence of heterogeneity in the effect of TAF/FTC vs. SOC in any of 11 prespecified sub-groups (Figure S2), including first-line NRTI, anchor randomisation, country and baseline VL. Results of per-protocol analyses were similar: 403/449(89.8%) TAF/FTC vs. 370/445(83.1%) SOC had VL <400copies/mL (adjusted difference +6.8%[+2.4%,+11.1%]; p=0.002). Differences between arms in suppression <60 and <1000 copies/mL were similar between arms, as were results at weeks 48 and 144 (Table S4).

Over 96 weeks, 127/919(13.8%) children experienced 176 grade 3/4 AEs (63(13.8%) TAF/FTC vs. 64(13.9%) SOC) (Cox p=0.93) (Table 2; Table S6), including eight infections, all in SOC (4 malaria, 3 tuberculosis, 1 herpes zoster). Twenty-nine(3.2%) children experienced a total of 31 serious AEs (15(3.3%) TAF/FTC vs. 14(3.0%) SOC) (p=0.84) (Table 2; Table S7); most were hospitalisations with infection. One child died (TAF/FTC+DTG, from hypotension/toxic shock/severe malnutrition, judged by the investigators as ART-unrelated). Twenty-four (2.6%) children experienced a total of 41 ART-modifying AEs (any grade) (11(2.4%) TAF/FTC vs. 13(2.8%) SOC) (p=0.68), of which 33 were tuberculosis-related protocol-specified modifications (Table 2).

**Figure 3:**
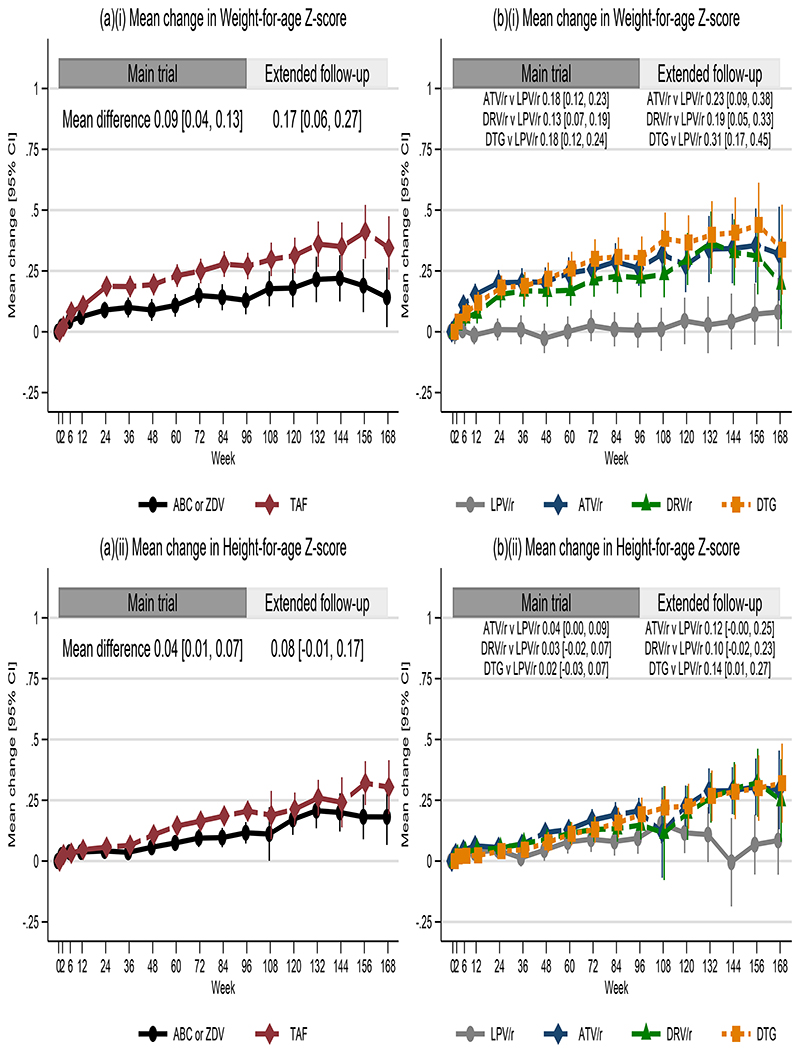
For the backbone (a) and anchor (b) randomisations, change in (i) weight- and (ii) height-for-age Z-scores Extended follow-up mean differences include all of extended follow-up. ABC denotes abacavir, ATV/r denotes ritonavir-boosted atazanavir, DRV/r ritonavir-boosted darunavir, DTG dolutegravir, LPV/r ritonavir-boosted lopinavir, TAF tenofovir alafenamide fumarate & ZDV zidovudine

Over 96 weeks, weight-, height- and BMI-for-age Z-scores increased more with TAF/FTC vs. SOC (mean Z-score difference (averaged over all visits to week 96) +0.09[95% CI +0.04,+0.13], +0.04 [+0.01,+0.07] and +0.10 [+0.04,+0.16], respectively). In extended follow-up, increases were maintained and similar (Figure 3; Figure S6). Comparing TAF/FTC vs. SOC at week-96, corresponding mean weight increase was 7.0 vs. 6.2kg; height increase was 10.2 vs. 9.8cm. There was a small reduction in mean CrCl in both arms at week 96, greater in TAF vs. SOC (mean -16 vs. -11ml/min), which persisted in extended follow-up (Figure S4). Phosphate excretion was similar between arms and no child discontinued TAF for renal dysfunction (Figure S5).

### Anchor randomisation

At randomisation, 910/919(99.0%) initiated their randomised anchor drug (eight with tuberculosis coinfection randomised to ATV/r or DRV/r initiated LPV/r or DTG (protocol-specified modification), one error). Through week-96, children spent 98.6% follow-up on allocated anchor drug (99.1% DTG, 98.5% DRV/r, 98.6% ATV/r, 98.4% LPV/r) and five (0.5%) initiated third-line ART (1 DRV/r, 2 ATV/r, 2 LPV/r). In extended follow-up, children spent 86.2% of time on allocated anchor drug (99.1% DTG, 95.6% DRV/r, 93.7% ATV/r, 54.9% LPV/r) (Figure S1).

At week-96, 92.0% DTG, 88.3% DRV/r, 84.3% ATV/r and 80.7% LPV/r had VL <400 copies/mL (Figure 2). Considering the pre-specified comparisons (Table S5), DTG was superior to LPV/r and ATV/r arms combined (adjusted difference +9.7% [95%CI +4.8%,+14.5%]; p<0.001). DRV/r was not superior to LPV/r and ATV/r combined as the comparison did not meet pre-specified significance (adjusted difference +5.6% [+0.3%,+11.0%]; p=0.04 vs. threshold p=0.03 from multiple comparisons). ATV/r was non-inferior to LPV/r (adjusted difference +3.4% [-3.4%,+10.2%]; p=0.33). Per-protocol analysis was similar (Supplementary appendix: results). For each comparison, there was no evidence of heterogeneity among 11 prespecified sub-groups, including first-line NRTI, randomised backbone, country and baseline VL, apart from marginally greater VL response for DTG vs. LPV/r and ATV/r combined following nevirapine vs efavirenz first-line(Figures S2). In a post-hoc analysis, VL suppression was +4.0% [-1.3%,+9.4%] higher with DTG vs. DRV/r (Table S5). For each comparison, results using <60 and <1000 copies/ml VL thresholds were similar, as was suppression at weeks 48 and 144 (Figure 2; Table S5).

Over 96 weeks, 127/919(13.8%) children experienced grade 3/4 AEs (Table 2; Table S6), most commonly hyperbilirubinemia, predictably almost exclusively ATV/r-associated (Figure S7). Fewer children experienced grade 3/4 AEs with DTG(5.2%) vs. LPV/r(11.5%) (p=0.02); there was no evidence of differences between DRV/r(8.6%) vs. LPV/r(11.5%) (p=0.31). Twenty-nine(3.2%) children experienced serious AEs (6 DTG, 8 DRV/r, 5 ATV/r, 10 LPV/r) (p>0.1) (Table S7). Twenty-four(2.6%) experienced ART-modifying AEs of any grade, with no differences across arms (7 DTG, 5 DRV/r, 5 ATV/r, 7 LPV/r) (p>0.5).

Weight- and BMI-for-age Z-scores increased more with ATV/r, DRV/r and DTG vs. LPV/r (Figure 3; Table S8). There was no evidence that anchor drugs’ effects on weight-for-age Z-scores differed by backbone (Figure S6). Additional secondary outcome analyses (including lipid (Figure S9) and bone health (Figure S10) comparisons) are reported in Supplementary appendix.

### Health economic analysis

TAF/FTC had lower cost than SOC (by $37.68), resulting in a high probability of being cost-saving. DTG was the least costly anchor drug, saving $190.77 compared to ATZ/r; DRV/r was the most expensive (Supplementary appendix).

## Discussion

TAF/FTC provided superior virological suppression vs. ABC/3TC or ZDV/3TC. DTG-based regimens were virologically superior vs. LPV/r and ATV/r arms combined; DRV/r-based regimens achieved higher virological suppression than LPV/r and ATV/r arms combined but could not be declared superior (although significance was close to the multiple-comparison adjusted threshold). LPV/r was associated with the poorest virological outcomes, growth, lipid profiles (Figure S9) and bone health (Figure S10). These comparisons between TAF/FTC (including a new 120/15mg paediatric formulation) and SOC, and the four main currently available second-line anchor drugs for children provide much-needed robust evidence to guide future drug formulation development and paediatric guidelines.

Children did well clinically with infrequent hospitalisation or disease progression and only one death over 96 weeks (due to advanced disease). This is in part attributable to relatively high baseline CD4 counts, supporting the principle of switching to second-line before evidence of significant immune-compromise.

The superior virological suppression of 89.4% at 96 weeks observed with TAF/FTC is comparable to the 93-100% reported in four small single-arm paediatric trials of TAF.^13^ Of note, >85% were virologically suppressed at baseline in these studies, whereas all children in CHAPAS-4 had baseline VL >400 copies/ml. Our results are also similar to the 86-92% virological suppression on TDF or TAF in the adult African NADIA and VISEND second-line trials,^5–7^ and the 84-86% VL suppression at 96 weeks in a pooled analysis of TDF/TAF in 14 adult initial treatment trials.^14^

Weight-, height- and BMI-for-age z-scores all increased more with TAF/FTC, suggesting overall better growth which is potentially a consequence of improved virological suppression. There was no evidence of bone toxicity with TAF, and if anything, greater increases in bone mineral density vs. SOC as assessed by total-body-less-head dual-energy X-ray absorptiometry (irrespective of anchor drug) (Figure S10). These findings, alongside the additional benefits of smaller pill size, once-daily administration, lower cost and lower risk of hypersensitivity, make TAF a valuable second-line option. Although mean CrCl decreased slightly more over 96 weeks with TAF/FTC, values remained within normal limits, with no clinician-assessed associated grade 3/4 adverse events; no child discontinued medication for renal impairment, and there was no evidence of tubulopathy.

The superior virologic suppression with DTG vs. ATV/r and LPV/r combined extends findings from the ODYSSEY trial which showed superiority of DTG vs. SOC for both first- and second-line ART (ODYSSEY second-line SOC being 72% LPV/r, 24% ATV/r, 1% DRV/r).^15^ CHAPAS-4 provides additional evidence through direct randomised comparisons of DTG and DRV/r vs. ATV/r or LPV/r. Given DTG’s cost-effectiveness, small milligram dosing and authorisation for use below 3 years, these results further support DTG as second-line anchor drug of choice in WHO guidelines (when not used first-line).^8^ WHO also recommends DTG combined with optimised NRTI backbone for adults failing NNRTI-based ART,^8^ based in part on superiority of DTG vs. LVP/r in the DAWNING trial,^16^ and non-inferiority of DTG vs. DRV/r (with TDF or ZDV) in the NADIA trial.^6,7^

CHAPAS-4 demonstrated immune reconstitution for all drugs, particularly during 24 weeks after second-line ART initiation (Figure S8). Age-appropriate weight-gain was observed with all anchor drugs except LPV/r, which showed minimal increases in weight-for-age Z-scores in a population with already low baseline scores (Figure 3). A systematic review and meta-analysis evaluating weight-gain among adults reported greater weight-gain among those receiving DTG with TAF compared to other NRTIs,^17^ but we observed no excessive weight-gain with any anchor/backbone combination, including DTG+TAF/FTC. Excess weight-gain in adults has been associated with advanced immunosuppression at ART initiation, high VL, female sex and black race, mostly occurring in the first 2 years of therapy.^18^ This phenomenon has been described as “return to health” where resting energy expenditure returns to normal as HIV viremia and inflammation are controlled.^19^ CHAPAS-4 participants were either normal or underweight at baseline (Table 1), and none had evidence of obesity. Results may therefore not be generalisable to more overweight paediatric populations. As expected, lipid profiles were less favourable for children on LPV/r (Figure S9) and hyperbilirubinemia was predictably seen with ATV/r (Figure S7).

Our findings also show that DRV/r and ATV/r are effective once-daily treatment options which could be considered if DTG cannot be used second-line. Previous small studies have shown ATV/r to be effective in children and potentially a preferred and better tolerated second-line option compared to LPV/r,^20^ as long as hyperbilirubinemia is not associated with discontinuation. LPV/r use in children has considerable challenges of unpalatability and twice-daily dosing. The additional data on poorer growth, abnormal lipid profiles and lower virological suppression in CHAPAS-4 emphasize that LPV/r may be suboptimal.

Our trial strengths include its power to compare both DTG and DRV/r with ATV/r and LPV/r while employing a factorial design to compare TAF-based with SOC backbones. The trial was conducted in three African countries, including three centres outside capital cities, increasing generalisability of results across sub-Saharan Africa where the majority of CLHIV live. Whilst the findings can inform guidelines on second-line regimen after NNRTI-based first-line ART, children currently initiating first-line DTG will also require robust second-line options. A limitation is that CHAPAS-4 does not provide direct evidence to inform anchor/backbone choice in this situation; however, safety and efficacy could be inferred (given lack of evidence of interaction) and they will undoubtedly remain important future options. The relatively high CD4 counts at enrolment may also limit generalisability to severely immunocompromised children. One factor that may have impacted ATV/r and DRV/r efficacy was the lack of co-formulated tablets, resulting in a relatively high pill burden (although a small 25mg ritonavir generic pill was used). Overcoming this barrier through FDC manufacture may further enhance the effectiveness of ritonavir-boosted PIs for children in future. The open-label design of the trial could have potentially introduced bias; however the primary endpoint (VL) was objective. See Table S1 for further review of representativeness/generalisability.

The impact of baseline genotypic NRTI resistance on risk of virological failure, as well as development of acquired resistance mutations during second-line ART, are important considerations for product/formulation prioritisation. Retrospective analyses of resistance results from all children at baseline and those with VL >400 copies/ml at weeks 48 and/or 96 are ongoing.

Overall, CHAPAS-4 results provide efficacy and safety data for TAF/FTC and DTG for paediatric second-line ART. If scaled up, TAF/FTC could also result in cost savings (Supplementary appendix). DRV/r offers several benefits over ATV/r (e.g. higher resistance barrier, ongoing FDC development) but cannot be used under 3 years and is relatively costly so alternative ritonavir-boosted PI/non-INSTI anchor options for young children remain important.^21^ CHAPAS-4 results support further development of child friendly FDCs of TAF/FTC, with or without anchor drugs, and their inclusion on the priority list of the WHO Paediatric Drug Optimization (PADO) program,^22^ which in turn should inform future guidelines and prioritisation of the most effective paediatric drugs and formulations for roll-out in Africa and globally.

Disclosure forms provided by the authors are available with the full text of this article at NEJM.org.

## Supplementary Material

supplement

## Figures and Tables

**Table 1:** Baseline clinical and demographic characteristics

	NRTI backbone randomisation		Anchor drug randomisation				Total N=919
	Standard-of-care N=461	TAF N=458	LPV/r N=227	ATV/r N=231	DRV/r N=232	DTG N=229	
Male	256 (55.5%)	241 (52.6%)	120 (52.9%)	129 (55.8%)	121 (52.2%)	127 (55.5%)	497 (54.1%)
Age (years)	10 (7, 13)	10 (8, 13)	10 (7, 12)	10 (8, 13)	10 (8, 12)	11 (8, 13)	10 (8, 13)
3-4	21 (4.6%)	18 (3.9%)	12 (5.3%)	14 (6.1%)	7 (3.0%)	6 (2.6%)	39 (4.2%)
5-9	178 (38.6%)	180 (39.3%)	95 (41.9%)	83 (35.9%)	96 (41.4%)	84 (36.7%)	358 (39.0%)
10-15	262 (56.8%)	260 (56.8%)	120 (52.9%)	134 (58.0%)	129 (55.6%)	139 (60.7%)	522 (56.8%)
WHO stage							
1	244 (52.9%)	239 (52.2%)	114 (50.2%)	121 (52.4%)	130 (56.0%)	118 (51.5%)	483 (52.6%)
2	140 (30.4%)	154 (33.6%)	79 (34.8%)	74 (32.0%)	65 (28.0%)	76 (33.2%)	294 (32.0%)
3	63 (13.7%)	50 (10.9%)	30 (13.2%)	29 (12.6%)	27 (11.6%)	27 (11.8%)	113 (12.3%)
4	14 (3.0%)	15 (3.3%)	4 (1.8%)	7 (3.0%)	10 (4.3%)	8 (3.5%)	29 (3.2%)
CD4 (cells/mm^3)*	667 (405, 963)	673 (434, 982)	692 (432, 1035)	685 (446, 943)	682 (416, 1000)	625 (349, 891)	669 (413, 971)
CD4%**	27.5 (19.0, 35.4)	28.3 (20.3, 37.0)	28.7 (19.2, 36.0)	28.0 (20.5, 35.2)	28.0 (19.4, 37.1)	27.0 (18.0, 36.0)	28.0 (19.2, 36.0)
VL (copies/ml)	17909 (5417, 58359)	17265 (5764, 50655)	16885 (6333, 59994)	16784 (5070, 56600)	18675 (6673, 49668)	19409 (4992, 57076)	17573 (5549, 55700)
Weight (kg)	26.1 (20.2, 33.5)	25.8 (21.0, 32.8)	25.1 (20.0, 33.4)	25.2 (20.3, 32.1)	26.0 (21.0, 32.3)	27.0 (21.3, 34.0)	25.9 (20.5, 33.1)
Weight-for-age Z-score***	-1.6 (-2.4, -0.9)	-1.6 (-2.4, -0.9)	-1.5 (-2.3, -0.8)	-1.6 (-2.5, -0.9)	-1.7 (-2.4, -0.9)	-1.6 (-2.5, -0.9)	-1.6 (-2.4, -0.9)
Height (cm)	130.9 (118.0, 142.5)	130.1 (120.7, 141.6)	130.0 (118.2, 142.0)	129.5 (119.0, 140.8)	131.6 (118.7, 142.3)	133.0 (120.6, 143.5)	130.5 (119.4, 142.0)
Height-for-age Z-score***	-1.5 (-2.3, -0.9)	-1.6 (-2.4, -0.8)	-1.5 (-2.3, -0.6)	-1.7 (-2.4, -1.0)	-1.6 (-2.3, -0.8)	-1.5 (-2.5, -0.9)	-1.6 (-2.3, -0.8)
BMI (kg/m^2)	15.4 (14.4, 16.5)	15.5 (14.3, 16.8)	15.5 (14.4, 16.8)	15.5 (14.3, 16.7)	15.4 (14.1, 16.5)	15.5 (14.5, 16.8)	15.5 (14.3, 16.7)
BMI-for-age Z-score***	-1.0 (-1.6, -0.4)	-0.9 (-1.8, -0.3)	-0.8 (-1.6, -0.3)	-1.0 (-1.8, -0.3)	-1.0 (-1.7, -0.5)	-1.0 (-1.7, -0.3)	-1.0 (-1.7, -0.4)
Time on first-line ART (years)	5.6 (3.2, 7.8)	5.5 (3.3, 7.7)	5.2 (3.2, 7.5)	5.4 (3.0, 7.6)	6.0 (3.3, 7.8)	5.7 (3.5, 8.1)	5.6 (3.3, 7.8)
First-line NRTI							
Abacavir	244 (52.9%)	246 (53.7%)	121 (53.3%)	124 (53.7%)	123 (53.0%)	122 (53.3%)	490 (53.3%)
Zidovudine	217 (47.1%)	212 (46.3%)	106 (46.7%)	107 (46.3%)	109 (47.0%)	107 (46.7%)	429 (46.7%)
First-line NNRTI							
Efavirenz	247 (53.6%)	267 (58.3%)	131 (57.7%)	128 (55.4%)	124 (53.4%)	131 (57.2%)	514 (55.9%)
Nevirapine	214 (46.4%)	191 (41.7%)	96 (42.3%)	103 (44.6%)	108 (46.6%)	98 (42.8%)	405 (44.1%)
Randomised NRTI backbone							
Standard-of-care	461 (100.0%)	0 (0.0%)	115 (50.7%)	115 (49.8%)	114 (49.1%)	117 (51.1%)	461 (50.2%)
TAF	0 (0.0%)	458 (100.0%)	112 (49.3%)	116 (50.2%)	118 (50.9%)	112 (48.9%)	458 (49.8%)
Randomised anchor drug							
LPV/r	115 (24.9%)	112 (24.5%)	227 (100.0%)	0 (0.0%)	0 (0.0%)	0 (0.0%)	227 (24.7%)
ATV/r	115 (24.9%)	116 (25.3%)	0 (0.0%)	231 (100.0%)	0 (0.0%)	0 (0.0%)	231 (25.1%)
DRV/r	114 (24.7%)	118 (25.8%)	0 (0.0%)	0 (0.0%)	232 (100.0%)	0 (0.0%)	232 (25.2%)
DTG	117 (25.4%)	112 (24.5%)	0 (0.0%)	0 (0.0%)	0 (0.0%)	229 (100.0%)	229 (24.9%)

ART denotes antiretroviral therapy, ATV/r ritonavir-boosted atazanavir, BMI body mass index, DRV/r ritonavir-boosted darunavir, DTG dolutegravir, LPV/r ritonavir-boosted lopinavir, NNRTI non-nucleoside reverse transcriptase inhibitor, NRTI nucleoside/nucleotide reverse transcriptase inhibitor, TAF tenofovir alafenamide fumarate, and VL HIV viral load

Values are n (%) or median (IQR)

* Missing for 13 patients

** Missing for 14 patients

*** Z-scores determined using British 1990 Reference data, which covers the full age range of CHAPAS-4 children

**Table 2:** Grade 3 and 4, serious and ART-modifying adverse events during 96-week follow-up

	Backbone randomisation		Anchor randomisation				Total N=919
	Standard-of-care N=461	TAF N=458	LPV/r N=227	ATV/r N=231	DRV/r N=232	DTG N=229	
**Grade 3/4**	**64 (13.9%) 93**	**63 (13.8%) 83**	**26 (11.5%) 36**	**69 (29.9%) 92**	**20 (8.6%) 28**	**12 (5.2%) 20**	**127 (13.8%) 176**
Raised bilirubin	25 (5.4%) 32	34 (7.4%) 36	1 (0.4%) 1	57 (24.7%) 66	1 (0.4%) 1	0 (0.0%) 0	59 (6.4%) 68
**Serious adverse event**	**14 (3.0%) 14**	**15 (3.3%) 17**	**10 (4.4%) 10**	**5 (2.2%) 6**	**8 (3.4%) 9**	**6 (2.6%) 6**	**29 (3.2%) 31**
Death	0 (0.0%) 0	1 (0.2%) 1*	0 (0.0%) 0	0 (0.0%) 0	0 (0.0%) 0	1 (0.4%) 1*	1 (0.1%) 1
Life threatening	1 (0.2%) 1	1 (0.2%) 2	1 (0.4%) 1	1 (0.4%) 2	0 (0.0%) 0	0 (0.0%) 0	2 (0.2%) 3
Caused or prolonged hospitalisation	13 (2.8%) 13	14 (3.1%) 16	9 (4.0%) 9	5 (2.2%) 6	8 (3.4%) 9	5 (2.2%) 5	27 (2.9%) 29
Other important medical condition	1 (0.2%) 1	1 (0.2%) 1	2 (0.9%) 2	0 (0.0%) 0	0 (0.0%) 0	0 (0.0%) 0	2 (0.2%) 2
**ART-modifying**	**13 (2.8%) 22**	**11 (2.4%) 19**	**7 (3.1%) 11**	**5 (2.2%) 11**	**5 (2.2%) 9**	**7 (3.1%) 10**	**24 (2.6%) 41**
Psychiatric disorder	0 (0.0%) 0	1 (0.2%) 1	0 (0.0%) 0	0 (0.0%) 0	0 (0.0%) 0	1 (0.4%) 1	1 (0.1%) 1
Acute hepatitis	1 (0.2%) 1	0 (0.0%) 0	1 (0.4%) 1	0 (0.0%) 0	0 (0.0%) 0	0 (0.0%) 0	1 (0.1%) 1
Hypersensitivity reaction	2 (0.4%) 4	0 (0.0%) 0	2 (0.9%) 4	0 (0.0%) 0	0 (0.0%) 0	0 (0.0%) 0	2 (0.2%) 4
Tuberculosis	9 (2.0%) 16	9 (2.0%) 17	4 (1.8%) 6	5 (2.2%) 11	4 (1.7%) 8	5 (2.2%) 8	18 (2.0%) 33
Pregnancy	0 (0.0%) 0	1 (0.2%) 1	0 (0.0%) 0	0 (0.0%) 0	0 (0.0%) 0	1 (0.4%) 1	1 (0.1%) 1
Anaemia	1 (0.2%) 1	0 (0.0%) 0	0 (0.0%) 0	0 (0.0%) 0	1 (0.4%) 1	0 (0.0%) 0	1 (0.1%) 1

ART denotes antiretroviral therapy, TAF tenofovir alafenamide fumarate, ATV/r ritonavir-boosted atazanavir, DRV/r ritonavir-boosted darunavir, DTG dolutegravir and LPV/r ritonavir-boosted lopinavir Excluding extended follow-up after 96 weeks

Showing number of patients with one or more event (% of patients) number of events

* Hypotension/shock/toxic shock (secondary: severe malnutrition; candidiasis of oesophagus, trachea, bronchi or lungs)
