# Supplementary material for: Second-Line Antiretroviral Therapy for Children Living with HIV in Africa
Source: N Engl J Med. Author manuscript; Available in PMC 2025 Jun 23. (PMC7617794; doi:10.1056/NEJMoa2404597)
Supplement: supplement [file EMS204159-supplement-supplement.pdf]

Contents

Supplemental methods 3

Supplemental results 6

Supplemental acknowledgements 7

Figure S1: Treatment received over time from randomisation (extended follow-up from week 120-168) 9

Figure S2: Subgroup analyses for the primary endpoint, viral load suppression <400 copies/ml at week 96 11

Figure S3: Change in BMI-for-age during main trial and extended follow-up 19

Figure S4: Change in creatinine clearance over main trial and extended follow up 21

Figure S5: Change in phosphate excretion over 96 weeks (backbone randomisation) 23

Figure S6: Change in weight-for-age by combined backbone and anchor drug 23

Figure S7: Change in bilirubin (anchor randomisation) 25

Figure S8: Change in (i) CD4 and (ii) CD4% during main trial and extended follow-up 26

Figure S9: Change in (i) total, (ii) HDL and (iii) LDL cholesterol and (iv) triglycerides during main trial and extended follow-up 28

Figure S10: Change in (i) Lumbar total and (ii) total body less head (1) bone mineral content, (2) bone mineral density and (3) bone mineral density Z-score 30

Table S1: Representativeness of study participants 32

Table S2: Weight-band based dosing 34

Table S3: Additional baseline clinical and demographic characteristics 36

Table S4: Viral load at weeks 6, 24, 48, 72, 96, 120, 144 and 168 for thresholds of viral load <400, <60 and <1000 copies/ml 37

Table S5: Viral load comparisons at weeks 48, 96 and 144 for thresholds of viral load <400, <60 and <1000 copies/ml 39

Table S6: Grade 3 and 4 adverse events during 96-week follow-up 41

Table S7: Serious Adverse Events (SAEs) during 96-week follow-up 45

Table S8: Weight-for-age at week 96 by weight-for-age at week 0 by anchor drug 47

Table S9: Number of disease progression events of each type (anchor randomisation) 49

Cost-Effectiveness Supplemental Methods 50

Cost-effectiveness Supplemental Results 50

Table CES1a: Costs of NRTI backbone antiretroviral drugs 52

Table CES1b: Costs of antiretroviral anchor drugs 53

Table CES2: Costs of health care resources used 55

Table CES3a. Resource Use by NRTI backbone randomization groups 56

Table CES3b. Resource use by anchor drug randomization groups 57

Table CES4a. Costs and QALYs by NRTI backbone randomization groups 58

Table CES4b. Costs and QALYs by anchor drug randomization groups 58

Table CES5a. Costs Analysis of NRTI Backbone Randomization Groups 59

Table CES5b. Costs Analysis of Different Comparators for Anchor Drug Randomization Groups 60

Table CES6a. Cost-effectiveness Analysis of NRTI Backbone Randomization Groups 61

Table CES6b. Cost-effectiveness Analysis of Different Comparators for Anchor Drug Randomization Groups 62

References 63

Supplemental methods

CHAPAS-4 was designed from a public health perspective. The nucleos(t)ide reverse transcriptase inhibitor (NRTI) backbone and anchor drug randomisations were separate comparisons. No interaction between the NRTI backbone and anchor drug randomisations was anticipated.

For the NRTI backbone randomisation, there was a single primary comparison (tenofovir alafenamide (TAF) vs. standard-of-care (SOC) in terms of HIV vial load (VL) <400 copies/ml at week 96). This comparison was non-inferiority.

For the anchor drug randomisation, there were three primary comparisons (dolutegravir (DTG) vs. ritonavir-boosted atazanavir (ATV/r) and lopinavir (LPV/r) combined, ritonavir-boosted darunavir (DRV/r) vs. ATV/r and LPV/r combined, ATV/r vs. LPV/r). The DTG vs. ATV/r and LPV/r combined and DRV/r vs. ATV/r and LPV/r combined comparisons were superiority. The ATV/r vs. LPV/r comparison was non-inferiority. The comparison of ATV/r vs. LPV/r was designed to demonstrate non-inferiority, since at the time the trial was designed there was little evidence to suggest superiority, particularly given ATV/r was only available as separate pills in children which could affect adherence. Logically therefore, given this non-inferiority hypothesis, the comparison of the drugs where superiority was anticipated (DTG, DRV/r) used this pooled comparator (LPV/r and ATV/r combined) to increase power.

Previous second-line treatment trials in adults have used non-inferiority margins of 10%[1], 12%[2-4] or 15%[5]. The strictest of these previous non-inferiority margins was chosen for the TAF versus SOC comparison (10%) and 12% for the LPV/r versus ATV/r comparison because clinician concerns about toxicity of TAF in children in terms of growing bones are greater than concerns about hyperbilirubinaemia with ATV/r versus hyperlipidaemia/gastrointestinal side-effects with LPV/r, and because ATV/r vs LPV/r are both drugs from the same class.

For both randomisations, the primary endpoint was VL <400 copies/mL at week 96. This was based on a single value because the VL testing was 24-weekly, with additional testing at week 6; some testing was also retrospective. Death counted as failure (VL ≥400 copies/mL). The primary analysis was intent-to-treat (treatment switches disregarded) because CHAPAS-4 was designed from a public health perspective. Children lost to follow-up were excluded from the analysis. There were no other missing week 96 VL values. The intention to treat analysis assumes that any treatment switches that happened during the trial would generalise to those that would happen outside the trial, and effectively compares the “intention to start treatment” with different drug regimens. Counting treatment switches (which are mostly for AEs) as “failures”, as in the FDA snapshot algorithm commonly used in regulatory trials, is a different type of estimand, and fundamentally a composite endpoint of viral failure or treatment switch. As this was designed as a public health trial, the FDA snapshot algorithm was not specified as an analysis or composite endpoint in the protocol or statistical analysis plan.

Division of AIDS table for grading the severity of adverse events (v 2.1 published July 2017)[6] apart from neutrophil gradings, were additionally based on WHO guidelines[7] recognising the lower normal levels in African populations.

Subgroup analyses for the primary outcome were planned by the randomisation stratification factors (site, failing first-line NRTI) together with country, age (3-4, 5-9, 10-15 years), sex, weight-band (14-<20, 20-<25, 25-<35, ≥35 kg), VL at failure (<10000, 10000-99999, ≥100000 copies/mL), and the other factorial randomisation. These eight subgroup analyses were pre-specified in the trial protocol.

An additional three subgroup analyses were planned by failing first line NNRTI, weight-for-age (terciles) and baseline CD4 (terciles) (specified in the statistical analysis plan).

Sub-group analysis used logistic regression adjusting for sub-group (and interactions between sub-group and randomised group), then marginal estimation of risk differences.

Real-time/local VL were done at screening, weeks 48 and 96 following WHO recommendations for annual VL monitoring. Retrospective viral loads were performed using stored plasma at weeks 6, 24 and 72, and at weeks 48 and 96 where a real-time VL was not done. Viral load testing at weeks 24 and 72 could be performed in real-time if part of routine care.

Any observed real-time detectable VL was followed by intensified adherence counselling following the protocol, and repeated 12 weeks later. Local/regional policies for routine resistance testing were followed by individual sites. Local/regional policies for determining third-line regimens were followed alongside guidance provided by the WHO. At the end of the trial, batched genotypic resistance testing was performed retrospectively on stored samples from all patients who had VL >400 copies/ml at week 48 or 96 and this is also being performed on baseline samples.

For time on randomised antiretroviral therapy (ART), the denominator was time in follow-up (i.e. time on randomised ART as a percent of time in follow-up). For extended follow-up, the denominator was time in extended follow-up (which was variable, depending on when each participant joined the trial, given the common end date of extended follow-up).

The effect of the COVID-19 pandemic and change in WHO guidelines
At the end of March 2020, the trial sponsor and the national authorities made the decision to halt recruitment in all sites due to the COVID-19 pandemic. All enrolled participants continued to be supplied with trial medication and were followed up either at the trial clinic, at home or via phone calls, depending on the level of local lockdown. This had cost and resource implications for the sites, including additional transport and personal protective equipment. From June 2020, sites restarted recruitment following review of national guidelines and local mitigation plans. The visit window allowed was increased during periods of lockdown or travel/transport restrictions to allow safety and endpoint tests to be conducted. COVID-19 specific protocol deviations were reviewed regularly, and the impact assessed. A manual of operations as well as COVID-19 risk management plans for each site were developed to guide these processes.

Delays in recruitment arose from this pause in enrolment and later there were additional challenges due to countries' implementation of updated WHO recommendations that dolutegravir (DTG) based regimens be given to children on first and second-line ART. On 26th March 2021, the independent data monitoring committee and trial steering committee agreed to the proposal by the trial management group that the sample size could be reduced to 920 from 1000 whilst retaining statistical power. This was possible due to the very small loss to follow up (0.5%) compared with that in the original protocol (10%). The revised sample size calculation assumed a 2.5% lost to follow-up rate.

Author contributions
Designed the study: DG, ASW, MBD, VM, CMK, VM, AT
Gathered the data: MBD, VM, HAM, CMK, AL, KD, CC, SM, VM, HM, DB, EN, CS, KJL,KN, LM, IY, MK, MN, JL, BN, WN, MM, GM, AG, KZ, RN, KZ, AT, AB
Analyzed the data: AJS, ASW, YZ, SW
Vouches for the data and analysis: AJS, ASW
Wrote the paper: MBD, VM, AB, DMG, AJS, AS, ASW; all authors commented

Decided to publish the paper: all authors

Wrote the first draft: MBD, VM, AJS
Commented extensively on initial drafts: DG, AB, AS, ASW

Supplemental results

Backbone randomisation

Over 96 weeks, there were only nine WHO stage 3/4 events and one child died (TAF/ emtricitabine (FTC), from hypotension/toxic shock secondary to severe malnutrition, judged unrelated to ART) (WHO 3/4 event/death: 5 TAF/FTC vs. 5 SOC). CD4 counts improved in both arms (+103 vs. +67 cells/mm^3 at week 96; mean difference between arms (averaged over all visits to week 96) +24 [95% CI -9,+58]), as did CD4% (+7.3% vs. +7.5%) (+0.4% [-0.4%,+1.1%]). In extended follow-up, there was no evidence of difference between arms in either CD4 or CD4% (Figure S8).

There was no evidence of differences in fasting lipids (total, high-density lipoprotein (HDL), low-density lipoprotein (LDL) cholesterol and triglycerides) between arms over 96 weeks, nor in extended follow-up (Figure S9).

Calcaneal ultrasounds performed on all children showed no evidence of differences between arms over 96 weeks, nor in extended follow-up. Dual-energy X-ray absorptiometry (DEXA) scans performed in 170 children at weeks 0, 48 and 96 showed no evidence of differences between arms in lumbar total bone mineral content (BMC), bone mineral density (BMD) or BMD Z-score (unadjusted for height) and total body less head (TBLH) BMD Z-score. TBLH BMC and BMD increased slightly more with TAF/FTC vs. SOC (Figure S10).

Anchor randomisation

At week-96, for the per-protocol population, 183/218(83.9%) ATV/r vs. 175/215(81.4%) LPV/r had VL <400 copies/mL (adjusted difference 2.1% [-4.8,8.9]; p=0.55).

Over 96 weeks, there were nine WHO stage 3/4 events (5 DTG, 2 DRV/r, 1 ATV/r, 2 LPV/r) and one death in the DTG arm from hypotension/toxic shock secondary to severe malnutrition, judged unrelated to ART (Table S9).

CD4 count improved in all arms (Figure S8) with no evidence of differences between arms over 96-weeks and in extended follow-up.

Over 96 weeks, creatinine clearance decreased more with DTG vs. LPV/r, ATV/r and DRV/r, although differences were small (in the order of 2ml/min) and within the normal range (Figure S4). Total, HDL and LDL cholesterol increased more with LPV/r vs. ATV/r, DRV/r or DTG (Figure S9). Triglycerides increased in all arms up to 48 weeks, more markedly in the LPV/r arm, then decreased through to week 96.

DEXA scans were undertaken at weeks 0, 48 and 96 in a subset of 170 children. At week-96, TBLH BMC and BMD increased more with ATV/r, DRV/r and DTG vs. LPV/r (Figure S10). TBLH BMD Z-scores also decreased most on LPV/r vs. the other three arms; lumbar total BMD Z-score decreased slightly less with DRV/r vs. LPV/r, ATV/r and DTG. There was no evidence of differences in lumbar total BMC and BMD. Of note the majority of children on SOC remained on ABC or ZDV throughout and only 7 (1.5%) children switched to TDF/TAF over 96 weeks.

Supplemental acknowledgements

We also acknowledge the following individuals in the partner institutions

Clinical Trials Unit:

MRC CTU at UCL

Di Gibb, Sarah Walker, Anna Turkova, Clare Shakeshaft, Moira Spyer, Margaret Thomason, Anna Griffiths, Lara Monkiewicz, Sue Massingham, Alex Szubert, Alasdair Bamford, Katja Doerholt, Amanda Bigault, Nimisha Dudakia, Annabelle South, Nadine Van Looy, Carly Au, Hannah Sweeney, Intira Jeannie Collins

Trial Sites:

Joint Clinical Research Centre Lubowa, Uganda: Cissy M. Kityo, Victor Musiime, Eva Natukunda, Esether Nambi, Diana Rutebarika Antonia, Rashida Nazzinda, Imelda Namyalo, Joan Nangiya, Lilian Nabeeta, Aidah Nakalyango, Lilian Kobusingye, Caroline Otike, Winnie Namala, Phionah Ampaire, Ayesiga Edgar, Claire Nasaazi, Milly Ndigendawani, Paul Ociti, Priscilla Kyobutungi, Ritah Mbabazi, Phyllis Mwesigwa Rubondo, Juliet Ankunda, Mariam Naabalamba, Mary Nannungi, Alex Musiime, Faith Mbasani, Babu Enoch Louis, Josephine Namusanje, Denis Odoch, Edward Bagirigomwa, Eddie Rubanga, Disan Mulima, Paul Oronon, Eram David Williams, David Baliruno, Josephine Kobusingye, Agnes Uyungrwoth, Barbara Mukanza, Jimmy Okello, Emily Ninsiima, Lutaro Ezra, Christine Nambi, Nansaigi Mangadalen, Musumba Sharif, Nobert B. Serunjogi, Otim Thomas

Joint Clinical Research Centre Mbarara, Uganda: Abbas Lugemwa, Shafic Makumbi, Sharif Musumba, Edward Mawejje, Ibrahim Yawe, Linda Jovia Kyomuhendo, Mariam Kasozi, Rogers Ankunda, Samson kariisa, Christine Inyakuwa, Emily Ninsiima, Lorna Atwine, Beatrice Tumusiime, John Ahuura, Deogracious Tukwasibwe, Violet Nagasha, Judith Kukundakwe, Mariam Zahara Nakisekka, Ritah Winnie Nambejja, Mercy Tukamushaba, Rubinga Baker, Edridah Keminyeto, Barbara Ainebyoona, Sula Myalo, Juliet Acen, Nicholas Jinta Wangwe, Ian Natuhurira, Gershom Kananura Natukunatsa

University Teaching Hospital, Zambia: Veronica Mulenga, Chishala Chabala, Joyce Chipili Lungu, Monica Kapasa, Khozya Zyambo, Kevin Zimba, Chungu Chalilwe, Dorothy Zangata, Ellen Shingalili, Naomi Mumba, Nayunda Kaonga, Mukumbi Kabesha, Oliver Mwenechanya, Terrence Chipoya, Friday Manakalanga, Stephen Malama, Daniel Chola

Arthur Davison Children's Hospital, Zambia: Bwendo Nduna, Mwate Mwamabazi, Kabwe Banda, Beatrice Kabamba, Muleya Inambao, Pauline Mahy Mukandila, Mwizukanji Nachamba, Stella Himabala, Shadrick Ngosa, Davies Sondashi, Collins Banda, Mark Munyangabe, Grace Mbewe Ngoma, Sarah Chimfwembe, Mercy Lukonde Malasha, Mumba Kajimalwendo, Henry Musukwa, Shadrick Mumba

University of Zimbabwe Clinical Research Centre, Zimbabwe: James Hakim, Mutsa Bwakura-Dangarembizi, Kusum Nathoo, Taneal Kamuzungu, Ennie Chidziva, Joyline Bhiri, Joshua Choga, Hilda Angela Mujuru, Godfrey Musoro, Vivian Mumbiro, Moses Chitsamatanga, Constantine Mutata, Shepherd Mudzingwa, Secrecy Gondo, Columbus Moyo, Ruth Nhema, Kathryn Boyd, Farai Matimba, Vinie Kouamou, Richard Matarise, Zorodzai Tangwena, Taona Mudzviti, Allen Matubu, Alfred Kateta,

Victor Chinembiri, Dorinda Mukura, Joy Chimanzi, Dorothy Murungu, Wendy Mapfumo, Pia Ngwaru, Lynette Chivere, Prosper Dube, Trust Mukanganiki, Sibusisiwe Weza, Tsitsi Gwenzi, Shirley Mutsai, Misheck Phiri, Makhosonke Ndlovu, Tapiwa Gwaze, Stuart Chitongo, Winisayi Njaravani, Sandra Musarurwa, Cleopatra Langa, Sue Tafeni, Wilbert Ishemunyoro, Nathalie Mudzimirema

Mpilo Central Hospital, Zimbabwe: Wedu Ndebele, Mary Nyathi, Grace Siziba, Getrude Tawodzera, Tracey Makuchete, Takudzwa Chidarura, Shingaidzo Murangandi, Lawrence Mafaro, Owen Chivima, Sifiso Dumani, Beaullar Mampondo, Constance Maphosa, Debra Mwale, Rangarirai Dhlamini, Thabani Sibanda, Nobukhosi Madubeko, Silibaziso Nyathi, Zibusiso Matiwaza, Blessing Sanyanga, Prince Ziyera, Gamuchirai Mauro, Titshabona Ncube, Again Gwapedza, Davison Mashoko

Local External Site Monitors

Uganda: Sylvia Nabukenya, Harriet Tibakabikoba, Sarah Nakalanzi, Cynthia Williams

Zimbabwe: Precious Chandiwana, Winnie Gozhora, Benedictor Dube

Zambia: Sylvia Mulambo, Hope Mwanyungwi

Sub-studies

Pharmacokinetic sub-studies – Radboud University Medical Centre: David Burger, Angela Colbers, Hylke Waalewijn, Lisanne Bevers, Shaghayegh Mohsenian-Naghani, Anne Kamphuis

Pharmacokinetic sub-studies – University of Cape Town: Helen McIlleron, Jennifer Norman, Lubbe Wiesner, Roeland Wasmann, Paolo Denti, Lufina Tsirizani Galileya

Toxicity sub-study: Eva Natukunda, Victor Musiime, Phillipa Musoke

Health Economics sub-study – University of York: Paul Revill, Simon Walker, Yingying Zhang

Trial Committees

Independent Trial Steering Committee Members: Adeodata Kekitiinwa, Angela Mushavi, Febby Banda Kawamya, Denis Tindyebwa, Hermione Lyall, Ian Weller

Independent Data Monitoring Committee Members: Tim Peto, Philippa Musoke, Margaret Siwale, Rose Kambarami

Figure S1: Treatment received over time from randomisation (extended follow-up from week 120-168)

(a) Backbone randomisation

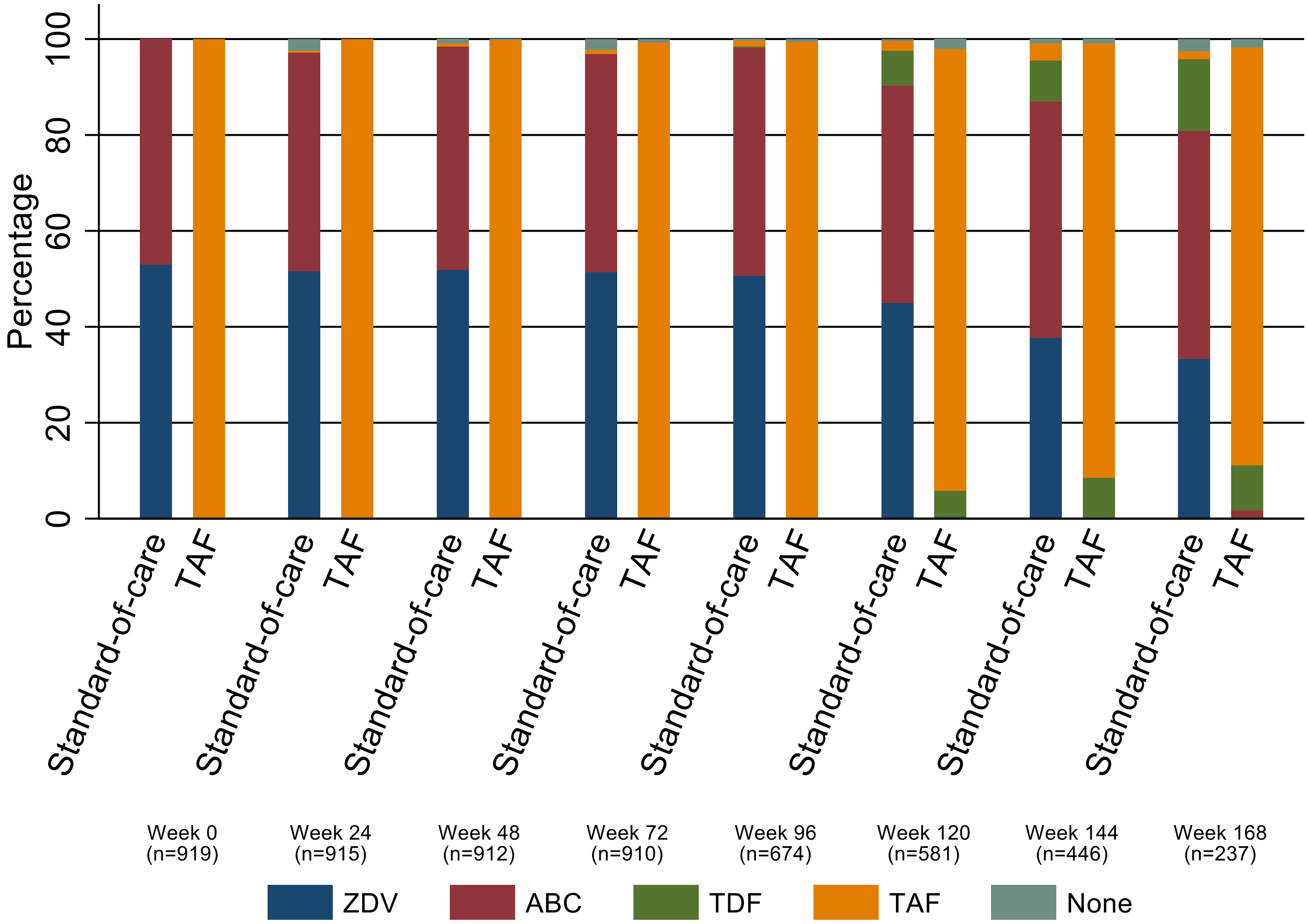

ABC denotes abacavir, TAF tenofovir alafenamide fumarate, TDF tenofovir disoproxil fumarate and ZDV zidovudine

(b) Anchor randomisation

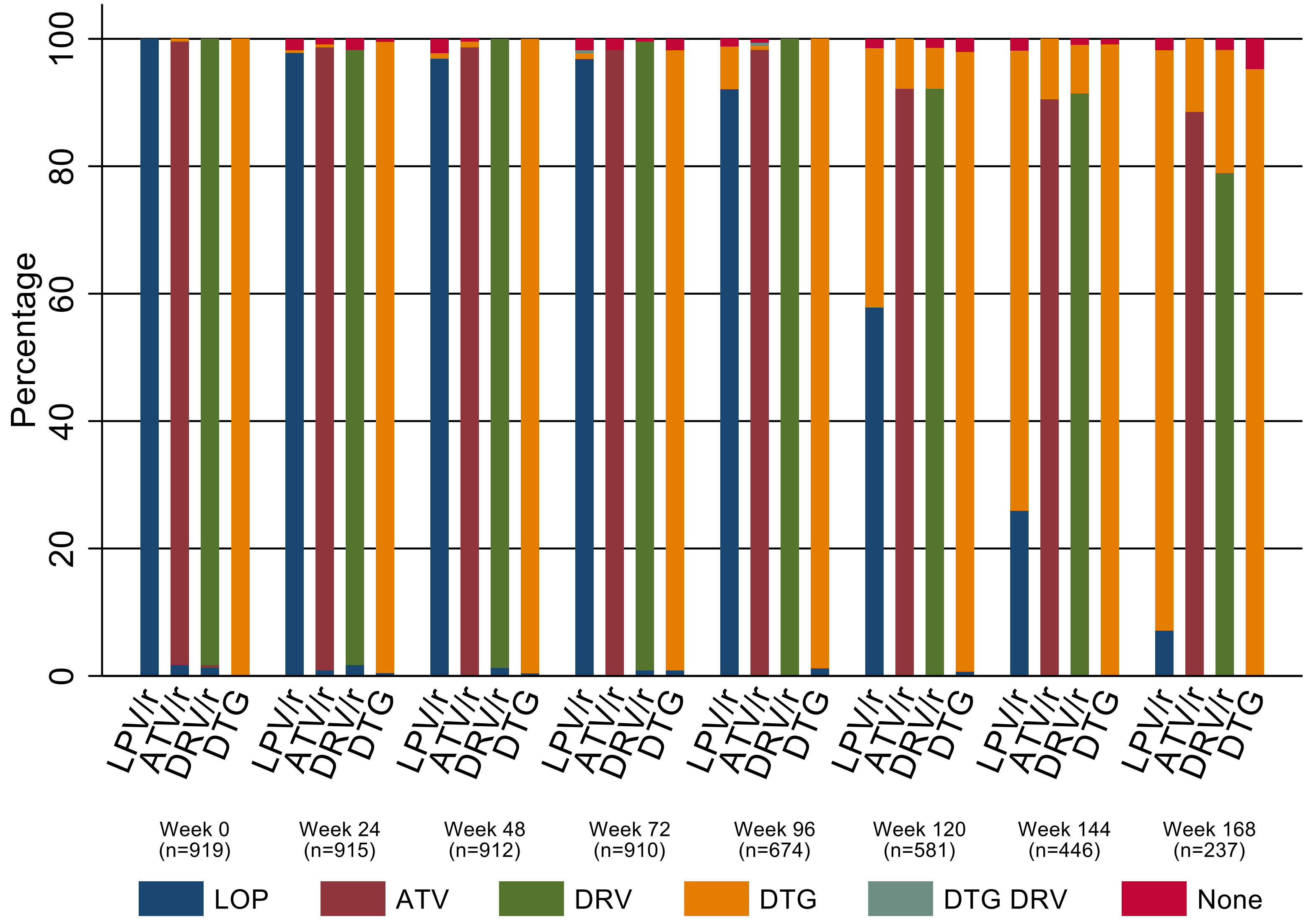

ATV/r denotes ritonavir-boosted atazanavir, DRV/r ritonavir-boosted darunavir, DTG dolutegravir and LPV/r ritonavir-boosted lopinavir

Figure S2: Subgroup analyses for the primary endpoint, viral load suppression <400 copies/ml at week 96

(a) Backbone randomisation

	Standard-of-care	TAF	TAF-Standard-of-care difference (%) [95% CI]
**01 PARTICIPANT HAS BEEN RANDOMISED TO: 3RD DRUG RANDOMISATION***			
LPV/r	83/112(74.1%)	97/111(87.4%)	8.2 [3.0, 13.4]
ATV/r	91/113(80.5%)	102/116(87.9%)	5.6 [-0.6, 11.9]
DRV/r	98/113(86.7%)	105/117(89.7%)	3.1 [-4.8, 11.0]
DTG	106/116(91.4%)	102/110(92.7%)	2.0 [-8.0, 12.0]
**02 SITE**			
Kampala	94/99(94.9%)	96/100(96.0%)	2.5 [-10.5, 15.6]
Mbarara	79/97(81.4%)	90/97(92.8%)	9.1 [3.5, 14.7]
Lusaka	39/57(68.4%)	52/61(85.2%)	8.4 [2.7, 14.2]
Ndola	27/37(73.0%)	28/36(77.8%)	2.7 [-7.5, 12.9]
Harare	90/108(83.3%)	96/109(88.1%)	4.0 [-3.1, 11.1]
Bulawayo	49/56(87.5%)	44/51(86.3%)	-1.2 [-14.6, 12.2]
**03 FAILING FIRST-LINE NRTI**			
ABC	188/239(78.7%)	211/244(86.5%)	5.9 [1.1, 10.7]
ZDV	190/215(88.4%)	195/210(92.9%)	5.5 [-0.5, 11.5]
**04 VL AT TRIAL ENTRY (COPIES/ML)**			
<10000	133/161(82.6%)	143/164(87.2%)	3.8 [-2.2, 9.9]
10000-99999	190/221(86.0%)	208/229(90.8%)	5.1 [-0.6, 10.7]
>=100000	55/72(76.4%)	55/60(91.7%)	9.7 [4.2, 15.2]
**05 COUNTRY**			
Uganda	173/196(88.3%)	186/197(94.4%)	7.6 [1.9, 13.3]
Zambia	66/94(70.2%)	80/97(82.5%)	6.6 [1.1, 12.2]
Zimbabwe	139/164(84.8%)	140/160(87.5%)	2.5 [-4.0, 9.0]

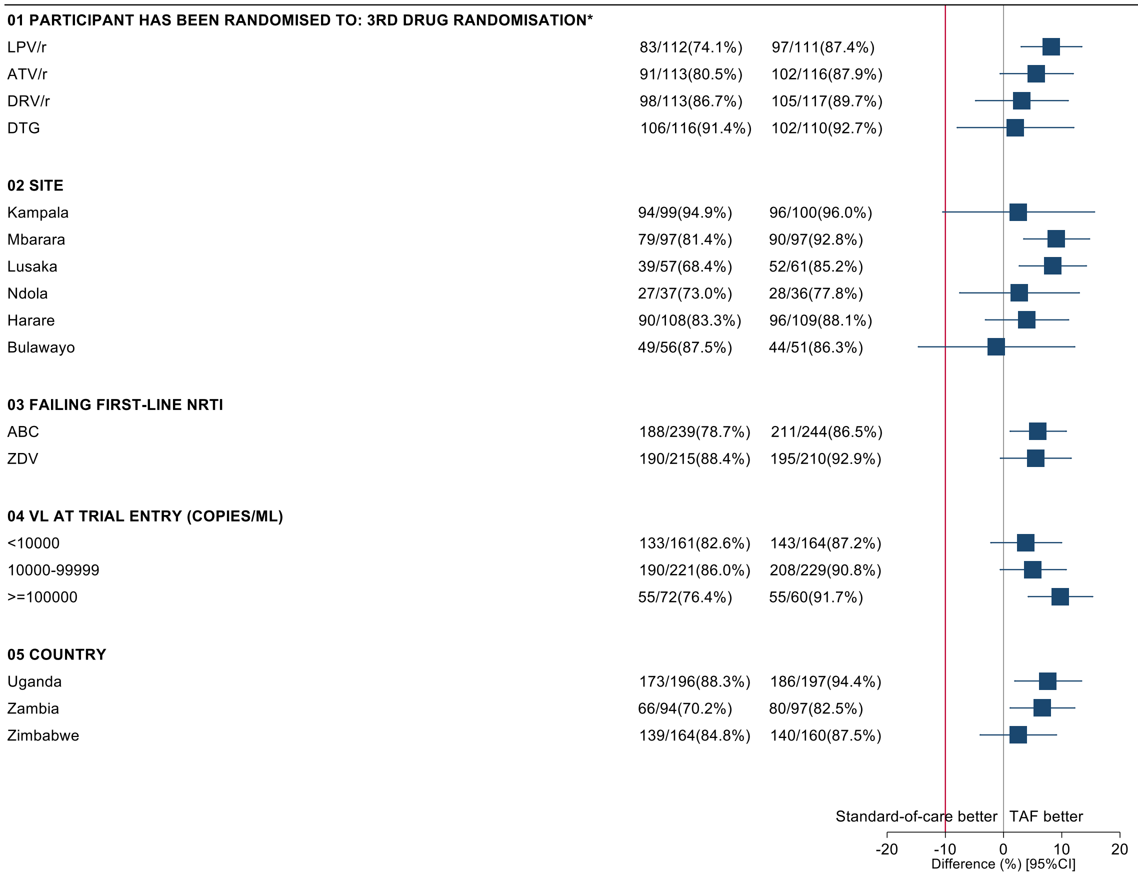

Red line denotes non-inferiority margin (10%)
*Interaction p=0.61

	Standard-of-care	TAF	TAF-Standard-of-care difference (%) [95% CI]
**06 AGE AT LAST BIRTHDAY (YEARS)**			
3-4	18/21(85.7%)	16/17(94.1%)	8.1 [-4.8, 21.0]
5-9	151/172(87.8%)	169/178(94.9%)	8.7 [3.1, 14.2]
10-15	209/261(80.1%)	221/259(85.3%)	4.1 [-0.8, 8.9]
**07 SEX**			
Male	205/250(82.0%)	216/238(90.8%)	7.8 [3.0, 12.7]
Female	173/204(84.8%)	190/216(88.0%)	3.0 [-2.9, 8.9]
**08 WEIGHT (KG)**			
<20	91/104(87.5%)	82/86(95.3%)	8.9 [2.4, 15.4]
20-<25	91/107(85.0%)	105/114(92.1%)	6.8 [0.2, 13.4]
25-<35	125/148(84.5%)	151/171(88.3%)	3.5 [-2.9, 10.0]
>=35	71/95(74.7%)	68/83(81.9%)	4.4 [-2.2, 11.0]
**09 FAILING FIRST LINE NNRTI**			
EFV	191/243(78.6%)	227/264(86.0%)	5.6 [0.8, 10.3]
NVP	187/211(88.6%)	179/190(94.2%)	7.1 [1.2, 12.9]
**10 WEIGHT-FOR-AGE Z-SCORE***			
<=-2.1	119/147(81.0%)	134/154(87.0%)	4.8 [-1.1, 10.7]
>-2.1 to <=-1.13	139/168(82.7%)	125/140(89.3%)	5.6 [-0.3, 11.5]
>-1.13	120/139(86.3%)	147/160(91.9%)	5.9 [-0.6, 12.3]
**11 CD4 AT TRIAL ENTRY (CELLS/MM^3)***			
<=499	120/153(78.4%)	121/144(84.0%)	4.0 [-1.9, 9.8]
>499 - <=852	124/153(81.0%)	140/151(92.7%)	9.8 [4.8, 14.8]
>852	131/145(90.3%)	136/150(90.7%)	0.4 [-8.5, 9.3]

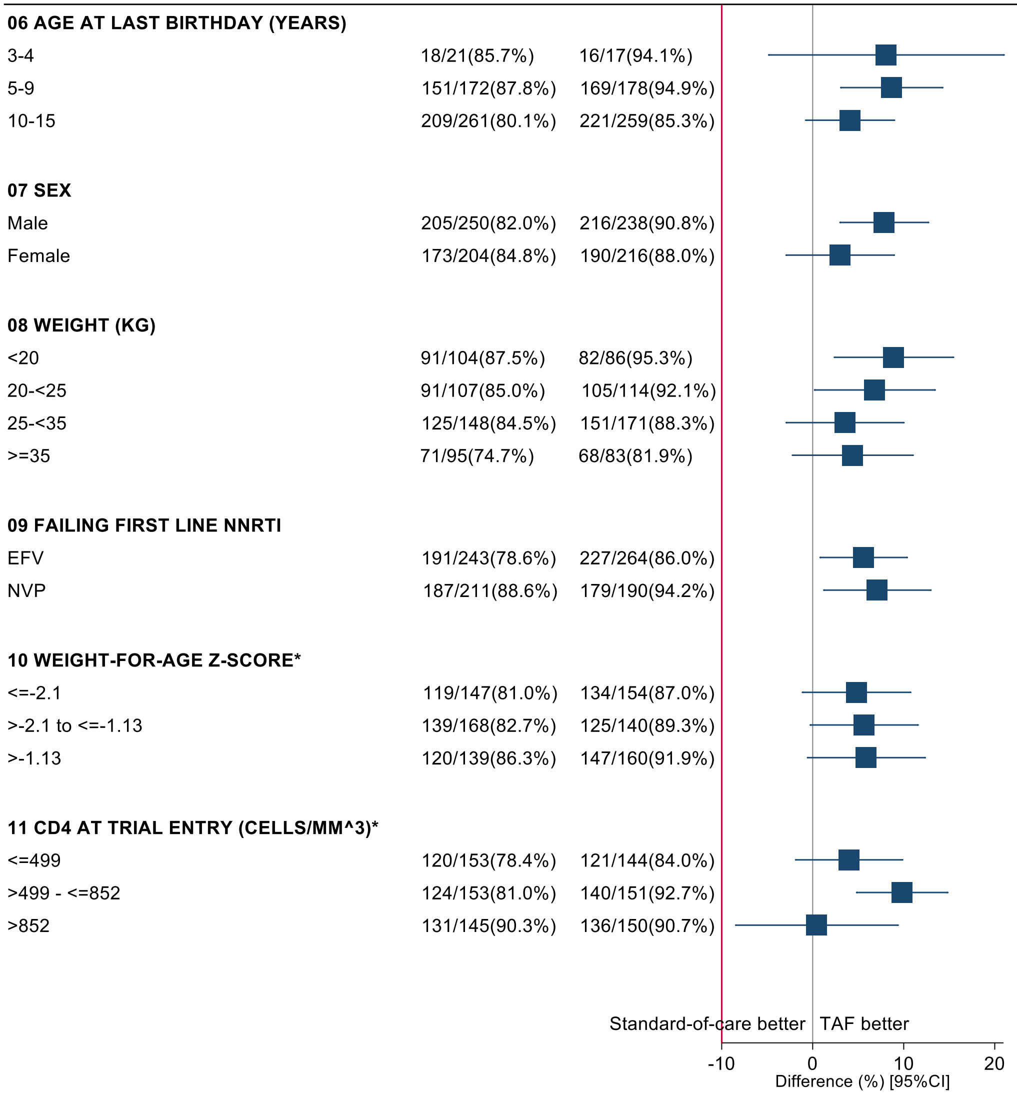

Red line denotes non-inferiority margin (10%)
*Terciles

ABC denotes abacavir, ATV/r ritonavir-boosted atazanavir, DRV/r ritonavir-boosted darunavir, DTG dolutegravir, EFV efavirenz, LPV/r ritonavir-boosted lopinavir, NNRTI non-nucleoside reverse transcriptase inhibitor, NRTI nucleoside/nucleotide reverse transcriptase inhibitor, NVP nevirapine, TAF tenofovir alafenamide fumarate, VL HIV viral load and ZDV zidovudine

(b) Anchor randomisation

(i) ATV/r vs. LPV/r

	LPV/r	ATV/r	ATV/r-LPV/r difference (%) [95% CI]
**01 PARTICIPANT HAS BEEN RANDOMISED TO: NRTI BACKBONE RANDOMISATION***			
Standard-of-care	83/112(74.1%)	91/113(80.5%)	4.9 [-3.1, 12.9]
TAF	97/111(87.4%)	102/116(87.9%)	0.7 [-10.3, 11.7]
**02 SITE**			
Kampala	46/48(95.8%)	47/51(92.2%)	-10.7 [-42.0, 20.6]
Mbarara	40/48(83.3%)	40/49(81.6%)	-1.7 [-16.8, 13.4]
Lusaka	19/29(65.5%)	25/30(83.3%)	10.3 [0.5, 20.1]
Ndola	11/18(61.1%)	15/18(83.3%)	11.5 [1.0, 21.9]
Harare	44/53(83.0%)	42/53(79.2%)	-3.6 [-18.4, 11.2]
Bulawayo	20/27(74.1%)	24/28(85.7%)	8.4 [-3.9, 20.6]
**03 FAILING FIRST-LINE NRTI**			
ABC	88/118(74.6%)	100/123(81.3%)	5.3 [-2.6, 13.1]
ZDV	92/105(87.6%)	93/106(87.7%)	0.2 [-11.4, 11.7]
**04 VL AT TRIAL ENTRY (COPIES/ML)**			
<10000	70/85(82.4%)	70/86(81.4%)	-0.9 [-12.4, 10.5]
10000-99999	84/105(80.0%)	94/107(87.9%)	7.6 [-1.0, 16.2]
>=100000	26/33(78.8%)	29/36(80.6%)	1.5 [-14.3, 17.4]
**05 COUNTRY**			
Uganda	86/96(89.6%)	87/100(87.0%)	-3.6 [-16.9, 9.6]
Zambia	30/47(63.8%)	40/48(83.3%)	11.3 [3.3, 19.3]
Zimbabwe	64/80(80.0%)	66/81(81.5%)	1.3 [-9.2, 11.8]

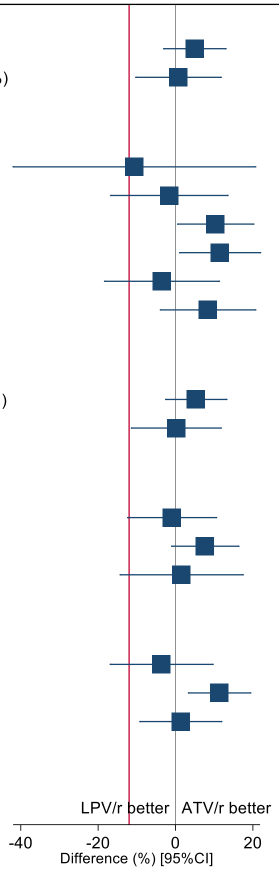

Red line denotes non-inferiority margin (12%)
*Interaction p=0.54

	LPV/r	ATV/r	ATV/r-LPV/r difference (%) [95% CI]
**07 SEX**			
Male	92/116(79.3%)	106/128(82.8%)	3.2 [-5.5, 11.9]
Female	88/107(82.2%)	87/101(86.1%)	4.0 [-5.6, 13.6]
**08 WEIGHT (KG)**			
<20	46/51(90.2%)	41/48(85.4%)	-6.9 [-27.5, 13.6]
20-<25	44/54(81.5%)	57/63(90.5%)	8.9 [-1.3, 19.2]
25-<35	60/71(84.5%)	65/80(81.2%)	-3.3 [-16.2, 9.5]
>=35	30/47(63.8%)	30/38(78.9%)	8.7 [-0.5, 17.8]
**09 FAILING FIRST LINE NNRTI**			
EFV	96/129(74.4%)	104/127(81.9%)	5.9 [-1.7, 13.5]
NVP	84/94(89.4%)	89/102(87.3%)	-3.0 [-16.2, 10.3]
**10 WEIGHT-FOR-AGE Z-SCORE***			
<=-2.1	51/66(77.3%)	58/75(77.3%)	0.0 [-11.1, 11.2]
>-2.1 to <=-1.13	59/75(78.7%)	67/80(83.8%)	4.4 [-5.6, 14.5]
>-1.13	70/82(85.4%)	68/74(91.9%)	8.0 [-2.4, 18.4]
**11 CD4 AT TRIAL ENTRY (CELLS/MM^3)***			
<=499	51/72(70.8%)	54/67(80.6%)	6.9 [-2.1, 15.8]
>499 - <=852	57/70(81.4%)	71/84(84.5%)	3.0 [-8.1, 14.1]
>852	70/79(88.6%)	63/73(86.3%)	-3.1 [-18.1, 11.8]

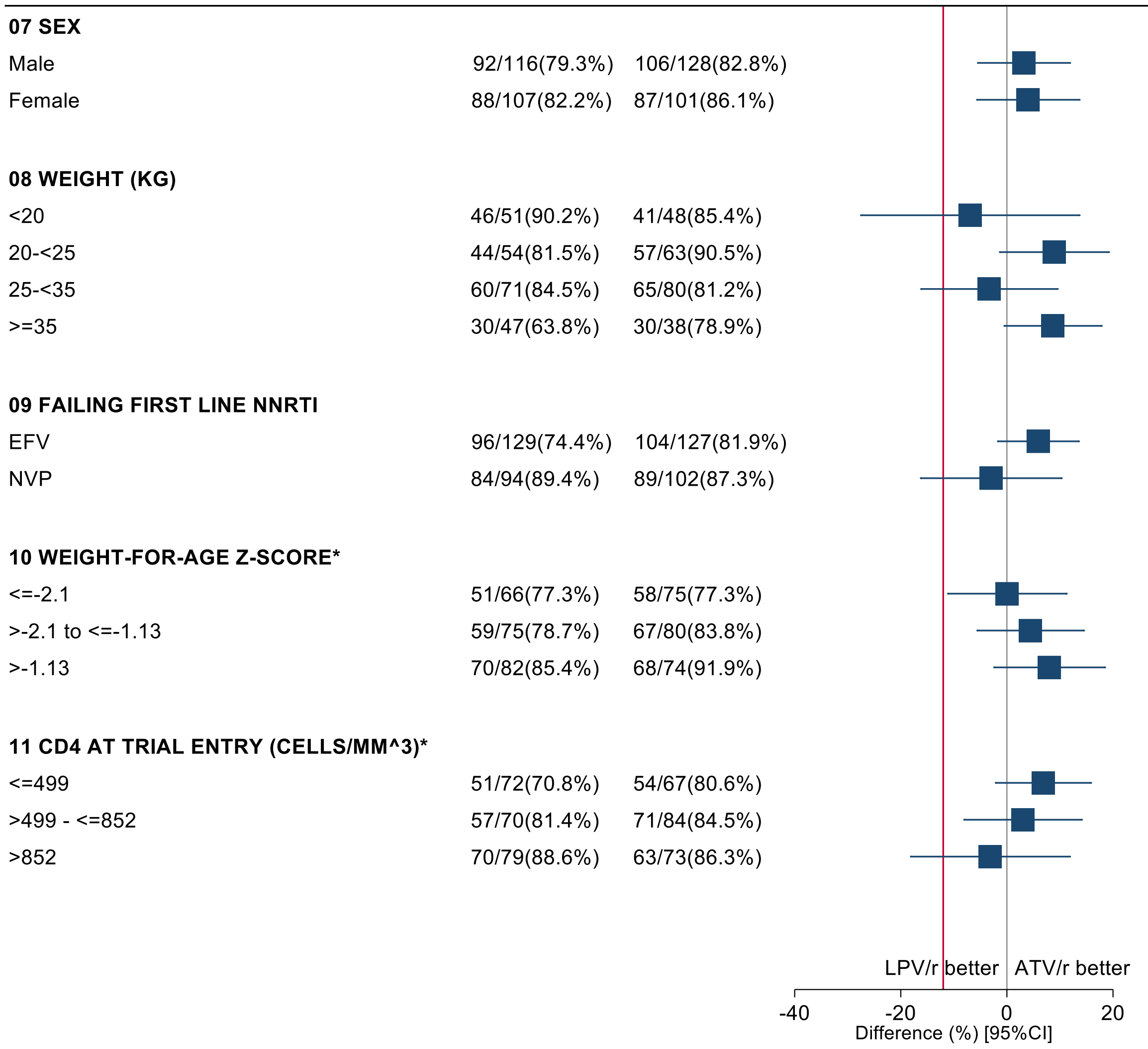

Red line denotes non-inferiority margin (12%)
*Terciles
AGE AT LAST BIRTHDAY (YEARS) (model not fitted due to low numbers) (ATV/r vs. LPV/r):
3-4: 12/14 (85.7%) vs. 11/11 (100.0%); 5-9: 72/82 (87.8%) vs. 81/92 (88.0%); 10-15: 109/133 (82.0%) vs. 88/120 (73.3%)

ABC denotes abacavir, ATV/r ritonavir-boosted atazanavir, EFV efavirenz, LPV/r ritonavir-boosted lopinavir, NNRTI non-nucleoside reverse transcriptase inhibitor, NRTI nucleoside/nucleotide reverse transcriptase inhibitor, NVP nevirapine, TAF tenofovir alafenamide fumarate, VL HIV viral load, ZDV zidovudine

(ii) DRV/r vs. ATV/r and LPV/r combined

	LPV/r / ATV/r	DRV/r	DRV/r-LPV/r / ATV/r difference (%) [95% CI]
**01 PARTICIPANT HAS BEEN RANDOMISED TO: NRTI BACKBONE RANDOMISATION***			
Standard-of-care	174/225(77.3%)	98/113(86.7%)	7.3 [1.2, 13.3]
TAF	199/227(87.7%)	105/117(89.7%)	2.5 [-5.8, 10.9]
**02 SITE**			
Kampala	93/99(93.9%)	50/51(98.0%)	10.2 [-1.5, 21.9]
Mbarara	80/97(82.5%)	42/48(87.5%)	4.5 [-5.6, 14.6]
Lusaka	44/59(74.6%)	24/29(82.8%)	5.4 [-5.1, 15.9]
Ndola	26/36(72.2%)	14/18(77.8%)	3.4 [-10.5, 17.4]
Harare	86/106(81.1%)	48/56(85.7%)	3.9 [-5.6, 13.3]
Bulawayo	44/55(80.0%)	25/28(89.3%)	7.4 [-3.3, 18.2]
**03 FAILING FIRST-LINE NRTI**			
ABC	188/241(78.0%)	105/122(86.1%)	6.4 [0.3, 12.5]
ZDV	185/211(87.7%)	98/108(90.7%)	3.8 [-4.6, 12.2]
**04 VL AT TRIAL ENTRY (COPIES/ML)**			
<10000	140/171(81.9%)	59/71(83.1%)	1.1 [-8.0, 10.2]
10000-99999	178/212(84.0%)	122/132(92.4%)	9.0 [2.7, 15.3]
>=100000	55/69(79.7%)	22/27(81.5%)	1.4 [-12.3, 15.1]
**05 COUNTRY**			
Uganda	173/196(88.3%)	92/99(92.9%)	6.2 [-2.1, 14.4]
Zambia	70/95(73.7%)	38/47(80.9%)	4.7 [-4.0, 13.3]
Zimbabwe	130/161(80.7%)	73/84(86.9%)	5.2 [-2.3, 12.8]

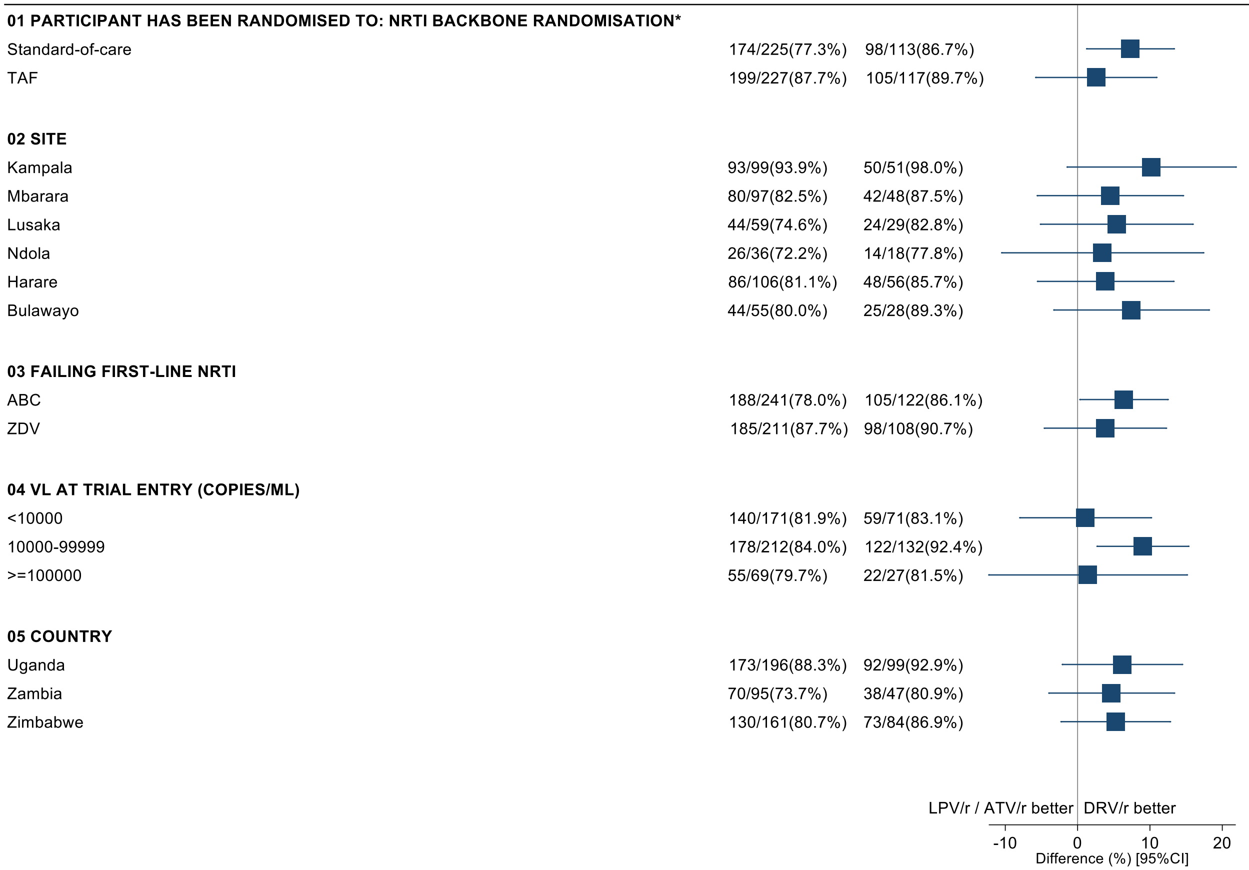

*Interaction p=0.36

	LPV/r / ATV/r	DRV/r	DRV/r-LPV/r / ATV/r difference (%) [95% CI]
**07 SEX**			
Male	198/244(81.1%)	108/119(90.8%)	8.9 [2.8, 14.9]
Female	175/208(84.1%)	95/111(85.6%)	1.4 [-6.6, 9.4]
**08 WEIGHT (KG)**			
<20	87/99(87.9%)	49/50(98.0%)	13.4 [7.0, 19.9]
20-<25	101/117(86.3%)	46/52(88.5%)	2.3 [-9.0, 13.7]
25-<35	125/151(82.8%)	75/86(87.2%)	4.1 [-4.0, 12.2]
>=35	60/85(70.6%)	33/42(78.6%)	4.8 [-3.9, 13.4]
**09 FAILING FIRST LINE NNRTI**			
EFV	200/256(78.1%)	106/122(86.9%)	7.0 [1.0, 13.0]
NVP	173/196(88.3%)	97/108(89.8%)	2.0 [-7.1, 11.0]
**10 WEIGHT-FOR-AGE Z-SCORE***			
<=-2.1	109/141(77.3%)	68/77(88.3%)	8.4 [1.7, 15.0]
>-2.1 to <=-1.13	126/155(81.3%)	74/84(88.1%)	6.0 [-1.5, 13.5]
>-1.13	138/156(88.5%)	61/69(88.4%)	-0.1 [-11.5, 11.4]
**11 CD4 AT TRIAL ENTRY (CELLS/MM^3)***			
<=499	105/139(75.5%)	63/78(80.8%)	3.7 [-3.9, 11.3]
>499 - <=852	128/154(83.1%)	67/73(91.8%)	8.5 [1.0, 16.0]
>852	133/152(87.5%)	71/77(92.2%)	5.9 [-3.3, 15.1]

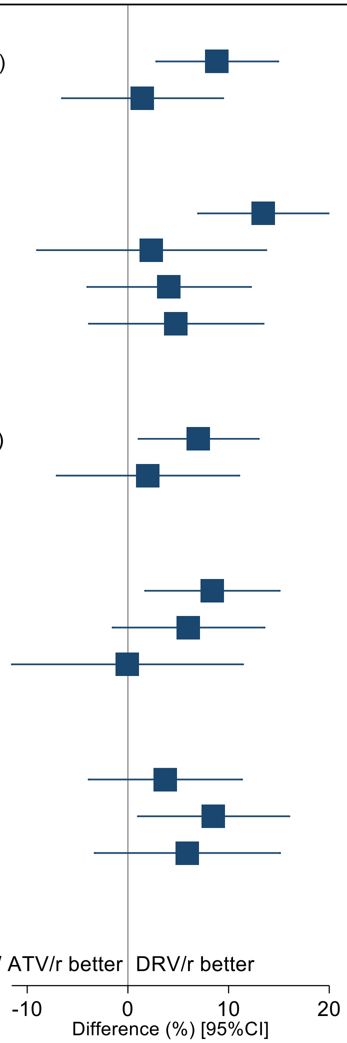

*Terciles
AGE AT LAST BIRTHDAY (YEARS) (model not fitted due to low numbers) (DRV/r vs. LPV/r / ATV/r):
3-4: 7/7 (100.0%) vs. 23/25 (92.0%); 5-9: 88/94 (93.6%) vs. 153/174 (87.9%); 10-15: 108/129 (83.7%) vs. 197/253 (77.9%)

(iii) DTG vs. ATV/r and LPV/r combined

	LPV/r / ATV/r	DTG	DTG-LPV/r / ATV/r difference (%) [95% CI]
**01 PARTICIPANT HAS BEEN RANDOMISED TO: NRTI BACKBONE RANDOMISATION***			
Standard-of-care	174/225(77.3%)	106/116(91.4%)	10.5 [5.4, 15.6]
TAF	199/227(87.7%)	102/110(92.7%)	6.0 [-1.2, 13.2]
**03 FAILING FIRST-LINE NRTI**			
ABC	188/241(78.0%)	106/120(88.3%)	7.7 [2.2, 13.2]
ZDV	185/211(87.7%)	102/106(96.2%)	10.7 [4.8, 16.6]
**04 VL AT TRIAL ENTRY (COPIES/ML)**			
<10000	140/171(81.9%)	77/83(92.8%)	9.4 [3.4, 15.4]
10000-99999	178/212(84.0%)	98/106(92.5%)	8.2 [2.1, 14.3]
>=100000	55/69(79.7%)	33/36(91.7%)	8.9 [1.1, 16.7]
**05 COUNTRY**			
Uganda	173/196(88.3%)	94/98(95.9%)	9.8 [3.3, 16.2]
Zambia	70/95(73.7%)	38/49(77.6%)	2.3 [-6.1, 10.8]
Zimbabwe	130/161(80.7%)	76/79(96.2%)	12.9 [8.1, 17.7]

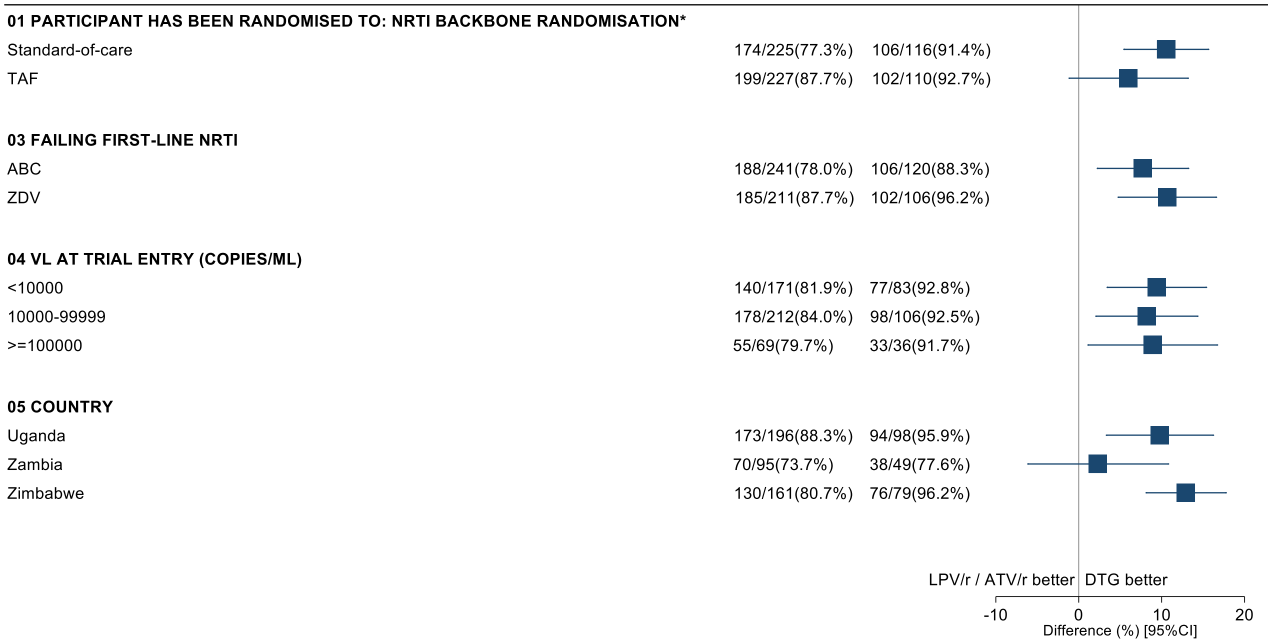

*Interaction p=0.33
SITE (model not fitted due to low numbers) (DTG vs. LPV/r / ATV/r):
Kampala: 47/49 (95.9%) vs. 93/99 (93.9%); Mbarara: 47/49 (95.9%) vs. 80/97 (82.5%); Lusaka: 23/30 (76.7%) vs. 44/59 (74.6%); Ndola: 15/19 (78.9%) vs. 26/36 (72.2%); Harare 52/55 (94.5%) vs. 86/106 (81.1%); Bulawayo: 24/24 (100.0%) vs. 44/55 (80.0%)

ABC denotes abacavir, ATV/r ritonavir-boosted atazanavir, DRV/r ritonavir-boosted darunavir, EFV efavirenz, LPV/r ritonavir-boosted lopinavir, NNRTI non-nucleoside reverse transcriptase inhibitor, NRTI nucleoside/nucleotide reverse transcriptase inhibitor, NVP nevirapine, TAF tenofovir alafenamide fumarate, VL HIV viral load, ZDV zidovudine

	LPV/r / ATV/r	DTG	DTG-LPV/r / ATV/r difference (%) [95% CI]
**07 SEX**			
Male	198/244(81.1%)	115/125(92.0%)	9.5 [4.0, 14.9]
Female	175/208(84.1%)	93/101(92.1%)	7.7 [1.3, 14.1]
**08 WEIGHT (KG)**			
<20	87/99(87.9%)	37/41(90.2%)	2.7 [-9.5, 14.8]
20-<25	101/117(86.3%)	49/52(94.2%)	8.4 [0.3, 16.5]
25-<35	125/151(82.8%)	76/82(92.7%)	8.9 [2.5, 15.2]
>=35	60/85(70.6%)	46/51(90.2%)	10.9 [5.5, 16.4]
**09 FAILING FIRST LINE NNRTI**			
EFV	200/256(78.1%)	112/129(86.8%)	6.5 [0.9, 12.0]
NVP	173/196(88.3%)	96/97(99.0%)	14.4 [10.1, 18.6]
**10 WEIGHT-FOR-AGE Z-SCORE***			
<=-2.1	109/141(77.3%)	76/83(91.6%)	10.3 [4.8, 15.8]
>-2.1 to <=-1.13	126/155(81.3%)	64/69(92.8%)	9.5 [3.4, 15.7]
>-1.13	138/156(88.5%)	68/74(91.9%)	4.2 [-5.0, 13.4]
**11 CD4 AT TRIAL ENTRY (CELLS/MM^3)***			
<=499	105/139(75.5%)	73/80(91.2%)	10.8 [5.4, 16.2]
>499 - <=852	128/154(83.1%)	69/77(89.6%)	5.8 [-1.7, 13.3]
>852	133/152(87.5%)	63/66(95.5%)	9.5 [2.2, 16.9]

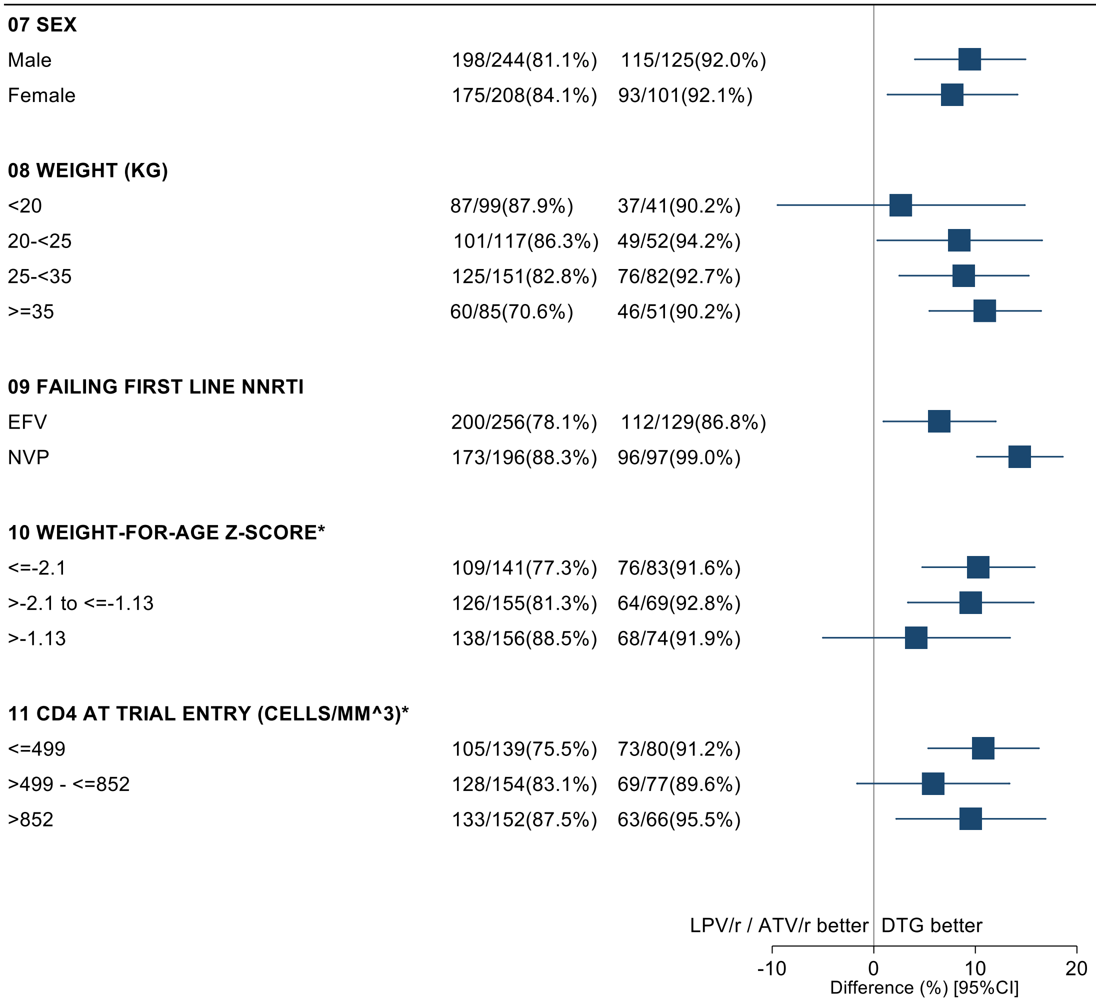

*Terciles
AGE AT LAST BIRTHDAY (YEARS) (model not fitted due to low numbers) (DRV/r vs. LPV/r / ATV/r):
3-4: 7/7 (100.0%) vs. 23/25 (92.0%); 5-9: 88/94 (93.6%) vs. 153/174 (87.9%); 10-15: 108/129 (83.7%) vs. 197/253 (77.9%)

ABC denotes abacavir, ATV/r ritonavir-boosted atazanavir, DTG dolutegravir, EFV efavirenz, LPV/r ritonavir-boosted lopinavir, NNRTI non-nucleoside reverse transcriptase inhibitor, NRTI nucleoside/nucleotide reverse transcriptase inhibitor, NVP nevirapine, TAF tenofovir alafenamide fumarate, VL HIV viral load, ZDV zidovudine

Figure S3: Change in BMI-for-age during main trial and extended follow-up

(a) Backbone randomisation

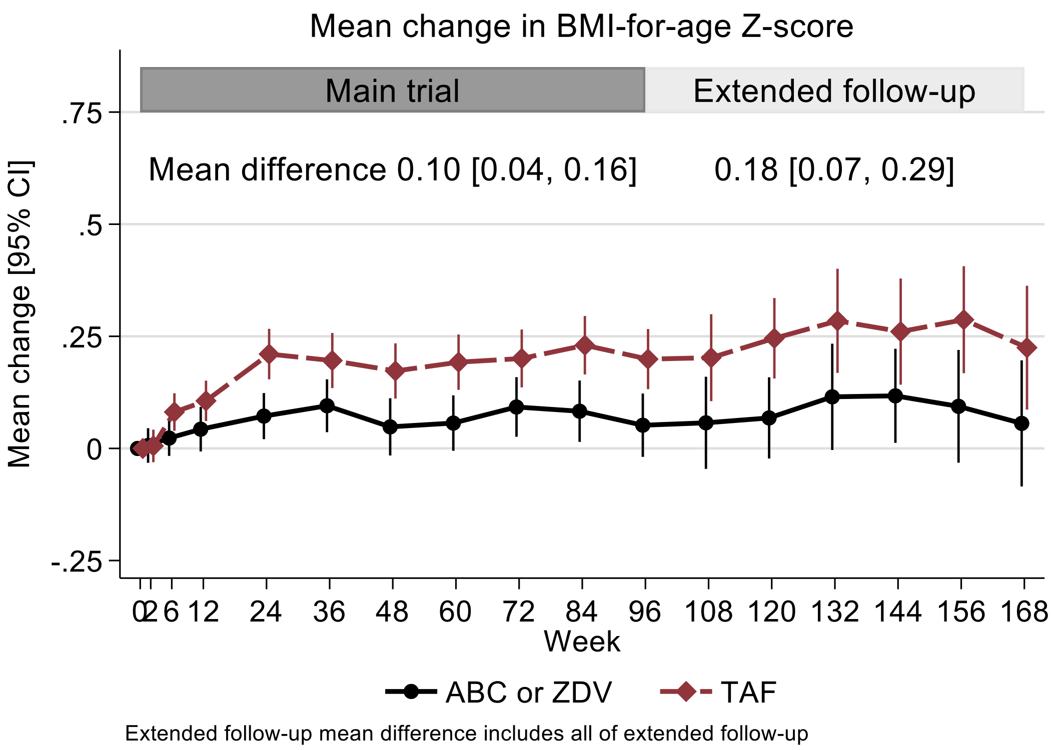

ABC denotes abacavir, BMI body mass index, TAF tenofovir alafenamide fumarate and ZDV zidovudine

(b) Anchor randomisation

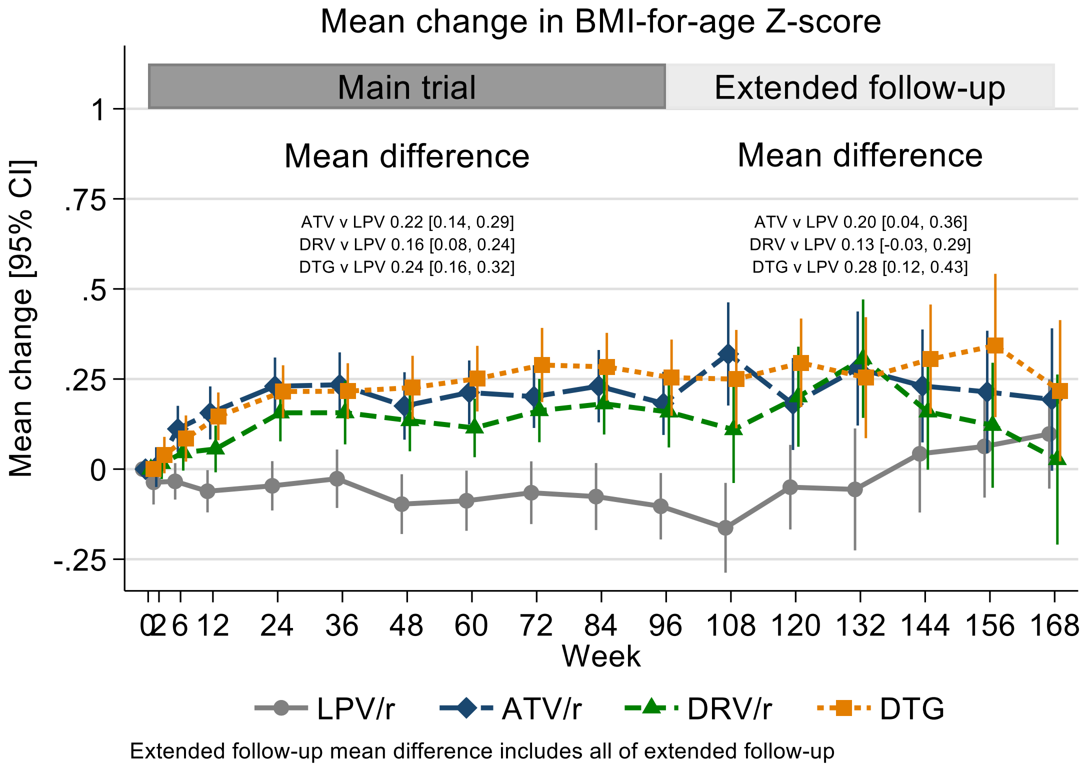

ATV/r denotes ritonavir-boosted atazanavir, BMI body mass index, DRV/r ritonavir-boosted darunavir, DTG dolutegravir and LPV/r ritonavir-boosted lopinavir

Figure S4: Change in creatinine clearance over main trial and extended follow up

(a) Backbone randomisation

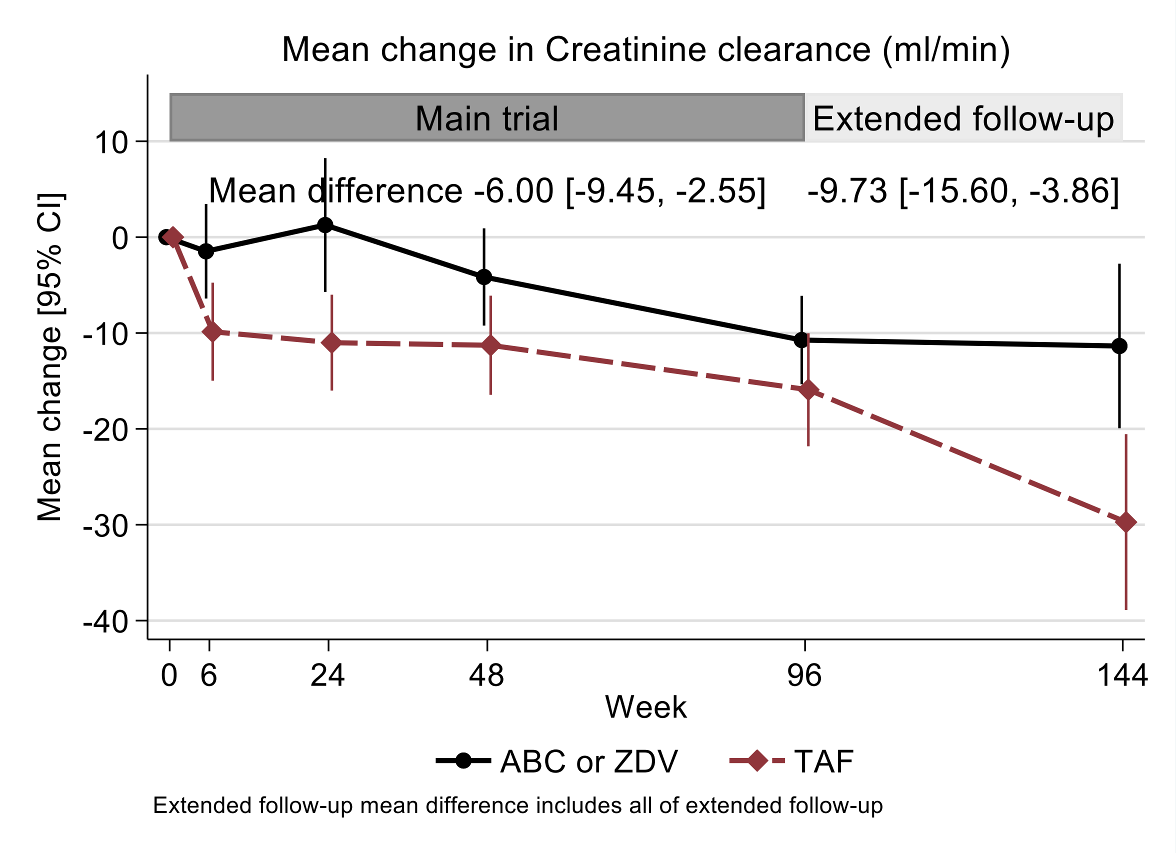

ABC denotes abacavir, TAF tenofovir alafenamide fumarate and ZDV zidovudine

(b) Anchor randomisation

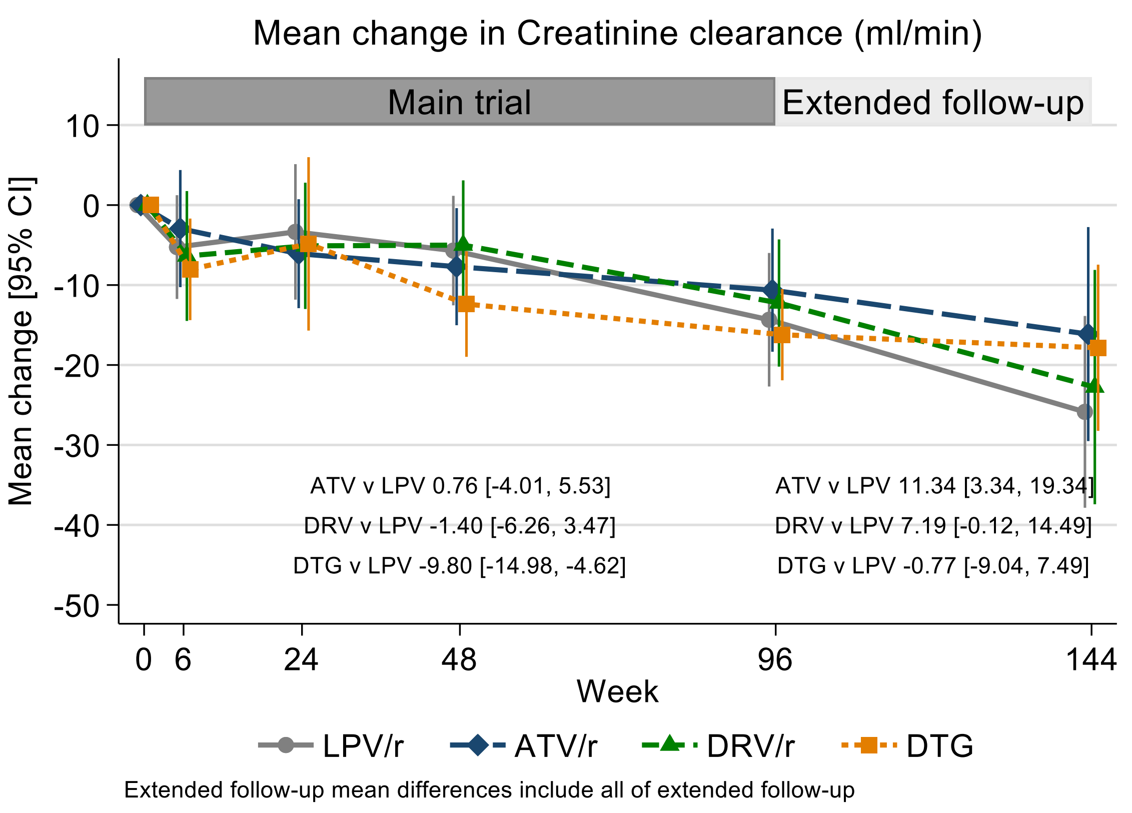

ATV/r denotes ritonavir-boosted atazanavir, DRV/r ritonavir-boosted darunavir, DTG dolutegravir and LPV/r ritonavir-boosted lopinavir

Figure S5: Change in phosphate excretion over 96 weeks (backbone randomisation)

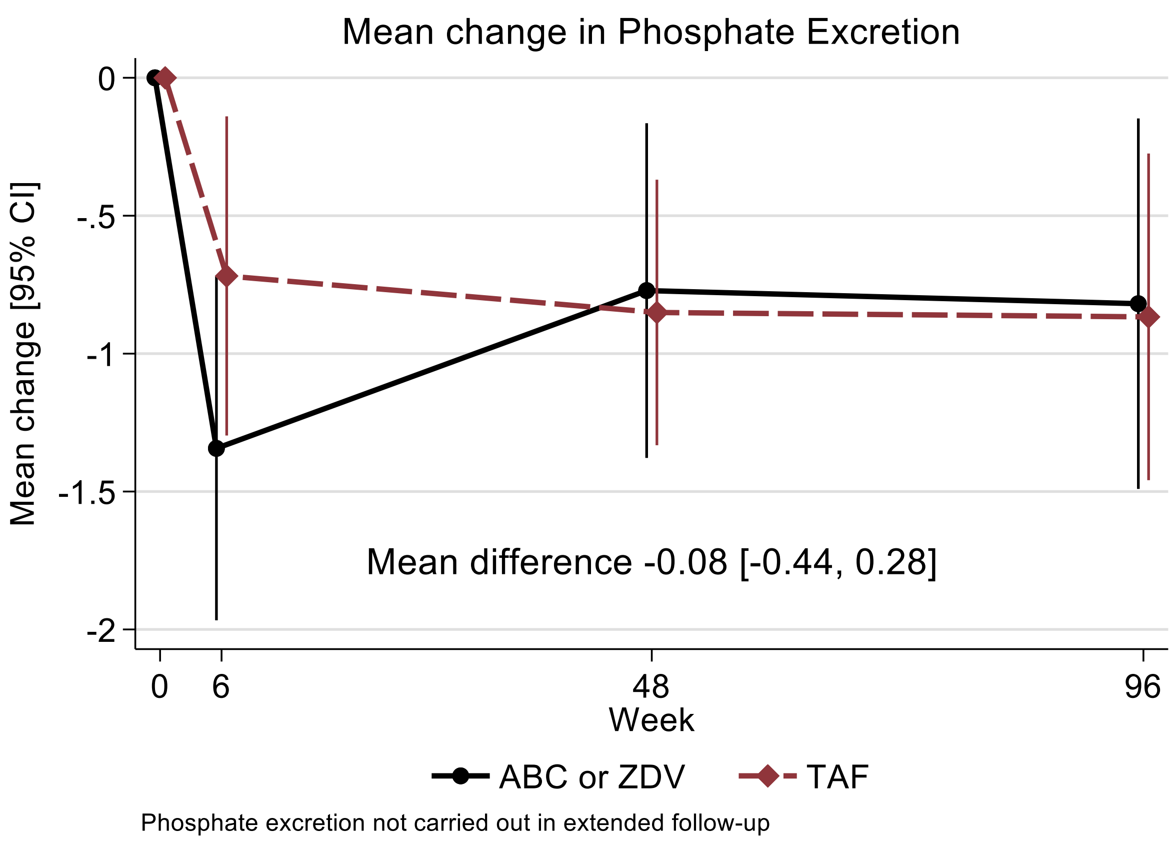

ABC denotes abacavir, TAF tenofovir alafenamide fumarate and ZDV zidovudine

Figure S6: Change in weight-for-age by combined backbone and anchor drug

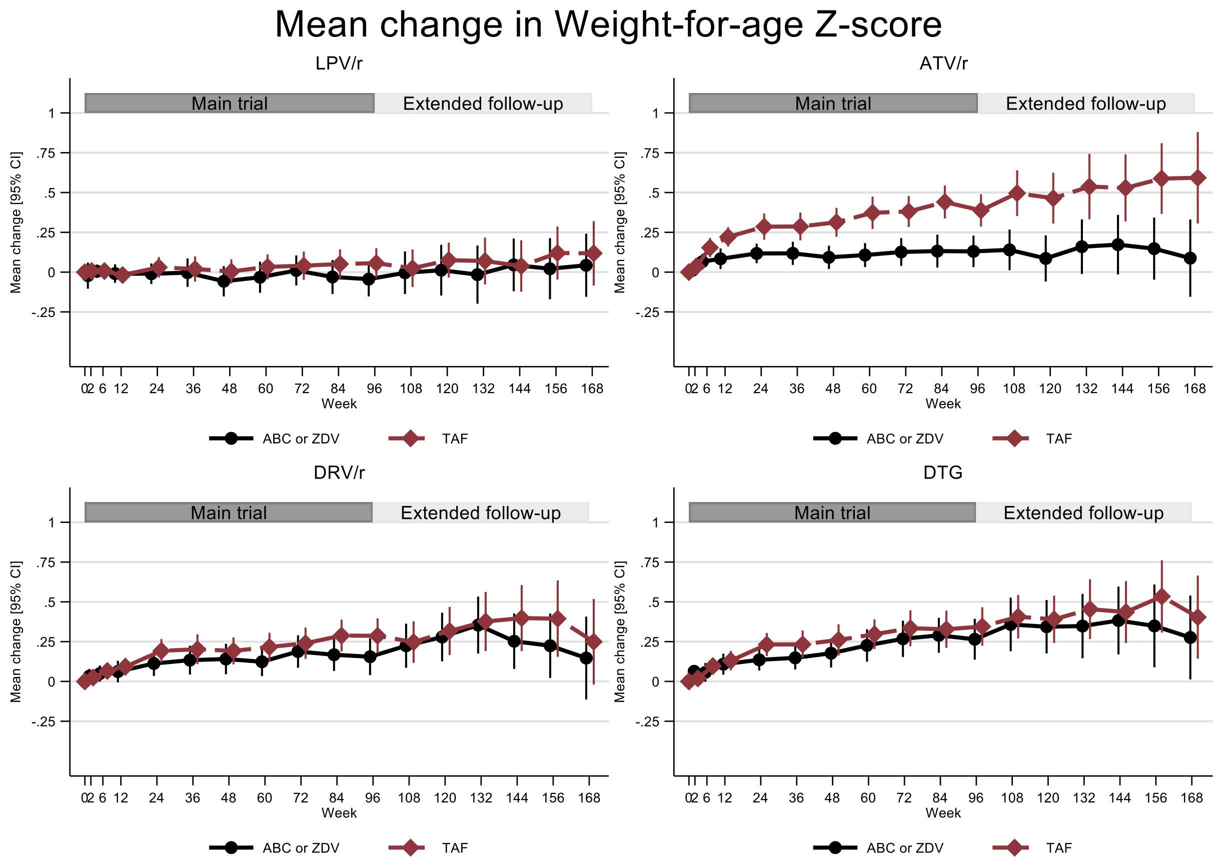

ABC denotes abacavir, ATV/r ritonavir-boosted atazanavir, DRV/r ritonavir-boosted darunavir, DTG dolutegravir, LPV/r ritonavir-boosted lopinavir, TAF tenofovir alafenamide fumarate and ZDV zidovudine

Figure S7: Change in bilirubin (anchor randomisation)

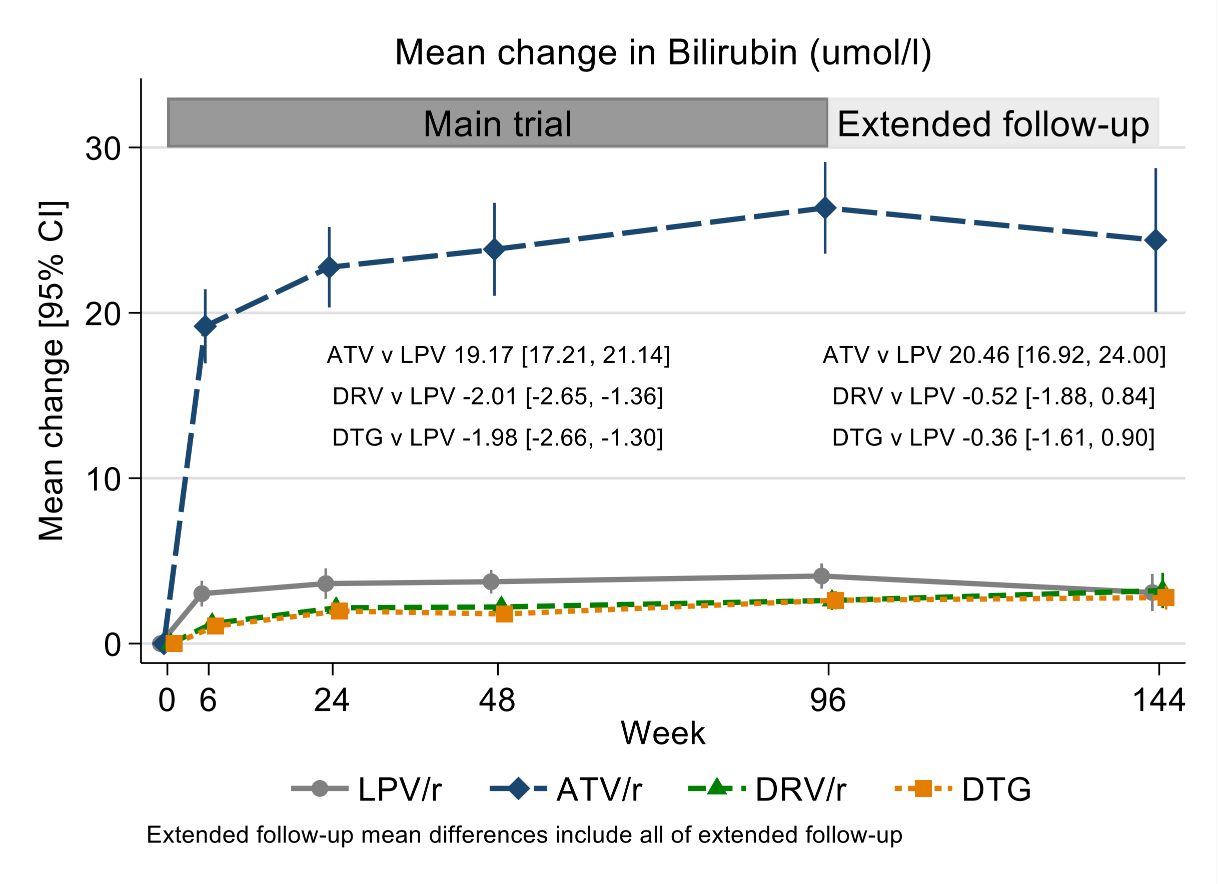

ATV/r denotes ritonavir-boosted atazanavir, DRV/r ritonavir-boosted darunavir, DTG dolutegravir and LPV/r ritonavir-boosted lopinavir

Figure S8: Change in (i) CD4 and (ii) CD4% during main trial and extended follow-up

(a) Backbone randomisation

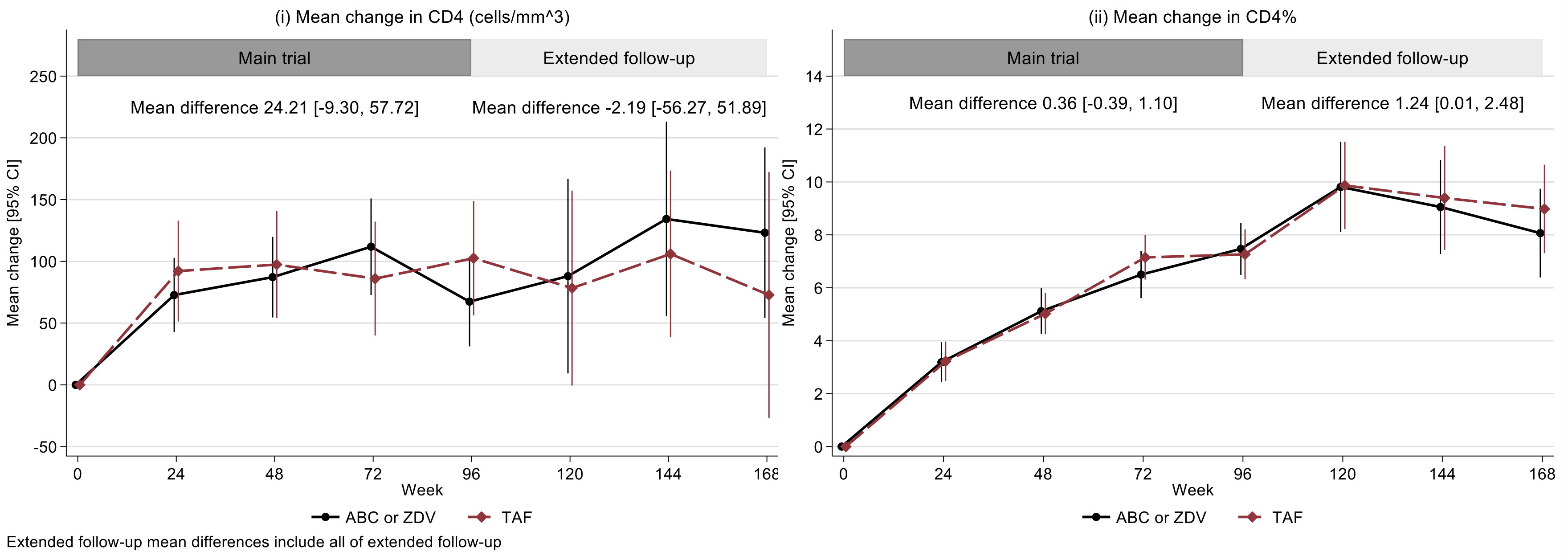

ABC denotes abacavir, TAF tenofovir alafenamide fumarate and ZDV zidovudine

(b) Anchor randomisation

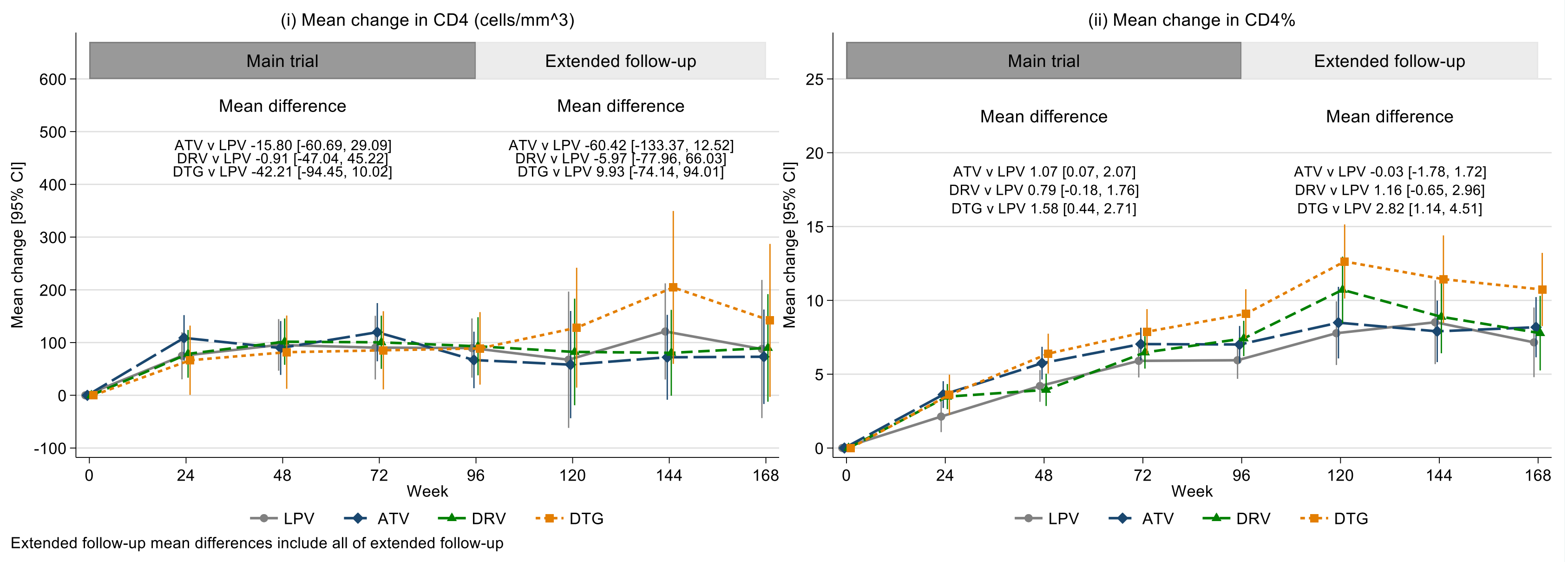

ATV denotes ritonavir-boosted atazanavir, DRV ritonavir-boosted darunavir, DTG dolutegravir and LPV ritonavir-boosted lopinavir

Figure S9: Change in (i) total, (ii) HDL and (iii) LDL cholesterol and (iv) triglycerides during main trial and extended follow-up

(a) Backbone randomisation

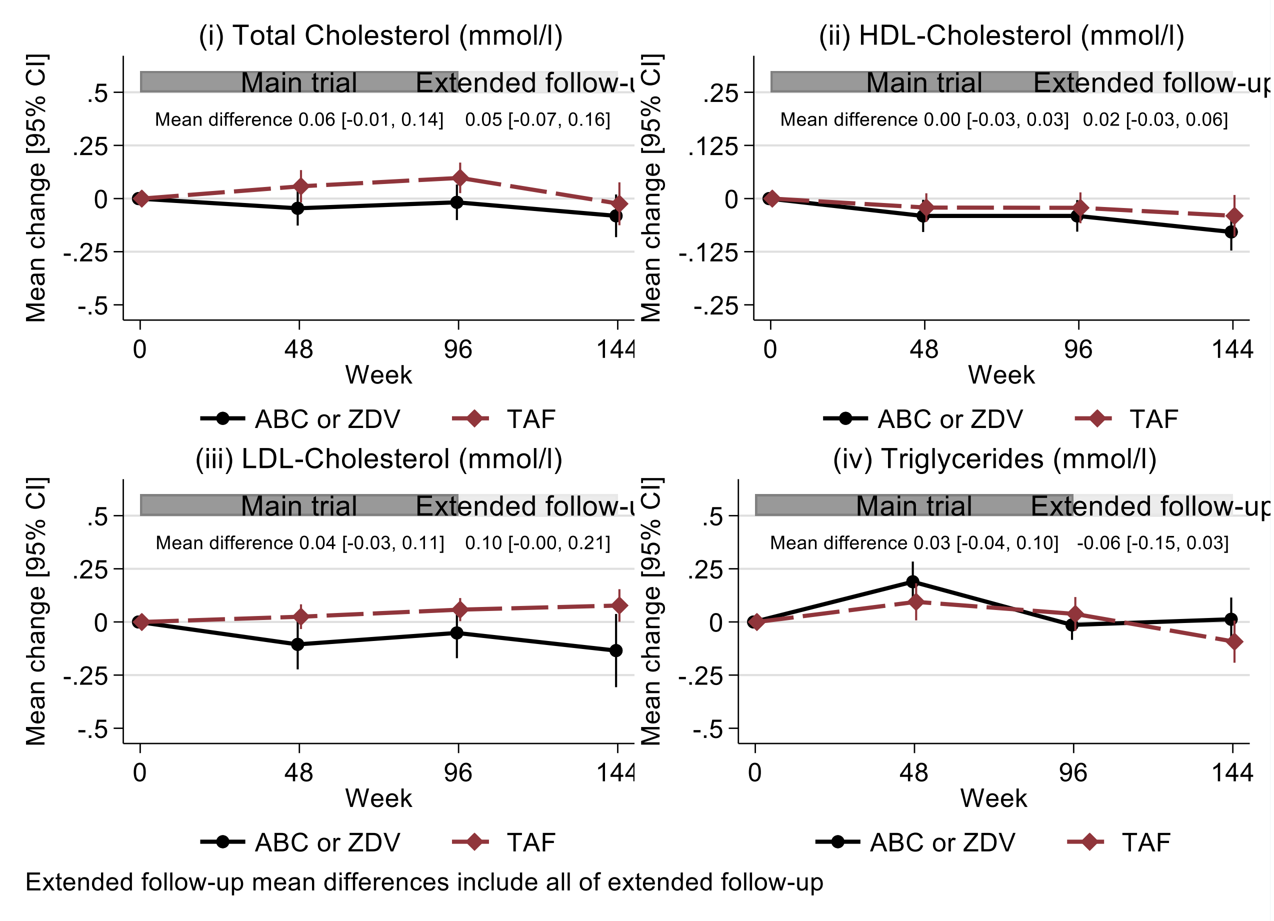

ABC denotes abacavir, HDL high-density lipoprotein, LDL low-density lipoprotein, TAF tenofovir alafenamide fumarate and ZDV zidovudine

(b) Anchor randomisation

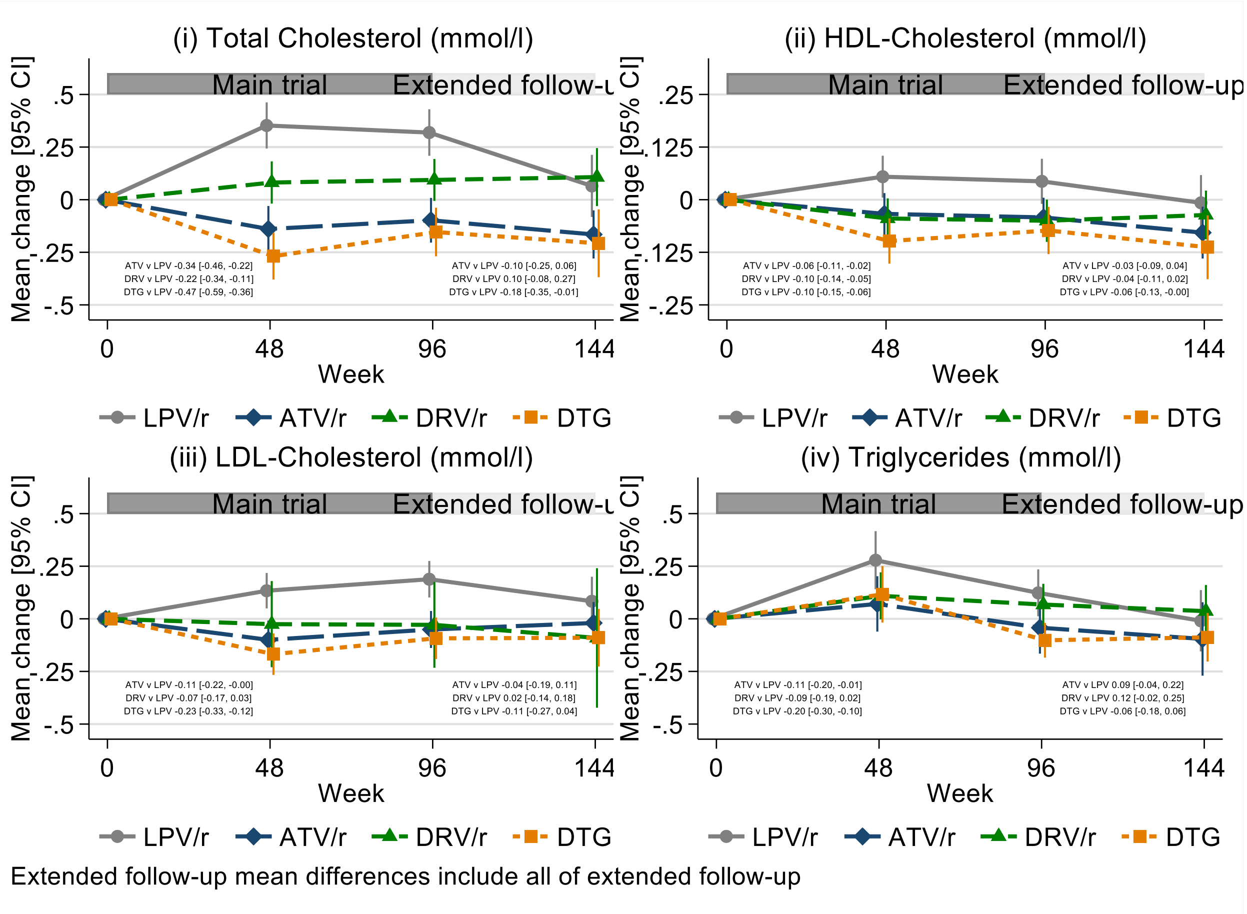

ATV/r denotes ritonavir-boosted atazanavir, DRV/r ritonavir-boosted darunavir, DTG dolutegravir, HDL high-density lipoprotein, LDL low-density lipoprotein and LPV/r ritonavir-boosted lopinavir

Figure S10: Change in (i) Lumbar total and (ii) total body less head (1) bone mineral content, (2) bone mineral density and (3) bone mineral density Z-score

(a) Backbone randomisation

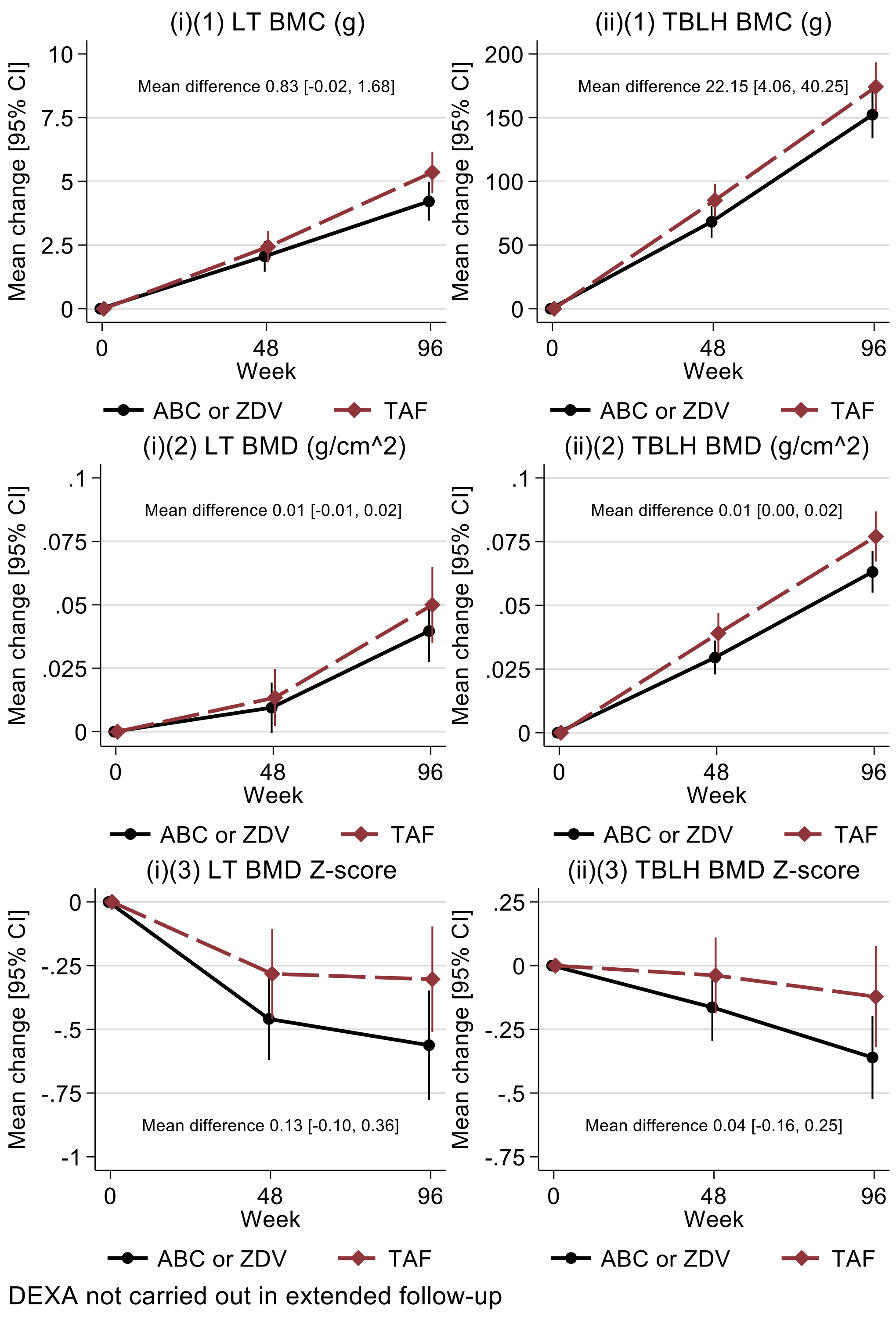

ABC denotes abacavir, BMC bone mineral content, BMD bone mineral density, DEXA dual energy x-ray absorptiometry, LT lumbar total, TAF tenofovir alafenamide fumarate, TBLH total body less head and ZDV zidovudine

(b) Anchor randomisation

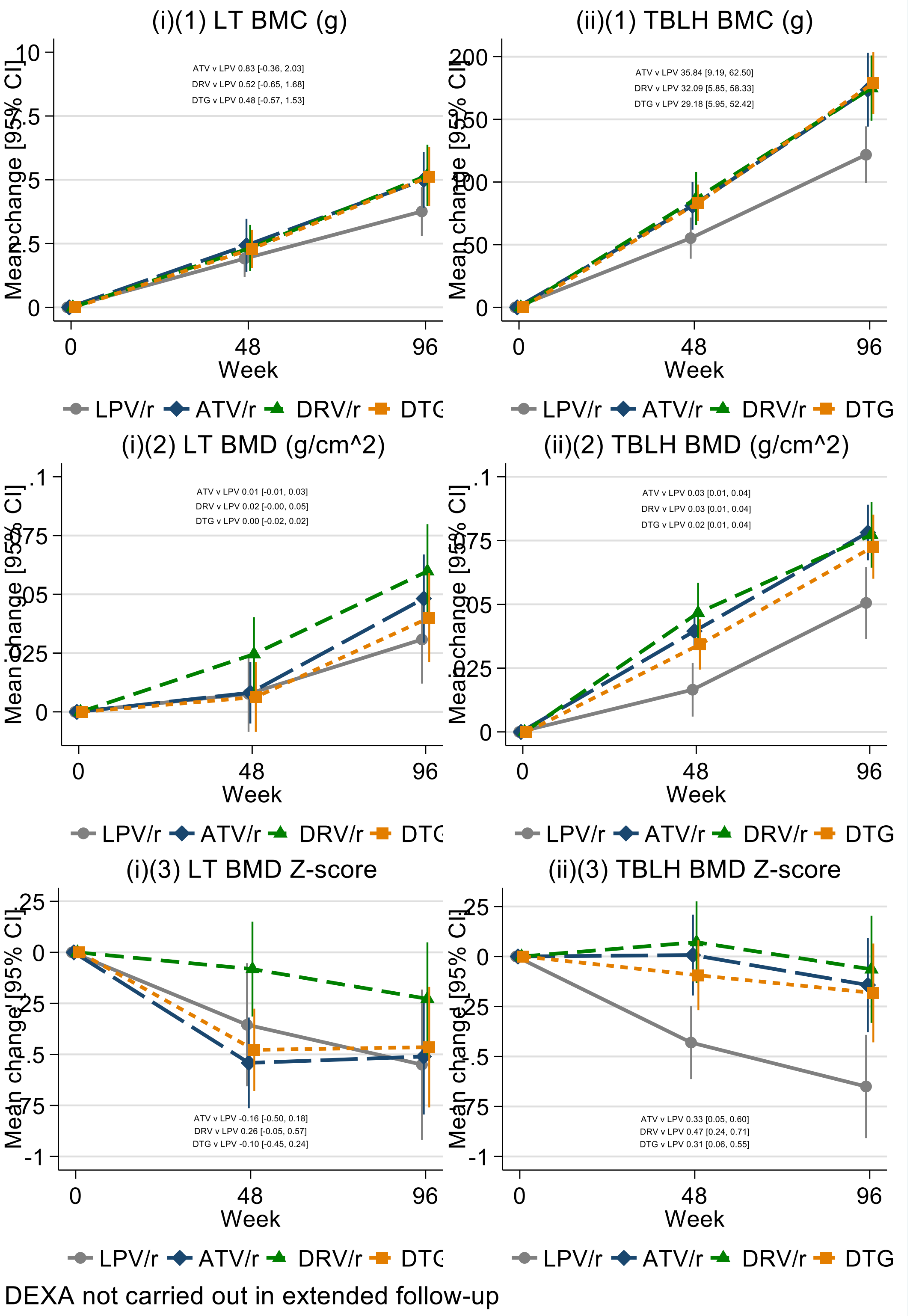

ATV/r denotes ritonavir-boosted atazanavir, BMC bone mineral content, BMD bone mineral density, DRV/r ritonavir-boosted darunavir, DTG dolutegravir, LPV/r ritonavir-boosted lopinavir, LT lumbar total and TBLH total body less head

Table S1: Representativeness of study participants

Disease, problem, or condition under investigation	Children living with HIV with first-line antiretroviral therapy (ART) failure
Special considerations related to:	
Sex and gender	The proportions of male and female children living with HIV are approximately equal in most regions of the world and there are no major difference in the prevalence of first-line virological failure relating to sex or gender
Age	Second-line ART is rarely required below the age of three years. Rates of first-line failure are lower with current integrase inhibitor-based first-line regimens than with previous non-nucleoside reverse transcriptase inhibitor based regimens.
Race or ethnic group	The large majority (nearly 87%) of children living with HIV are in sub-Saharan Africa (predominately black African)
Geography	Globally, ethnicity and age of children living with HIV varies between countries and regions and is related to factors such as local epidemiology of HIV in pregnancy and breastfeeding, access to services for prevention of vertical transmission and patterns of migration.<br><br>The majority of countries with high prevalence of HIV in children are in sub-Saharan Africa with rates of first-line failure in individual countries ranging between approximately 10 and 40%.<br><br>Rates and timing of first-line failure vary by region, country and are influenced by factors such as viral load monitoring strategy and availability of second-line ART.
Other considerations	The majority of children newly starting first-line ART or those currently on first line ART in Africa have initiated or transitioned to dolutegravir-based ART combined with abacavir and lamivudine as backbone.
Overall representativeness of this trial	Children from 6 centres in Uganda, Zimbabwe and Zambia were included in CHAPAS-4. The 6 centres included sites within and outside capital cities. Of those recruited, there was a roughly equal distribution of male and female children. The median [IQR] age was 10 [8-13] years and the majority were black African.<br><br>Children with HIV and children on ART in these three countries make up a significant proportion of both the African and global paediatric population living with HIV. The children included in CHAPAS-4 are broadly

	comparable to children living with HIV in the region in terms of sex, ethnicity and age at switch to second-line ART.<br><br>Response to second-line therapy in children is not known to vary by sex, or ethnicity in the settings included in CHAPAS-4. Observational studies have reported older age at start of second-line therapy being associated with poorer outcomes in the sub-Saharan African setting although they included a wider age range (0-<18yrs) than those included in CHAPAS-4 (3-15yrs) and no age effect was observed in our study.<br><br>As children <3 years were not included in CHAPAS-4 there are limitations in the generalisability to these younger age groups. However children in this age range rarely require second-line ART.<br><br>At the time of the trial non-nucleoside reverse transcriptase inhibitor-based first-line ART was commonly used for children. Dolutegravir has now been rolled out internationally and as a consequence the proportion of children failing first-line therapy is reducing overall, and there are fewer children failing on nonnucleoside reverse transcriptase inhibitor-based first-line ART.<br><br>However the proportion of children receiving ART that are not virologically suppressed continues to be greater than the proportion in adults. There is some way to go to achieve "95-95-95" target for children. The CHAPAS-4 trial population is representative of a substantial proportion of children globally requiring second-line ART in order to achieve the 3rd "95" (95% of those receiving ART having virological suppression).

This table was prepared using published epidemiological reports[8-10], cohort studies[11,12] and results of a randomised clinical trial of first and second-line antiretroviral therapy in children.[13]

Table S2: Weight-band based dosing

(a) Backbone drugs

WHO weight bands/kg	ABC/3TC		ZDV/3TC		FTC/TAF	
	120/60mg	600/300mg	60/30mg	300/150mg	120/15mg	200/25mg
	OD	OD	BD (am/pm)	BD (am/pm)	OD	OD
14-19.9	2.5	-	3+2*	-	1	-
20-24.9	3	-	3+3	-	1	-
25-34.9	-	1**	-	1+1	-	1
35- (adult)	-	1**	-	1+1	-	1

* or 2.5+2.5 where scored

** or 5 x ABC/3TC 120/60mg

Pharmacokinetic data for TAF from CHAPAS-4 has been published: Waalewijn H, Szubert AJ, Wasmann RE et al. Clin Infect Dis. 2023 Sep 18;77(6):875-882. [14]

ABC denotes abacavir, BD twice daily, FTC emtricitabine, NRTI nucleoside/nucleotide reverse transcriptase inhibitor, OD once daily, TAF tenofovir alafenamide fumarate, ZDV zidovudine and 3TC lamivudine

(b) Anchor drugs

WHO weight band/kg	DTG			ATV/r				LPV/r		DRV/r			
	5mg DT	50mg FCT	Daily dose/mg	200mg ATV	25mg RTV	300/100mg‡	Daily dose/mg	200/50mg*	Daily dose/mg	150mg DRV**	400mg † DRV	100mg RTV	Daily dose/mg
	OD	OD		OD	OD	OD		BD (am/pm)		OD	OD	OD	
14-19.9	5	-	25	2	3	-	200/75***	1+1	400/100	4	-	1	600/100
25-34.9	-	1	50	2	3	-	200/75***	1+1	400/100	4	-	1	600/100
25-34.9	-	1	50	-	-	1	300/100	2+1	600/150	-	2	1	800/100
35- (adult)	-	1	50	-	-	1	300/100	2+2	800/200	-	2	1	800/100

* double dose of tablets if using 100/25mg, so 2+2 for 14-19.9kg etc

** alternatively one 600mg tablet can be used which can be cut; or 8x75mg tablets if available

*** if required 100mg RTV may be used to boost ATV for the 14-19.9kg and 20-24.9kg weight-bands

†alternatively one 800mg tablet can be used which can be cut if easier to swallow.

‡ alternatively three 100mg ATV and four 25mg RTV

Pharmacokinetic data for DTG from CHAPAS-4 has been published: Waalewijn H, Wasman RE, Bamford A et al. J Pediatric Infect Dis Soc. 2024 Sep 26;13(9):496-500. [15]

Pharmacokinetic data for DRV/r from CHAPAS-4 has been published: Tsirizani L, Naghani SM, Waalewijn H et al. J Antimicrob Chemother. 2024 Sep 20:dkae319. doi: 10.1093/jac/dkae319. [16]

ATV/r denotes ritonavir-boosted atazanavir, BD twice daily, DRV/r ritonavir-boosted darunavir, DT dispersible tablet, DTG dolutegravir, FCT film-coated tablet, LPV/r ritonavir-boosted lopinavir, OD once daily, RTV ritonavir

Table S3: Additional baseline clinical and demographic characteristics

(a) Backbone randomisation

	Standard-of-care N=461	TAF N=458	Total N=919
Centre			
Uganda/Kampala	100 (21.7%)	101 (22.1%)	201 (21.9%)
Uganda/Mbarara	99 (21.5%)	97 (21.2%)	196 (21.3%)
Zambia/Lusaka	60 (13.0%)	61 (13.3%)	121 (13.2%)
Zambia/Ndola	37 (8.0%)	37 (8.1%)	74 (8.1%)
Zimbabwe/Harare	109 (23.6%)	110 (24.0%)	219 (23.8%)
Zimbabwe/Bulawayo	56 (12.1%)	52 (11.4%)	108 (11.8%)

Values are n (%) or median (IQR). TAF denotes tenofovir alafenamide fumarate

(b) Anchor randomisation

	LPV/r N=227	ATV/r N=231	DRV/r N=232	DTG N=229	Total N=919
Centre					
Uganda/Kampala	49 (21.6%)	51 (22.1%)	51 (22.0%)	50 (21.8%)	201 (21.9%)
Uganda/Mbarara	49 (21.6%)	49 (21.2%)	49 (21.1%)	49 (21.4%)	196 (21.3%)
Zambia/Lusaka	30 (13.2%)	31 (13.4%)	29 (12.5%)	31 (13.5%)	121 (13.2%)
Zambia/Ndola	18 (7.9%)	18 (7.8%)	19 (8.2%)	19 (8.3%)	74 (8.1%)
Zimbabwe/Harare	54 (23.8%)	54 (23.4%)	56 (24.1%)	55 (24.0%)	219 (23.8%)
Zimbabwe/Bulawayo	27 (11.9%)	28 (12.1%)	28 (12.1%)	25 (10.9%)	108 (11.8%)

Values are n (%) or median (IQR)

ATV/r denotes ritonavir-boosted atazanavir, DRV/r ritonavir-boosted darunavir, DTG dolutegravir and LPV/r ritonavir-boosted lopinavir

Table S4: Viral load at weeks 6, 24, 48, 72, 96, 120, 144 and 168 for thresholds of viral load <400, <60 and <1000 copies/ml

(a) Backbone randomisation

	**Standard-of-care**	**TAF**	**Total**
**<400**			
Week 6	335/404 (82.9%)	345/408 (84.6%)	680/812 (83.7%)
Week 24	343/408 (84.1%)	371/412 (90.0%)	714/820 (87.1%)
Week 48	386/453 (85.2%)	400/454 (88.1%)	786/907 (86.7%)
Week 72	358/409 (87.5%)	377/412 (91.5%)	735/821 (89.5%)
Week 96	378/454 (83.3%)	406/454 (89.4%)	784/908 (86.3%)
Week 120	175/213 (82.2%)	211/231 (91.3%)	386/444 (86.9%)
Week 144	203/249 (81.5%)	214/239 (89.5%)	417/488 (85.5%)
Week 168	150/172 (87.2%)	140/164 (85.4%)	290/336 (86.3%)
**<60**			
Week 6	200/404 (49.5%)	201/408 (49.3%)	401/812 (49.4%)
Week 24	304/408 (74.5%)	329/412 (79.9%)	633/820 (77.2%)
Week 48	335/453 (74.0%)	359/454 (79.1%)	694/907 (76.5%)
Week 72	327/409 (80.0%)	350/412 (85.0%)	677/821 (82.5%)
Week 96	332/454 (73.1%)	360/454 (79.3%)	692/908 (76.2%)
Week 120	160/213 (75.1%)	191/231 (82.7%)	351/444 (79.1%)
Week 144	177/249 (71.1%)	187/239 (78.2%)	364/488 (74.6%)
Week 168	129/172 (75.0%)	130/164 (79.3%)	259/336 (77.1%)
**<1000**			
Week 6	370/404 (91.6%)	378/408 (92.6%)	748/812 (92.1%)
Week 24	352/408 (86.3%)	374/412 (90.8%)	726/820 (88.5%)
Week 48	396/453 (87.4%)	415/454 (91.4%)	811/907 (89.4%)
Week 72	365/409 (89.2%)	385/412 (93.4%)	750/821 (91.4%)
Week 96	393/454 (86.6%)	413/454 (91.0%)	806/908 (88.8%)
Week 120	183/213 (85.9%)	218/231 (94.4%)	401/444 (90.3%)
Week 144	214/249 (85.9%)	219/239 (91.6%)	433/488 (88.7%)
Week 168	153/172 (89.0%)	146/164 (89.0%)	299/336 (89.0%)

TAF denotes tenofovir alafenamide

(b) Anchor randomisation

	LPV/r	ATV/r	DRV/r	DTG	Total
**<400**					
Week 6	151/200 (75.5%)	166/204 (81.4%)	172/208 (82.7%)	191/200 (95.5%)	680/812 (83.7%)
Week 24	173/200 (86.5%)	178/207 (86.0%)	178/208 (85.6%)	185/205 (90.2%)	714/820 (87.1%)
Week 48	179/223 (80.3%)	198/230 (86.1%)	198/229 (86.5%)	211/225 (93.8%)	786/907 (86.7%)
Week 72	171/198 (86.4%)	182/206 (88.3%)	193/212 (91.0%)	189/205 (92.2%)	735/821 (89.5%)
Week 96	180/223 (80.7%)	193/229 (84.3%)	203/230 (88.3%)	208/226 (92.0%)	784/908 (86.3%)
Week 120	88/111 (79.3%)	100/120 (83.3%)	102/110 (92.7%)	96/103 (93.2%)	386/444 (86.9%)
Week 144	95/116 (81.9%)	105/126 (83.3%)	104/120 (86.7%)	113/126 (89.7%)	417/488 (85.5%)
Week 168	65/81 (80.2%)	78/87 (89.7%)	64/77 (83.1%)	83/91 (91.2%)	290/336 (86.3%)
**<60**					
Week 6	82/200 (41.0%)	72/204 (35.3%)	78/208 (37.5%)	169/200 (84.5%)	401/812 (49.4%)
Week 24	145/200 (72.5%)	156/207 (75.4%)	162/208 (77.9%)	170/205 (82.9%)	633/820 (77.2%)
Week 48	159/223 (71.3%)	175/230 (76.1%)	172/229 (75.1%)	188/225 (83.6%)	694/907 (76.5%)
Week 72	156/198 (78.8%)	169/206 (82.0%)	172/212 (81.1%)	180/205 (87.8%)	677/821 (82.5%)
Week 96	156/223 (70.0%)	173/229 (75.5%)	175/230 (76.1%)	188/226 (83.2%)	692/908 (76.2%)
Week 120	78/111 (70.3%)	91/120 (75.8%)	91/110 (82.7%)	91/103 (88.3%)	351/444 (79.1%)
Week 144	79/116 (68.1%)	94/126 (74.6%)	91/120 (75.8%)	100/126 (79.4%)	364/488 (74.6%)
Week 168	56/81 (69.1%)	69/87 (79.3%)	57/77 (74.0%)	77/91 (84.6%)	259/336 (77.1%)
**<1000**					
Week 6	176/200 (88.0%)	190/204 (93.1%)	190/208 (91.3%)	192/200 (96.0%)	748/812 (92.1%)
Week 24	174/200 (87.0%)	181/207 (87.4%)	184/208 (88.5%)	187/205 (91.2%)	726/820 (88.5%)
Week 48	190/223 (85.2%)	205/230 (89.1%)	202/229 (88.2%)	214/225 (95.1%)	811/907 (89.4%)
Week 72	174/198 (87.9%)	190/206 (92.2%)	196/212 (92.5%)	190/205 (92.7%)	750/821 (91.4%)
Week 96	187/223 (83.9%)	199/229 (86.9%)	208/230 (90.4%)	212/226 (93.8%)	806/908 (88.8%)
Week 120	92/111 (82.9%)	105/120 (87.5%)	106/110 (96.4%)	98/103 (95.1%)	401/444 (90.3%)
Week 144	98/116 (84.5%)	111/126 (88.1%)	109/120 (90.8%)	115/126 (91.3%)	433/488 (88.7%)
Week 168	69/81 (85.2%)	78/87 (89.7%)	64/77 (83.1%)	88/91 (96.7%)	299/336 (89.0%)

ATV/r denotes ritonavir-boosted atazanavir, DRV/r ritonavir-boosted darunavir, DTG dolutegravir, LPV/r ritonavir-boosted lopinavir and VL HIV viral load

Table S5: Viral load comparisons at weeks 48, 96 and 144 for thresholds of viral load <400, <60 and <1000 copies/ml

(a) Backbone randomisation

Comparison	Week 48 (n=907): difference (%) [95% CI]	Week 96 (n=908): difference (%) [95% CI]	p*	Week 144 (n=488): difference (%) [95% CI]
**<400 copies/ml**				
TAF vs. Standard-of-care	3.0 [-1.3, 7.4]	6.3 [2.0, 10.6]	0.004	8.2 [2.1, 14.3]
**<60 copies/ml**				
TAF vs. Standard-of-care	5.3 [0.0, 10.7]	6.3 [1.0, 11.5]		7.2 [-0.2, 14.5]
**<1000 copies/ml**				
TAF vs. Standard-of-care	4.1 [0.1, 8.0]	4.6 [0.6, 8.6]		5.8 [0.4, 11.3]

*Primary endpoint only

CI denotes confidence interval and TAF tenofovir alafenamide fumarate

(b) Anchor randomisation

Comparison	Week 48 (n=907): difference (%) [95% CI]	Week 96 (n=908): difference (%) [95% CI]	P*	Week 144 (n=488): difference (%) [95% CI]
**<400 copies/ml**				
ATV/r vs. LPV/r	5.8 [-1.0, 12.6]	3.4 [-3.4, 10.2]	0.33	1.5 [-7.8, 10.7]
DTG vs. LPV/r or ATV/r	10.6 [6.0, 15.2]	9.7 [4.8, 14.5]	<0.001	7.4 [0.4, 14.4]
DRV/r vs. LPV/r or ATV/r	3.2 [-2.3, 8.8]	5.6 [0.3, 11.0]	0.04	4.3 [-3.2, 11.8]
DTG vs. DRV/r	7.3 [2.0, 12.7]	4.0 [-1.3, 9.4]		3.1 [-4.7, 11.0]
**<60 copies/ml**				
ATV/r vs. LPV/r	4.8 [-3.1, 12.7]	5.4 [-2.5, 13.2]		6.1 [-5.0, 17.2]
DTG vs. LPV/r or ATV/r	9.9 [3.8, 16.0]	10.5 [4.4, 16.6]		8.1 [-0.8, 16.9]
DRV/r vs. LPV/r or ATV/r	1.4 [-5.4, 8.1]	3.1 [-3.5, 9.8]		4.7 [-4.3, 13.8]
DTG vs. DRV/r	8.5 [1.4, 15.7]	7.4 [0.4, 14.4]		3.3 [-6.4, 12.9]
**<1000 copies/ml**				
ATV/r vs. LPV/r	3.8 [-2.2, 9.9]	2.9 [-3.5, 9.3]		3.4 [-5.1, 12.0]
DTG vs. LPV/r or ATV/r	8.0 [3.8, 12.1]	8.5 [4.1, 13.0]		5.0 [-1.4, 11.5]
DRV/r vs. LPV/r or ATV/r	1.0 [-4.2, 6.1]	4.9 [0.0, 9.9]		4.6 [-2.0, 11.3]
DTG vs. DRV/r	7.0 [2.0, 11.9]	3.6 [-1.2, 8.4]		0.2 [-6.7, 7.1]

*Primary endpoint only

ATV/r denotes ritonavir-boosted atazanavir, DRV/r ritonavir-boosted darunavir, DTG dolutegravir, LPV/r ritonavir-boosted lopinavir and VL HIV viral load

Table S6: Grade 3 and 4 adverse events during 96-week follow-up

(a) Backbone randomisation

	**Standard-of-care N=461**	**TAF N=458**	**Total N=919**	**p***
**Any**	**64 (13.9%) 93**	**63 (13.8%) 83**	**127 (13.8%) 176**	**1.00**
**CNS**	**0 (0.0%) 0**	**1 (0.2%) 3**	**1 (0.1%) 3**	**0.50**
**Psychiatric**	**3 (0.7%) 3**	**2 (0.4%) 2**	**5 (0.5%) 5**	**1.00**
**Lower Respiratory Tract**	**2 (0.4%) 2**	**3 (0.7%) 4**	**5 (0.5%) 6**	**0.69**
**Cardiovascular**	**0 (0.0%) 0**	**1 (0.2%) 1**	**1 (0.1%) 1**	**0.50**
**Eye**	**1 (0.2%) 1**	**0 (0.0%) 0**	**1 (0.1%) 1**	**1.00**
**Gastrointestinal**	**0 (0.0%) 0**	**2 (0.4%) 2**	**2 (0.2%) 2**	**0.25**
**Hepatic**	**4 (0.9%) 4**	**2 (0.4%) 2**	**6 (0.7%) 6**	**0.69**
**Musculoskeletal**	**0 (0.0%) 0**	**1 (0.2%) 1**	**1 (0.1%) 1**	**0.50**
**Skin**	**4 (0.9%) 5**	**1 (0.2%) 2**	**5 (0.5%) 7**	**0.37**
**Haematological**	**25 (5.4%) 31**	**18 (3.9%) 22**	**43 (4.7%) 53**	**0.35**
Pancytopenia, bone marrow depression	0 (0.0%) 0	1 (0.2%) 2	1 (0.1%) 2	
Anaemia with clinical symptoms	6 (1.3%) 6	1 (0.2%) 1	7 (0.8%) 7	
Thrombocytopenia	3 (0.7%) 3	4 (0.9%) 6	7 (0.8%) 9	
Leucopenia	1 (0.2%) 1	0 (0.0%) 0	1 (0.1%) 1	
Neutropenia	10 (2.2%) 10	5 (1.1%) 6	15 (1.6%) 16	
Anaemia with no clinical symptoms	8 (1.7%) 8	5 (1.1%) 5	13 (1.4%) 13	
Lymphopenia	3 (0.7%) 3	2 (0.4%) 2	5 (0.5%) 5	
**Biochemical**	**28 (6.1%) 35**	**36 (7.9%) 39**	**64 (7.0%) 74**	**0.30**
Raised liver enzymes	0 (0.0%) 0	1 (0.2%) 1	1 (0.1%) 1	
Raised AST	2 (0.4%) 2	1 (0.2%) 1	3 (0.3%) 3	
Raised ALT	1 (0.2%) 1	1 (0.2%) 1	2 (0.2%) 2	
Raised bilirubin	25 (5.4%) 32	34 (7.4%) 36	59 (6.4%) 68	
**Systemic**	**1 (0.2%) 1**	**2 (0.4%) 2**	**3 (0.3%) 3**	**0.62**
**Specific Infections**	**8 (1.7%) 8**	**0 (0.0%) 0**	**8 (0.9%) 8**	**0.008**
Herpes Zoster (Varicella Zoster) – cutaneous	1 (0.2%) 1	0 (0.0%) 0	1 (0.1%) 1	
Tuberculosis – disseminated/miliary	1 (0.2%) 1	0 (0.0%) 0	1 (0.1%) 1	
P. falciparum malaria	4 (0.9%) 4	0 (0.0%) 0	4 (0.4%) 4	

	Standard-of-care N=461	TAF N=458	Total N=919	p*
Tuberculosis – abdominal	2 (0.4%) 2	0 (0.0%) 0	2 (0.2%) 2	
**Undiagnosed Fevers**	**0 (0.0%) 0**	**1 (0.2%) 1**	**1 (0.1%) 1**	**0.50**
**Tumours**	**1 (0.2%) 1**	**0 (0.0%) 0**	**1 (0.1%) 1**	**1.00**
**Pregnancy Associated**	**0 (0.0%) 0**	**1 (0.2%) 1**	**1 (0.1%) 1**	**0.50**
**Other**	**2 (0.4%) 2**	**1 (0.2%) 1**	**3 (0.3%) 3**	**1.00**

Excluding extended follow-up after 96 weeks
Detail within body system provided where p≤0.05 or ≥10% of children experienced an event
Showing number of patients with one or more event (% of patients) number of events
TAF denotes tenofovir alafenamide fumarate
*Fisher's exact test

(b) Anchor randomisation

	LPV/r N=227	ATV/r N=231	DRV/r N=232	DTG N=229	Total N=919	p*
**Any**	**26 (11.5%) 36**	**69 (29.9%) 92**	**20 (8.6%) 28**	**12 (5.2%) 20**	**127 (13.8%) 176**	**<0.001**
**CNS**	**0 (0.0%) 0**	**0 (0.0%) 0**	**1 (0.4%) 3**	**0 (0.0%) 0**	**1 (0.1%) 3**	**1.00**
**Psychiatric**	**3 (1.3%) 3**	**1 (0.4%) 1**	**1 (0.4%) 1**	**0 (0.0%) 0**	**5 (0.5%) 5**	**0.23**
**Lower Respiratory Tract**	**2 (0.9%) 2**	**0 (0.0%) 0**	**2 (0.9%) 2**	**1 (0.4%) 2**	**5 (0.5%) 6**	**0.53**
**Cardiovascular**	**0 (0.0%) 0**	**0 (0.0%) 0**	**0 (0.0%) 0**	**1 (0.4%) 1**	**1 (0.1%) 1**	**0.50**
**Eye**	**0 (0.0%) 0**	**1 (0.4%) 1**	**0 (0.0%) 0**	**0 (0.0%) 0**	**1 (0.1%) 1**	**0.75**
**Gastrointestinal**	**1 (0.4%) 1**	**1 (0.4%) 1**	**0 (0.0%) 0**	**0 (0.0%) 0**	**2 (0.2%) 2**	**0.50**
**Hepatic**	**2 (0.9%) 2**	**3 (1.3%) 3**	**1 (0.4%) 1**	**0 (0.0%) 0**	**6 (0.7%) 6**	**0.38**
**Musculoskeletal**	**0 (0.0%) 0**	**0 (0.0%) 0**	**0 (0.0%) 0**	**1 (0.4%) 1**	**1 (0.1%) 1**	**0.50**
**Skin**	**3 (1.3%) 5**	**1 (0.4%) 1**	**1 (0.4%) 1**	**0 (0.0%) 0**	**5 (0.5%) 7**	**0.23**
**Haematological**	**13 (5.7%) 14**	**9 (3.9%) 13**	**13 (5.6%) 17**	**8 (3.5%) 9**	**43 (4.7%) 53**	**0.58**
Pancytopenia, bone marrow depression	0 (0.0%) 0	1 (0.4%) 2	0 (0.0%) 0	0 (0.0%) 0	1 (0.1%) 2	
Anaemia with clinical symptoms	3 (1.3%) 3	0 (0.0%) 0	4 (1.7%) 4	0 (0.0%) 0	7 (0.8%) 7	
Thrombocytopenia	3 (1.3%) 3	0 (0.0%) 0	2 (0.9%) 4	2 (0.9%) 2	7 (0.8%) 9	
Leucopenia	0 (0.0%) 0	1 (0.4%) 1	0 (0.0%) 0	0 (0.0%) 0	1 (0.1%) 1	
Neutropenia	3 (1.3%) 3	6 (2.6%) 6	4 (1.7%) 5	2 (0.9%) 2	15 (1.6%) 16	
Anaemia with no clinical symptoms	4 (1.8%) 4	2 (0.9%) 2	3 (1.3%) 3	4 (1.7%) 4	13 (1.4%) 13	
Lymphopenia	1 (0.4%) 1	2 (0.9%) 2	1 (0.4%) 1	1 (0.4%) 1	5 (0.5%) 5	
**Biochemical**	**3 (1.3%) 3**	**60 (26.0%) 70**	**1 (0.4%) 1**	**0 (0.0%) 0**	**64 (7.0%) 74**	**<0.001**
Raised liver enzymes	0 (0.0%) 0	1 (0.4%) 1	0 (0.0%) 0	0 (0.0%) 0	1 (0.1%) 1	
Raised AST	1 (0.4%) 1	2 (0.9%) 2	0 (0.0%) 0	0 (0.0%) 0	3 (0.3%) 3	
Raised ALT	1 (0.4%) 1	1 (0.4%) 1	0 (0.0%) 0	0 (0.0%) 0	2 (0.2%) 2	
Raised bilirubin	1 (0.4%) 1	57 (24.7%) 66	1 (0.4%) 1	0 (0.0%) 0	59 (6.4%) 68	
**Systemic**	**1 (0.4%) 1**	**0 (0.0%) 0**	**0 (0.0%) 0**	**2 (0.9%) 2**	**3 (0.3%) 3**	**0.20**
**Specific Infections**	**3 (1.3%) 3**	**0 (0.0%) 0**	**1 (0.4%) 1**	**4 (1.7%) 4**	**8 (0.9%) 8**	**0.11**
**Undiagnosed Fevers**	**0 (0.0%) 0**	**1 (0.4%) 1**	**0 (0.0%) 0**	**0 (0.0%) 0**	**1 (0.1%) 1**	**0.75**
**Tumours**	**0 (0.0%) 0**	**0 (0.0%) 0**	**0 (0.0%) 0**	**1 (0.4%) 1**	**1 (0.1%) 1**	**0.50**
**Pregnancy Associated**	**1 (0.4%) 1**	**0 (0.0%) 0**	**0 (0.0%) 0**	**0 (0.0%) 0**	**1 (0.1%) 1**	**0.25**
**Other**	**1 (0.4%) 1**	**1 (0.4%) 1**	**1 (0.4%) 1**	**0 (0.0%) 0**	**3 (0.3%) 3**	**0.90**

Excluding extended follow-up after 96 weeks

Detail within body system provided where p≤0.05 or ≥10% of children experienced an event

Showing number of patients with one or more event (% of patients) number of events
*Fisher's exact test

ALT denotes alanine aminotransferase, AST aspartate aminotransaminase, ATV/r denotes ritonavir-boosted atazanavir, CNS central nervous system, DRV/r ritonavir-boosted darunavir, DTG dolutegravir and LPV/r ritonavir-boosted lopinavir

Table S7: Serious Adverse Events (SAEs) during 96-week follow-up

(a) Backbone randomisation

	Standard-of-care N=461	TAF N=458	Total N=919	p*
**Any**	**14 (3.0%) 14**	**15 (3.3%) 17**	**29 (3.2%) 31**	**0.85**
CNS	0 (0.0%) 0	1 (0.2%) 2	1 (0.1%) 2	0.50
Upper Respiratory Tract	0 (0.0%) 0	2 (0.4%) 2	2 (0.2%) 2	0.25
Lower Respiratory Tract	2 (0.4%) 2	4 (0.9%) 4	6 (0.7%) 6	0.45
Gastrointestinal	0 (0.0%) 0	2 (0.4%) 2	2 (0.2%) 2	0.25
Hepatic	0 (0.0%) 0	1 (0.2%) 1	1 (0.1%) 1	0.50
Skin	3 (0.7%) 3	0 (0.0%) 0	3 (0.3%) 3	0.25
Haematological	2 (0.4%) 2	2 (0.4%) 3	4 (0.4%) 5	1.00
Systemic	1 (0.2%) 1	0 (0.0%) 0	1 (0.1%) 1	1.00
Specific Infections	5 (1.1%) 5	2 (0.4%) 2	7 (0.8%) 7	0.45
Undiagnosed Fevers	0 (0.0%) 0	1 (0.2%) 1	1 (0.1%) 1	0.50
Other	1 (0.2%) 1	0 (0.0%) 0	1 (0.1%) 1	1.00

Excluding extended follow-up after 96 weeks
Detail within body system provided where p≤0.05 or ≥10% of children experienced an event
Number of patients with one or more episode (% of patients) number of episodes
TAF denotes tenofovir alafenamide fumarate
*Fisher's exact test

(b) Anchor randomisation

	LPV/r N=227	ATV/r N=231	DRV/r N=232	DTG N=229	Total N=919	p*
**Any**	**10 (4.4%) 10**	**5 (2.2%) 6**	**8 (3.4%) 9**	**6 (2.6%) 6**	**29 (3.2%) 31**	**0.55**
CNS	0 (0.0%) 0	0 (0.0%) 0	1 (0.4%) 2	0 (0.0%) 0	1 (0.1%) 2	1.00
Upper Respiratory Tract	1 (0.4%) 1	1 (0.4%) 1	0 (0.0%) 0	0 (0.0%) 0	2 (0.2%) 2	0.50
Lower Respiratory Tract	3 (1.3%) 3	0 (0.0%) 0	2 (0.9%) 2	1 (0.4%) 1	6 (0.7%) 6	0.26
Gastrointestinal	1 (0.4%) 1	1 (0.4%) 1	0 (0.0%) 0	0 (0.0%) 0	2 (0.2%) 2	0.50
Hepatic	0 (0.0%) 0	1 (0.4%) 1	0 (0.0%) 0	0 (0.0%) 0	1 (0.1%) 1	0.75
Skin	2 (0.9%) 2	0 (0.0%) 0	1 (0.4%) 1	0 (0.0%) 0	3 (0.3%) 3	0.25
Haematological	1 (0.4%) 1	1 (0.4%) 2	2 (0.9%) 2	0 (0.0%) 0	4 (0.4%) 5	0.81
Systemic	0 (0.0%) 0	0 (0.0%) 0	0 (0.0%) 0	1 (0.4%) 1	1 (0.1%) 1	0.50
Specific Infections	2 (0.9%) 2	0 (0.0%) 0	1 (0.4%) 1	4 (1.7%) 4	7 (0.8%) 7	0.12
Undiagnosed Fevers	0 (0.0%) 0	1 (0.4%) 1	0 (0.0%) 0	0 (0.0%) 0	1 (0.1%) 1	0.75
Other	0 (0.0%) 0	0 (0.0%) 0	1 (0.4%) 1	0 (0.0%) 0	1 (0.1%) 1	1.00

Excluding extended follow-up after 96 weeks

Detail within body system provided where p≤0.05 or ≥10% of children experienced an event

Showing number of patients with one or more episode (% of patients) number of episodes

*Fisher's exact test

ATV/r denotes ritonavir-boosted atazanavir, DRV/r ritonavir-boosted darunavir, DTG dolutegravir and LPV/r ritonavir-boosted lopinavir

Table S8: Weight-for-age at week 96 by weight-for-age at week 0 by anchor drug

LPV/r

Week 0	<-3 N=23	-3 to <-2 N=49	-2 to <-1 N=71	-1 to <0 N=55	0 to <1 N=16	1 to <2 N=1	Total N=215
**Week 96**							
<-3	15 (65.2%)	5 (10.2%)	1 (1.4%)	0 (0.0%)	0 (0.0%)	0 (0.0%)	21 (9.8%)
-3 to <-2	8 (34.8%)	34 (69.4%)	7 (9.9%)	0 (0.0%)	0 (0.0%)	0 (0.0%)	49 (22.8%)
-2 to <-1	0 (0.0%)	9 (18.4%)	51 (71.8%)	14 (25.5%)	0 (0.0%)	0 (0.0%)	74 (34.4%)
-1 to <0	0 (0.0%)	1 (2.0%)	12 (16.9%)	36 (65.5%)	5 (31.3%)	0 (0.0%)	54 (25.1%)
0 to <1	0 (0.0%)	0 (0.0%)	0 (0.0%)	5 (9.1%)	11 (68.8%)	0 (0.0%)	16 (7.4%)
1 to <2	0 (0.0%)	0 (0.0%)	0 (0.0%)	0 (0.0%)	0 (0.0%)	1 (100.0%)	1 (0.5%)

ATV/r

Week 0	<-3 N=31	-3 to <-2 N=50	-2 to <-1 N=82	-1 to <0 N=42	0 to <1 N=13	1 to <2 N=4	Total N=222
**Week 96**							
<-3	18 (58.1%)	2 (4.0%)	0 (0.0%)	0 (0.0%)	0 (0.0%)	0 (0.0%)	20 (9.0%)
-3 to <-2	11 (35.5%)	26 (52.0%)	5 (6.1%)	0 (0.0%)	0 (0.0%)	0 (0.0%)	42 (18.9%)
-2 to <-1	2 (6.5%)	21 (42.0%)	57 (69.5%)	1 (2.4%)	0 (0.0%)	1 (25.0%)	82 (36.9%)
-1 to <0	0 (0.0%)	1 (2.0%)	19 (23.2%)	33 (78.6%)	0 (0.0%)	0 (0.0%)	53 (23.9%)
0 to <1	0 (0.0%)	0 (0.0%)	1 (1.2%)	7 (16.7%)	10 (76.9%)	1 (25.0%)	19 (8.6%)
1 to <2	0 (0.0%)	0 (0.0%)	0 (0.0%)	1 (2.4%)	2 (15.4%)	2 (50.0%)	5 (2.3%)
2 to <3	0 (0.0%)	0 (0.0%)	0 (0.0%)	0 (0.0%)	1 (7.7%)	0 (0.0%)	1 (0.5%)

DRV/r

Week 0	<-3 N=24	-3 to <-2 N=63	-2 to <-1 N=75	-1 to <0 N=44	0 to <1 N=18	1 to <2 N=1	Total N=225
**Week 96**							
<-3	11 (45.8%)	3 (4.8%)	0 (0.0%)	0 (0.0%)	0 (0.0%)	0 (0.0%)	14 (6.2%)
-3 to <-2	12 (50.0%)	34 (54.0%)	12 (16.0%)	0 (0.0%)	0 (0.0%)	0 (0.0%)	58 (25.8%)
-2 to <-1	1 (4.2%)	20 (31.7%)	48 (64.0%)	5 (11.4%)	0 (0.0%)	0 (0.0%)	74 (32.9%)
-1 to <0	0 (0.0%)	6 (9.5%)	14 (18.7%)	36 (81.8%)	4 (22.2%)	0 (0.0%)	60 (26.7%)
0 to <1	0 (0.0%)	0 (0.0%)	0 (0.0%)	3 (6.8%)	10 (55.6%)	0 (0.0%)	13 (5.8%)
1 to <2	0 (0.0%)	0 (0.0%)	1 (1.3%)	0 (0.0%)	4 (22.2%)	1 (100.0%)	6 (2.7%)

DTG

Week 0	<-3 N=26	-3 to <-2 N=61	-2 to <-1 N=73	-1 to <0 N=53	0 to <1 N=8	1 to <2 N=1	Total N=222
**Week 96**							
<-3	14 (53.8%)	3 (4.9%)	0 (0.0%)	0 (0.0%)	0 (0.0%)	0 (0.0%)	17 (7.7%)
-3 to <-2	11 (42.3%)	30 (49.2%)	6 (8.2%)	0 (0.0%)	0 (0.0%)	0 (0.0%)	47 (21.2%)
-2 to <-1	1 (3.8%)	25 (41.0%)	42 (57.5%)	10 (18.9%)	0 (0.0%)	0 (0.0%)	78 (35.1%)
-1 to <0	0 (0.0%)	3 (4.9%)	22 (30.1%)	34 (64.2%)	3 (37.5%)	0 (0.0%)	62 (27.9%)
0 to <1	0 (0.0%)	0 (0.0%)	2 (2.7%)	7 (13.2%)	4 (50.0%)	0 (0.0%)	13 (5.9%)
1 to <2	0 (0.0%)	0 (0.0%)	1 (1.4%)	2 (3.8%)	1 (12.5%)	1 (100.0%)	5 (2.3%)

ATV/r denotes ritonavir-boosted atazanavir, DRV/r ritonavir-boosted darunavir, DTG dolutegravir and LPV/r ritonavir-boosted lopinavir

Table S9: Number of disease progression events of each type (anchor randomisation)

	LPV/r	ATV/r	DRV/r	DTG
WHO 3	0 (0.0%) 0	0 (0.0%) 0	0 (0.0%) 0	1 (0.4%) 1
WHO 4	2 (0.9%) 2	1 (0.4%) 1	2 (0.9%) 2	3 (1.3%) 3
Death	0 (0.0%)	0 (0.0%)	0 (0.0%)	1 (0.4%)
Any	2 (0.9%) 2	1 (0.4%) 1	2 (0.9%) 2	4 (1.7%) 5

Showing Number of patients with one or more event (% of patients) number of events
e.g., '2 (20.0%) 3,' would indicate a total of 3 events in a total of 2 patients
Corresponding hazard ratios and 95% confidence intervals:
ATV/r v LPV/r: 0.49 [0.04, 5.39]; DRV/r v LPV/r: 0.98 [0.14, 6.93]; DTG v LPV/r: 1.99 [0.36, 10.87]

ATV/r denotes ritonavir-boosted atazanavir, DRV/r ritonavir-boosted darunavir, DTG dolutegravir and LPV/r ritonavir-boosted lopinavir

Cost-Effectiveness Supplemental Methods

The health economics analysis assessed the costs and cost-effectiveness of two sets of comparisons from the CHAPAS-4 trial over the 96-week trial period: i) for the NRTI backbone randomization, tenofovir alafenamide fumarate(TAF)/emtricitabine (FTC) was compared to standard-of-care (SOC); and ii) for the randomization of second line anchor drugs, ,there were five comparisons ((1) dolutegravir (DTG) vs. ritonavir-boosted atazanavir (ATV/r) or ritonavir-boosted lopinavir (LPV/r); (2) ritonavir-boosted darunavir (DRV/r) vs. ATV/r or LPV/r; (3) ATV/r vs. LPV/r; (4) DTG vs. ATV/r or LPV/r or DRV/r; (5) DTG vs. ATV/r vs. LPV/r vs. DRV/r). Health was measured by quality adjusted life years (QALYs) and was calculated using the EQ-5D-3L instrument and the area-under-the-curve approach. Total costs were estimated from the health system perspective and included antiretroviral (ART) drugs costs, clinic visits costs and hospital stays costs. A discount rate of 3% per annum was applied to costs and QALYs incurred for participants in their second year of the study. All costs were measured in 2022 US$.

Generalized linear models with a gamma distribution and an identity link function were used to estimate the mean total costs and incremental costs and ordinary least squares were used to estimate the mean QALYs and the incremental QALYs. The following stratification factors were controlled for in all regression models: the six sites and the nucleos(t)ide reverse transcriptase inhibitor (NRTI) backbone drugs they failed on in the first line treatment. The baseline EQ5D indices were also controlled for in estimating total and incremental QALYs. Probabilistic sensitivity analyses were conducted to estimate the probability of each intervention being least costly (in cost analysis) or most cost-effective (in cost-effectiveness analysis).

Cost-effectiveness Supplemental Results

Unit costs of ART drugs and of other health care are reported in Table CES1a (NRTI backbone), CES1b (second line anchor drug) and CES2, respectively.

Table CES3a and CES4a report the resources used, and costs and QALYs for the TAF/FTC compared to SOC. Table CES3b and CES4b report the resources used, and costs and QALYs for the second line anchor comparators.

The main health economics analyses focused on comparing the mean total costs between trial arms for the 96-week trial period, including costs of ART drugs, clinic visits and hospital stays, from the health system perspective. QALYs outcomes calculated based on the EQ-5D-3L instrument were not significantly different across trial arms and there were no systematic trends between arms suggesting any small observed differences were the result of random chance.

Table CES5a reports the estimated total costs of SOC and TAF/FTC, the estimated incremental cost of TAF/FTC, and the probability of each being least costly. TAF/FTC was found to be $37.68 less costly than SOC. Probabilistic sensitivity analysis suggested the probability of TAF/FTC being cost saving compared to SOC was 100%. Table CES6a reports the full cost-effectiveness analysis including the estimated total QALYs of SOC and TAF/FTC, and incremental cost-effectiveness ratios (ICERs) and incremental net health benefits (INHBs) of TAF/FTC compared to SOC. TAF/FTC was less costly ($37.68) than SOC, it was also marginally less effective (0.0048 QALYs). One QALY loss from switching from SOC to TAF/FTC generated $7,861 of cost savings, considerably higher than the highest cost-effectiveness threshold of $500 per QALY. The INHB of TAF/FTC compared to SOC was 0.0707, using the cost-effectiveness threshold of $500 per QALY. The positive net benefit suggested that TAF/FTC was cost-effective compared to SOC. The probability of TAF/FTC being cost-effective was 100% using three different threshold levels of £100, £300 and £500 per QALY.

Table CES5b presents mean total costs and incremental costs for each of the second line anchor comparisons. The four-way comparisons of DTG vs. LPV/r vs. ATV/r vs. DRV/r (comparison 5) showed that DTG was the least costly, and DRV/r most costly. The probability of DTG being cost-saving compared to the other three anchor drugs was 100%. Switching to DTG from the next most costly option, ATV/r, generated a large cost saving of $190.77. Other two-way comparisons suggested that ATV/r was cost-saving compared to LPV/r (saving of $17.35). DTG was cost-saving compared to ATV/r or LPV/r (cost saving of $200.49). DRV/r was around $130.10 more costly than ATV/r or LPV/r. Table CES6b presents the estimated total costs and total QALYs, and ICERs and INHBs for each of the comparisons. The four-way comparisons of DTG vs. LPV/r vs. ATV/r vs. DRV/r showed that LPV/r and DRV/r were dominated by ATV/r. Although ATV/r was more effective (0.0093 QALY), it was also more costly ($190.77) than DTG and the ICER of $20,423 per QALY was much higher than the cost-effectiveness thresholds of £500 per QALY considered here. INHBs of ATV/r, LPV/r and DRV/r compared to DTG were all negative using three different cost-effectiveness threshold levels of £100, £300 and £500 per QALY, suggesting that DTG was the cost-effective option among the four second-line drugs compared. Probabilistic sensitivity analyses also showed that ATV/r, LPV/r or DRV/r had 0% of being cost-effective when compared with DTG. Other two-way comparisons suggested that ATV/r was cost-effective compared to LPV/r with a probability of 99.70%, as it is more effective in terms of QALYs (0.0018 QALY) and slightly less costly ($17.35). DRV/r was dominated by ATV/r or LPV/r being less effective (0.0029 QALY) in terms of QALYs and more costly ($130.10).

Table CES1a: Costs of NRTI backbone antiretroviral drugs

		WHO WEIGHT BANDS			
		14-19.9 kg	20-24.9 kg	25-34.9 kg	35- kg (adult)
**Abacavir/ Lamivudine ABC/3TC**	Daily dose	300/150 mg	360/180 mg	600/300 mg	600/300 mg
	Tablets taken	2.5*120/60 mg ABC/3TC	3*120/60 mg ABC/3TC	1*600/300 mg[1] ABC/3TC	1*600/300 mg[1] ABC/3TC
	**Unit cost($)**	**ABC/3TC (120/60 mg) disp. scored: 0.1**	**ABC/3TC (120/60 mg) disp. scored: 0.1**	**ABC/3TC (600/300 mg): 0.25**	**ABC/3TC (600/300 mg): 0.25**
	**Daily cost($)**	**0.25**	**0.3**	**0.25**	**0.25**
**Zidovudine/ Lamivudine ZDV/3TC**	Daily dose	300/150 mg	360/180 mg	600/300mg	600/300mg
	Tablets taken	3*60/30 mg ZDV/3TC (AM) + 2*60/30 mg ZDV/3TC[2] (PM)	3*60/30 mg ZDV/3TC (AM) + 3*60/30 mg ZDV/3TC[2] (PM)	1*300/150 mg ZDV/3TC (AM) + 1*300/150 mg ZDV/3TC (PM)	1*300/150 mg ZDV/3TC (AM) + 1*300/150 mg ZDV/3TC (PM)
	**Unit cost($)**	**ZDV/3TC (60/30 mg) disp. scored: 0.03**	**ZDV /3TC (60/30 mg) disp. scored: 0.03**	**ZDV /3TC (300/150 mg): 0.09**	**ZDV /3TC (300/150 mg): 0.09**
	**Daily cost($)**	**0.15**	**0.18**	**0.18**	**0.18**
**Tenofovir alafenamide/ Emtricitabine TAF/FTC**	Daily dose	120/15 mg	120/15 mg	200/25 mg	200/25 mg
	Tablets taken	1*120/15 mg FTC/TAF	1*120/15 mg FTC/TAF	1*200/25 mg FTC/TAF	1*200/25 mg FTC/TAF
	**Unit cost($)**	**TAF/FTC/DTG (25/200/50 mg): 0.17**	**TAF/FTC/DTG (25/200/50 mg): 0.17**	**TAF/FTC/DTG (25/200/50 mg): 0.17**	**TAF/FTC/DTG (25/200/50 mg): 0.17**
	**Daily cost($)**	**0.102**	**0.102**	**0.17**	**0.17**

Notes: ART costs were calculated using data on usage from CHAPAS-4 trial and unit costs of different ART drugs from Clinton Health Access Initiative (CHAI) where available (2022). Where data was unavailable, trial sites provided information on costs. We used proportions (calculated based on dosage) of the published adult drug costs for the costs of the paediatric drugs 120/15 mg FTC/TAF. ABC: abcavir, 3TC: lamivudine, ZDV: zidovudine, TAF: tenofovir

alafenamide, FTC: emtricitabine, DTG: dolutegravir 1. or 5 x ABC/3TC 120/60mg; 2. or 2.5+2.5; 3. Tablets taken: once daily if not stated, or twice daily (morning **AM** and afternoon **PM**)

Table CES1b: Costs of antiretroviral anchor drugs

<table>
<tr><th></th><th></th><th colspan="5">WHO weight bands</th></tr>
<tr><th></th><th></th><th colspan="2">14-19.9 kg</th><th>20-24.9 kg</th><th>25-34.9 kg</th><th>35- kg (adult)</th></tr>
<tr><td rowspan="4">Dolutegravir
(DTG)</td><td>Daily dose</td><td colspan="2">25 mg DT</td><td>50 mg FCT</td><td>50 mg FCT</td><td>50 mg FCT</td></tr>
<tr><td>Tablets taken</td><td colspan="2">5*5 mg DT</td><td>1*50 mg FCT</td><td>1*50 mg FCT</td><td>1*50 mg FCT</td></tr>
<tr><td>Unit cost($)</td><td>2.5*DTG (10 mg) disp. scored: 0.05</td><td>half a DTG (50 mg) FCT: 0.07</td><td>DTG (50 mg): 0.07</td><td>DTG (50 mg): 0.07</td><td>DTG (50 mg): 0.07</td></tr>
<tr><td>Daily cost($)</td><td>0.125</td><td>0.035</td><td>0.07</td><td>0.07</td><td>0.07</td></tr>
<tr><td rowspan="5">Atazanavir/
ritonavir
(ATV/r)</td><td>Daily dose</td><td colspan="2">200/75[1] mg</td><td>200/75[1] mg</td><td>300/100 mg</td><td>300/100 mg</td></tr>
<tr><td>Tablets taken</td><td colspan="2">2*100 mg ATV, 3*25 mg RTV</td><td>2*100 mg ATV, 3*25 mg RTV</td><td>1*300/100 mg[2]</td><td>1*300/100 mg[2]</td></tr>
<tr><td>Unit cost($)</td><td colspan="2">ATV 200mg capsule: 0.59, RTV (25 mg) heat-stable: 0.10</td><td>ATV 200mg capsule: 0.59, RTV (25 mg) heat-stable: 0.10</td><td>ATV/r (300/100 mg): 0.41
ATV 300mg capsule:0.61
RTV (100 mg) heat-stable: 0.10</td><td>ATV/r (300/100 mg): 0.41
ATV 300mg capsule:0.61
RTV (100 mg) heat-stable: 0.10</td></tr>
<tr><td>Daily cost($)</td><td colspan="2">0.89</td><td>0.89</td><td>0.41</td><td>0.41</td></tr>
<tr><td>paediatric drug cost as proportion of adult drug cost</td><td colspan="2">0.30</td><td>0.30</td><td>0.41</td><td>0.41</td></tr>
</table>

**Lopinavir/ritonavir (LPV/r)**	Daily dose	400/100 mg	400/100 mg	600/150 mg	800/200 mg
	Tablets taken	1*200/50 mg[3] (AM) + 1*200/50 mg[3] (PM)	1*200/50 mg[3] (AM) + 1*200/50 mg[3] (PM)	2*200/50 mg[3] (AM) + 1*200/50 mg[3] (PM)	2*200/50 mg[3] (AM) + 2*200/50 mg[3] (PM)
	**Unit cost($)**	**LPV/r (200/50 mg): 0.14**	**LPV/r (200/50 mg): 0.14**	**LPV/r (200/50 mg): 0.14**	**LPV/r (200/50 mg): 0.14**
	**Daily cost($)**	**0.28**	**0.28**	**0.42**	**0.56**
**Darunavir/ritonavir (DRV/r)**	Daily dose	600/100 mg	600/100 mg	800/100 mg	800/100 mg
	Tablets taken	4*150 mg[4] DRV, 1*100 mg RTV	4*150 mg[4] DRV, 1*100 mg RTV	2*400 mg[5] DRV, 1*100 mg RTV	2*400 mg[5] DRV, 1*100 mg RTV
	**Unit cost($)**	**DRV (150 mg): 0.21, RTV (100 mg) heat-stable: 0.10**	**DRV (150 mg): 0.21, RTV (100 mg) heat-stable: 0.10**	**DRV/r (400/50 mg): 0.32**	**DRV/r (400/50 mg): 0.32**
	**Daily cost($)**	**0.94**	**0.94**	**0.64**	**0.64**
	**paediatric drug cost as proportion of adult drug cost**	**0.48**	**0.48**	**0.64**	**0.64**

Notes: ART costs were calculated using data on usage from CHAPAS-4 trial and unit costs of different ART drugs from Clinton Health Access Initiative (CHAI) where available (2022). Where data was unavailable, trial sites provided information on costs. In some cases, we used proportions of the published adult drug costs for the costs of their paediatric drugs (e.g. 200/75 mg ATV/r, 600/100 mg DRV/r). 1. if required 100mg RTV may be used to boost ATV for the 14-19.9kg and 20-24.9kg weight-bands; 2. alternatively three 100mg ATV and four 25mg RTV; 3. double dose of tablets if using 100/25mg, so 2+2 for 14-19.9kg etc; 4. alternatively one 600mg tablet can be used which can be cut; or 8x75mg tablets if available. DT: dispersible tablet, FCT: film coated tablet, DTG: dolutegravir, ATV/r: ritonavir-boosted atazanavir, LPV/r: ritonavir-boosted lopinavir, DRV/r: ritonavir-boosted darunavir, RTV: ritonavir. Tablets taken: once daily if not stated, or twice daily (morning **AM** and afternoon **PM**)

Table CES2: Costs of health care resources used

	Uganda		Zambia		Zimbabwe	
	2022 price in US$ (adjusted for local inflation)	Source	2022 price in US$ (adjusted for local inflation)	Source	2022 price in US$ (adjusted for Zambia's inflation as a proxy)	Source
Hospital overnight: Teaching hospital	4.81	WHO Choice (2011)	8.83	WHO Choice (2011)	3.93	WHO Choice (2011)
Outpatient attendances: Health Centre (no beds)	0.97		1.78		0.75	
Outpatient attendances: Secondary-level hospital	1.42		2.60		1.09	

Notes: 1. Unit costs of outpatient attendances in a secondary-level hospital (the highest level) were used to cost scheduled and unscheduled visits, unit costs of hospital overnight in a teaching hospital (the highest level) were used to cost hospital stays, and unit costs of outpatient attendances in a health centre (no beds) were used to calculated the costs of visiting a local clinic or healthcare workers; 2. All unit costs were checked with the CHAPAS-4 trial sites to confirm that they were reasonable estimates of the actual costs; 3. Unit cost information collected before 2022 were adjusted for local inflation to get the 2022 price (the unit cost was reported in US dollar in WHO Choice (2011).

Table CES3a. Resource Use by NRTI backbone randomization groups.

NRTI Backbone Randomization Groups	SOC		TAF/FTC	
	Mean	SD	Mean	SD
ART use				
Average duration of four anchor drugs (days)	662.30	57.79	666.07	47.93
LPV/r duration (days)	651.90	85.91	652.91	85.07
As % of the 96-week trial on LPV/r	97.01%	12.78%	97.16%	12.66%
N (as % of the 919 children)	115 (12.51%)		112 (12.19%)	
ATV/r duration (days)	655.89	55.37	664.06	29.46
As % of the 96-week trial on ATV/r	97.60%	8.24%	98.82%	4.38%
N (as % of the 919 children)	115 (12.51%)		116 (12.62%)	
DRV/r duration (days)	658.16	69.81	659.96	67.26
As % of the 96-week trial on DRV/r	97.94%	10.39%	98.21%	10.01%
N (as % of the 919 children)	114 (12.4%)		118 (12.84%)	
DTG duration (days)	667.74	23.38	649.66	99.44
As % of the 96-week trial on DTG	99.37%	3.48%	96.68%	14.80%
N (as % of the 919 children)	117 (12.73%)		112 (12.19%)	
Average duration of NRTI backbone drugs (days)	661.78	56.83	663.79	54.49
Average duration of other ART drugs (days)	0.24	5.22	2.80	42.36
Other health care use				
Scheduled clinic visits	10.76	0.87	10.82	0.67
Unscheduled clinic visits	1.22	2.27	0.97	1.85
Hospital stays (days)	0.24	2.40	0.19	1.49
Visits to a local clinic or healthcare workers	0.17	0.50	0.27	1.18
N (as % of the 919 children)	461 (50.16%)		458 (49.84 %)	

Notes: 1. The days in four anchor drugs were calculated conditional on patients being allocated to the specific anchor drugs at randomization; 2. We only calculated the resources used when patients were on second-line treatment; 3. Four anchor drugs included LPV/r, ATV/r, DRV/r, DTG; 4. NRTI backbone drugs included TAF/FTC and SoC (ABC/3TC or ZDV/3TC); 5. Other ART drugs included TDF/3TC/DTG (300/300/50 mg) and TDF/3TC. NRTI: Nucleos(t)ide reverse transcriptase inhibitor, SOC: standard-of-care, TAF: tenofovir alafenamide, FTC: emtricitabine, SD: standard deviation, ART: antiretroviral therapy, LPV/r: ritonavir-boosted lopinavir, ATV/r: ritonavir-boosted atazanavir, DRV/r: ritonavir-boosted darunavir, DTG: dolutegravir.

Table CES3b. Resource use by anchor drug randomization groups

Anchor drug Randomization groups	LPV/r		ATV/r		DRV/r		DTG	
	Mean	SD	Mean	SD	Mean	SD	Mean	SD
ART use								
Average duration of four anchor drugs (days)	660.28	68.79	666.78	38.01	667.38	39.07	662.17	60.21
Average duration of NRTI backbone drugs (days)	656.33	77.67	665.57	34.37	666.72	39.78	662.39	60.26
SOC duration (days)	641.40	119.18	660.5	48.46	658.66	72.93	668.15	23.28
As % of the 96-week trial on SoC	0.9545	0.1773	0.9829	0.0721	0.9801	0.1085	0.9943	0.0346
N (as % of the 919 children)	115	12.51%	115	12.51%	114	12.40%	117	12.73%
TAF/FTC duration (days)	658.21	71.59	669.66	9.33	670.37	4.22	656.38	82.58
As % of the 96-week trial on TAF/ FTC	0.9795	0.1065	0.9965	0.0139	0.9976	0.0063	0.9767	0.1229
N (as % of the 919 children)	112	12.19%	116	12.62%	118	12.84%	112	12.19%
Average duration of other ART drugs (days)	2.69	40.49	2.91	44.21	0.48	7.35	0.00	0.00
Other health care use								
Scheduled clinic visits	10.72	0.96	10.84	0.56	10.83	0.73	10.78	0.80
Unscheduled clinic visits	1.22	2.10	1.29	2.17	0.99	1.75	0.87	2.24
Hospital stays (days)	0.19	1.13	0.16	1.29	0.28	1.80	0.24	3.14
Visits to a local clinic or healthcare workers	0.22	0.63	0.25	1.42	0.20	0.60	0.22	0.72
N (as % of the 919 children)	227	(24.70%)	231	(25.14%)	232	(25.24%)	229	(24.92%)

Notes: 1. The days in four anchor drugs were calculated conditional on patients being allocated to the specific anchor drugs at randomization; 2. We only calculated the resource use when patients were on second-line treatment; 3. Anchor drugs included LPV/r, ATV/r, DRV/r, DTG; 4. NRTI backbone drugs included TAF/FTC and SOC (ABC/3TC or ZDV/3TC); 5. Other ART drugs included TDF/3TC/DTG (300/300/50 mg) and TDF/3TC. DTG: dolutegravir, ATV/r: ritonavir-boosted atazanavir, LPV/r: ritonavir-boosted lopinavir, DRV/r: ritonavir-boosted darunavir, ART: antiretroviral therapy, NRTI: nucleos(t)ide revirse transcriptase inhibitor, SD: standard deviation, SOC: stand-of-care, TAF: tenofovir alafenamide, FTC: emtricitabine, ABC: abacavir, 3TC: lamivudine.

Table CES4a. Costs and QALYs by NRTI backbone randomization groups

NRTI backbone randomization groups	SOC		TAF/FTC	
Costs	Mean	SD	Mean	SD
ART costs				
Anchor drugs costs	236.01	131.74	244.79	132.94
DTG cost	13.29	23.53	12.19	21.81
ATV/r cost	59.69	105.63	61.79	107.67
LPV/r cost	66.55	120.08	68.46	122.96
DRV/r cost	93.31	165.43	99.05	170.73
NRTI backbone drugs costs	140.43	32.37	98.91	21.70
Other ART drug costs	0.03	0.68	0.30	4.48
**Total ART costs**	**371.39**	**136.78**	**339.35**	**137.49**
Other health care costs				
Scheduled visits costs	14.94	5.52	15.09	5.53
Unscheduled visits costs	1.59	2.84	1.28	2.32
Hospital stay costs	1.30	11.07	0.94	6.89
Cost of visiting a local clinic or healthcare workers	0.23	0.75	0.31	1.10
**Total other health care costs**	**18.05**	**14.06**	**17.61**	**10.20**
**Total health care costs**	**389.35**	**136.95**	**356.97**	**138.52**
**QALYs**	**1.8035**	**0.0285**	**1.7992**	**0.0873**
N (as % of the 919 children)	461 (50.16%)		458 (49.84 %)	

Notes: 1. All the costs were in 2022 US dollar; 2. The discount factor for costs and QALYs occurring in in the second year of the study was 3% per annum; 3. Anchor drugs included DTG , ATV/r, LPV/r, DRV/r; 4. NRTI backbone drugs included TAF/FTC and SoC (ABC/3TC or ZDV/3TC); 5. Other ART drugs included TDF/3TC/DTG (300/300/50 mg) and TDF/3TC; 6. QALYs were captured by EQ-5D index over 96 weeks using area-under-the-curve approach. Only EQ-5D indices reported in the scheduled visits were used to calculate QALYs. Missing baseline indices were imputed using the sample average index, while other missing indices were imputed using the mean of the indices before and after the missing index of the same individual. NRTI: Nucleos(t)ide reverse transcriptase inhibitor, SOC: standard-of-care, TAF: tenofovir alafenamide, FTC: emtricitabine, SD: standard deviation, ART: antiretroviral therapy, LPV/r: ritonavir-boosted lopinavir, ATV/r: ritonavir-boosted atazanavir, DRV/r: ritonavir-boosted darunavir, DTG: dolutegravir, QALY: quality-adjusted life year

Table CES4b. Costs and QALYs by anchor drug randomization groups

Anchor Drug Randomization Groups	LPV/r		ATV/r		DRV/r		DTG	
Costs	Mean	SD	Mean	SD	Mean	SD	Mean	SD
ART costs								
Anchor drugs costs	269.87	77.25	247.13	36.81	390.94	57.15	51.84	15.57
NRTI backbone drugs costs	117.76	37.03	120.16	34.31	118.87	33.59	122.14	33.08

TAF/FTC cost	48.07	50.68	48.90	50.76	50.00	51.02	48.69	51.98
SOC cost	68.11	72.88	69.65	73.46	67.28	72.40	71.80	73.40
Other ART drug costs	0.30	4.45	0.30	4.51	0.06	0.96	0.00	0.00
**Total ART costs**	**382.69**	**96.45**	**362.62**	**56.17**	**502.98**	**78.33**	**171.67**	**34.55**
Other health care costs								
Scheduled visits costs	14.88	5.50	15.06	5.42	15.02	5.56	15.11	5.65
Unscheduled visits costs	1.61	2.67	1.70	2.87	1.30	2.16	1.12	2.61
Hospital stay costs	1.31	7.83	0.69	5.30	1.42	9.32	1.08	12.85
Cost of visiting a local clinic or healthcare workers	0.27	0.84	0.24	1.13	0.27	0.84	0.29	0.94
**Total other health care costs**	**18.06**	**11.22**	**17.69**	**9.31**	**18.00**	**11.90**	**17.59**	**15.86**
**Total health care costs**	**400.73**	**97.30**	**380.31**	**56.02**	**520.81**	**79.06**	**189.26**	**38.88**
**QALYs**	**1.8047**	**0.0271**	**1.8045**	**0.0395**	**1.8018**	**0.0304**	**1.7945**	**0.1168**
N (as % of the 919 children)	227	(24.70%)	231	(25.14%)	232	(25.24%)	229	(24.92%)

Notes: 1. All the costs were in 2022 US dollar; 2. The discount factor for costs and QALYs occurring in the second year of the study was 3% per annum; 3. Anchor drugs included LPV/r, ATV/r, DRV/r, DTG; 4. NRTI backbone drugs included TAF/FTC, ABC/3TC, ZDV/3TC; 5. Other ART drugs included TDF/3TC/DTG (300/300/50 mg) and TDF/3TC; 6. QALYs were captured by EQ-5D index over 96 weeks using area-under-the-curve approach. Only EQ-5D indices reported in the scheduled visits were used to calculate QALYs. Missing baseline indices were imputed using the sample average index, while other missing indices were imputed using the mean of the indices before and after the missing index of the same individual. DTG: dolutegravir, ATV/r: ritonavir-boosted atazanavir, LPV/r: ritonavir-boosted lopinavir, DRV/r: ritonavir-boosted darunavir, ART: antiretroviral therapy, NRTI: nucleos(t)ide revirse transcriptase inhibitor, SD: standard deviation, SOC: stand-of-care, TAF: tenofovir alafenamide, FTC: emtricitabine, ABC: abacavir, 3TC: lamivudine, QALY: quality-adjusted life year,

Table CES5a. Costs Analysis of NRTI Backbone Randomization Groups

Comparators	Total Costs	Incremental cost				Probability of being least costly
	mean	mean	SE	95% CI		
SOC	391.61					0%
TAF/FTC	353.92	-37.68	3.39	-44.32	-31.05	100%
N=919						

Notes: 1. All the costs were in 2022 US dollar and a discount rate of 3% per annum was applied to costs incurred for participants in their second year of the study; 2. The model controlled for stratification factors of the six sites and the NRTI backbone drugs they failed on in the first line treatment; 3. Probabilistic sensitivity analyses were conducted to estimate the probability of each intervention being least costly using the threshold of $500 per QALY implied by the decisions that had been previously made on the ART drugs for HIV. SOC: standard-of-care, TAF: tenofovir alafenamide, FTC: emtricitabine, SE: standard error, CI: confidence interval.

Table CES5b. Costs Analysis of Different Comparators for Anchor Drug Randomization Groups

Comparators	Costs	Incremental cost				Probability of being least costly
	mean	mean	SE	95% CI		
(1) ATV/r or LPV/r	389.89					0%
DTG	189.40	-200.49	4.03	-208.39	-192.58	100%
N=687						
(2) ATV/r or LPV/r	390.41					100%
DRV/r	520.50	130.10	6.63	117.10	143.10	0%
N=690						
(3) LPV/r	399.07					0.4%
ATV/r	381.72	-17.35	6.69	-30.45	-4.25	99.6%
N=458						
(4) ATV/r or LPV/r or DRV/r	433.66					0%
DTG	189.56	-244.09	4.34	-252.60	-235.58	100%
N=919						
Comparators	Costs	incremental cost compared to the next lowest cost				Probability of being least costly
	mean	mean	SE	95% CI		
(5) DTG	189.58					100%
ATV/r	380.35	190.77	4.76	181.45	200.10	0%
LPV/r	399.20	18.85	6.22	6.67	31.03	0%
DRV/r	520.41	121.21	7.38	106.73	135.68	0%
N=919						

Notes: 1. All the costs were in 2022 US dollar and a discount rate of 3% per annum was applied to costs incurred for participants in their second year of the study; 2. The model controlled for stratification factors of the six sites and the NRTI backbone drugs they failed on in the first line treatment; 3. Probabilistic sensitivity analyses were conducted to estimate the probability of each intervention being least costly using the threshold of $500 per QALY implied by the decisions that had been previously made on the ART drugs for HIV; 4. There were five comparators of different anchor drugs: (1) DTG vs. ATV/r or LPV/r; (2) DRV/r vs. ATV/r or LPV/r; (3) ATV/r vs. LPV/r; (4) DTG vs. ATV/r or LPV/r or DRV/r. (5) DTG vs. ATV/r vs. LPV/r vs. DRV/r. DTG: dolutegravir, ATV/r: ritonavir-boosted atazanavir, LPV/r: ritonavir-boosted lopinavir, DRV/r: ritonavir-boosted darunavir, ART: antiretroviral therapy, NRTI: nucleos(t)ide revirse transcriptase inhibitor, SE: standard error, CI: confidence interval.

Table CES6a. Cost-effectiveness Analysis of NRTI Backbone Randomization Groups

Comparators	Mean Cost	Mean QALYs	Incremental cost				Incremental QALYs				ICER	INHB (λ=100)	Prob of CE	INHB (λ=300)	Prob of CE	INHB (λ=500)	Prob of CE
	mean	mean	mean	SE	95% CI		mean	SE	95% CI								
SOC	391.61	1.8037											0%		0%		0%
TAF/FTC	353.92	1.7990	-37.68	3.39	-44.32	-31.05	-0.0048	0.0041	-0.0129	0.0033	7860.87	0.3728	100%	0.1211	100%	0.0707	100%
N=919																	

Notes: 1. All the costs were in 2022 US dollar and the discount factor for costs and QALYs occurring in in the second year of the study was 3% per annum; 2. Generalized linear model with a gamma distribution of the dependent variable and an identity link function was used to predict the mean total costs and estimate the incremental costs, controlling for the stratification factors; 3. Generalized linear model with a gaussian (normal) distribution of the dependent variable and an identity link function (equivalent to ordinary least squares) was used to predict the mean QALYs and estimate the incremental QALYs, controlling for the stratification factors and the baseline EQ5D indices; 4. Cost-effectiveness threshold of $500 per QALY was used to calculate ICER based on the decisions that had been previously made on the ART drugs for HIV; 5. cost-effectiveness thresholds of $100 and $300 per QALY were also used when calculating INHB for scenario analyses, and probabilistic sensitivity analyses were conducted to estimate the probability of each intervention being cost-effective (CE) using the three thresholds of $500, $300 and $100 per QALY. QALY: quality-adjusted life year, INHB: incremental net health benefit, CE: cost effectiveness.

Table CES6b. Cost-effectiveness Analysis of Different Comparators for Anchor Drug Randomization Groups

Comparators	Total Cost	Total QALYs	Incremental cost				Incremental QALYs				ICER	INHB (λ=100)	Prob of being CE	INHB (λ=300)	Prob of being CE	INHB (λ=500)	Prob of being CE
	mean	mean	mean	SE	95% CI		mean	SE	95% CI								
(1) ATV/r or LPV/r	389.89	1.8040											0%		0%		0%
DTG	189.40	1.7957	-200.49	4.03	-208.39	-192.58	-0.0083	0.0057	-0.0194	0.0029	24171.15	1.9950	100%	0.6595	100%	0.3924	100%
N=687																	
(2) ATV/r or LPV/r	390.41	1.8046											100%		100%		100%
DRV/r	520.50	1.8017	130.10	6.63	117.10	143.10	-0.0029	0.0023	-0.0075	0.0017	Dominated	-1.3010	0%	-0.4356	0%	-0.2625	0%
N=690																	
(3) LPV/r	399.07	1.8037											0.30%		0.30%		0.30%
ATV/r	381.72	1.8055	-17.35	6.69	-30.45	-4.25	0.0018	0.0026	-0.0033	0.0068	-9773.22	0.1767	99.70%	0.0601	99.70%	0.0368	99.70%
N=458																	
(4) ATV/r or LPV/r or DRV/r	433.66	1.8033											0%		0%		0%
DTG	189.56	1.7956	-244.09	4.34	-252.6	-235.58	-0.0077	0.0048	-0.0171	0.0017	31818.65	2.4336	100%	0.8059	100%	0.4803	100%
N=919																	
Comparators	Total Cost	Total QALYs	Incremental cost compared to the next lowest cost				Incremental QALY compared to the next lowest cost				ICER vs. DTG	INHB (λ=100) vs. DTG	Prob of being CE	INHB (λ=300) vs. DTG	Prob of being CE	INHB (λ=500) vs. DTG	Prob of being CE
	mean	mean	mean	SE	95% CI		mean	SE	95% CI								
(5) DTG	189.58	1.7956											100%		100%		100%
ATV/r	380.35	1.8049	190.77	4.76	181.45	200.10	0.0093	0.0059	-0.0022	0.0208	20423.25	-1.9002	0%	-0.6272	0%	-0.3726	0%
LPV/r	399.20	1.8035	18.85	6.22	6.67	31.03	-0.0014	0.0059	-0.0130	0.0101	Dominated	-2.0895	0%	-0.6912	0%	-0.4115	0%
DRV/r	520.41	1.8014	121.21	7.38	106.73	135.68	-0.0021	0.0059	-0.0136	0.0094	Dominated	-3.3023	0%	-1.0968	0%	-0.6557	0%
N=919																	

Notes: 1. All the costs are in 2022 US dollar; 2. Generalized linear model with a gamma distribution of the dependent variable and an identity link function was used to estimate the mean total costs and the incremental costs, controlling for stratification factors; 3. Generalized linear model with a gaussian (normal) distribution of the dependent variable and an identity link function (equivalent to ordinary least squares) was used to estimate the mean total QALYs and the incremental QALYs, controlling for stratification factors and baseline EQ5D indices; 4. Probabilistic sensitivity analyses were conducted to estimate the probability of each intervention being cost effective (reported next to the INHBs).; 5. There are five comparators: (1) DTG vs. ATV/r or LPV/r; (2) DRV/r vs. ATV/r or LPV/r; (3) ATV/r vs. LPV/r; (4) DTG vs. ATV/r or LPV/r or DRV/r. (5) DTG vs. ATV/r vs. LPV/r vs. DRV/r; 6. Cost-effectiveness threshold (λ) of $500 per QALY implied by the decisions that had been previously made on the ART drugs for HIV were used to calculate ICER; 7. For scenario analyses, cost-effectiveness thresholds of $100 and $300 per QALY were also used to calculate INHB. DTG: dolutegravir, ATV/r: ritonavir-boosted atazanavir, LPV/r: ritonavir-boosted lopinavir, DRV/r: ritonavir-boosted darunavir, ART: antiretroviral therapy, NRTI: nucleos(t)ide revirse transcriptase inhibitor, SE: standard error, CI: confidence interval. CE: cost effective, QALY: quality-adjusted life years, ICER: incremental cost-effectiveness ratio**,** INHB: incremental net health benefits.

References

1. Paton NI, Kityo C, Hoppe A, et al. Assessment of second-line antiretroviral regimens for HIV therapy in Africa. N Engl J Med. 2014 Jul 17;371(3):234-47.
2. Paton NI, Musaazi J, Kityo C, et al. Dolutegravir or Darunavir in Combination with Zidovudine or Tenofovir to Treat HIV. N Engl J Med. 2021 Jul 22;385(4):330-341.
3. SECOND-LINE Study Group; Boyd MA, Kumarasamy N, et al. Ritonavir-boosted lopinavir plus nucleoside or nucleotide reverse transcriptase inhibitors versus ritonavir-boosted lopinavir plus raltegravir for treatment of HIV-1 infection in adults with virological failure of a standard first-line ART regimen (SECOND-LINE): a randomised, open-label, non-inferiority study. Lancet. 2013 Jun 15;381(9883):2091-9.
4. Aboud M, Kaplan R, Lombaard J, et al. Dolutegravir versus ritonavir-boosted lopinavir both with dual nucleoside reverse transcriptase inhibitor therapy in adults with HIV-1 infection in whom first-line therapy has failed (DAWNING): an open-label, non-inferiority, phase 3b trial. Lancet Infect Dis. 2019 Mar;19(3):253-264.
5. Ciaffi L, Koulla-Shiro S, Sawadogo A, et al. Efficacy and safety of three second-line antiretroviral regimens in HIV-infected patients in Africa. AIDS. 2015 Jul 31;29(12):1473-81.
6. Division of AIDS (DAIDS) Table for grading the severity of adult and pediatric adverse events v2.1. July 2017. Available at https://rsc.niaid.nih.gov/sites/default/files/daidsgradingcorrectedv21.pdf
7. Antiretroviral therapy for HIV infection in infants and children: towards universal access. Recommendations for a public health approach. 2010 revision. WHO. Available at https://www.who.int/publications/i/item/9789241599801
8. The urgency of now: AIDS at a crossroads. Geneva: Joint United Nations Programme on HIV/AIDS; 2024. Licence: CC BY-NC-SA 3.0 IGO. Available at https://www.unaids.org/en/resources/documents/2024/global-aids-update-2024 . Accessed 14th October 2024.
9. Transforming Vision into Reality: The 2024 Global Alliance Progress Report on Ending AIDS in Children by 2030. Geneva: Joint United Nations Programme on HIV/AIDS; 2024. Licence: CC BY-NC-SA 3.0 IGO. Available at https://www.unaids.org/en/resources/documents/2024/transforming-vision-into-reality . Accessed 14th October 2024.

10. Global and regional trends. UNICEF. Available at https://data.unicef.org/topic/hivaids/global-regional-trends/ . Accessed 14th October 2024.
11. Outcomes of second-line antiretroviral therapy among children living with HIV: a global cohort analysis. Collaborative Initiative for Paediatric HIV Education and Research (CIPHER) Global Cohort Collaboration. J Int AIDS Soc. 2020 Apr;23(4):e25477.
12. Global estimates of viral suppression in children and adolescents and adults on antiretroviral therapy adjusted for missing viral load measurements: a multiregional, retrospective cohort study in 31 countries. Han WM, Law MG, Egger M et al. Lancet HIV. 2021 Dec;8(12):e766-e775.
13. Turkova A, White E, Mujuru HA, et al. Dolutegravir as First- or Second-Line Treatment for HIV-1 Infection in Children. New England Journal of Medicine 2021;385:2531-43.
14. First Pharmacokinetic Data of Tenofovir Alafenamide Fumarate and Tenofovir With Dolutegravir or Boosted Protease Inhibitors in African Children: A Substudy of the CHAPAS-4 Trial. Waalewijn H, Szubert AJ, Wasmann RE et al. Clin Infect Dis. 2023 Sep 18;77(6):875-882
15. Population Pharmacokinetics of Dolutegravir in African Children: Results From the CHAPAS-4 Trial. Waalewijn H, Wasman RE, Bamford A et al. J Pediatric Infect Dis Soc. 2024 Sep 26;13(9):496-500
16. Pharmacokinetics of once-daily darunavir/ritonavir in second-line treatment in African children with HIV. Tsirizani L, Naghani SM, Waalewijn H et al. J Antimicrob Chemother. 2024 Sep 20:dkae319. doi: 10.1093/jac/dkae319
